# Seat-Height Effects During Seated Shoe Donning in Older Adults: A Multi-Stage Ergonomic Evaluation Integrating Muscle Activity, Postural Exposure, and Subjective Comfort

**DOI:** 10.3390/s26206654

**Published:** 2026-10-21

**Authors:** Jingtong Wang, Haowei Wang, Wenxin Kang, Lichun Feng, Zhe Yuan, Mingli Qiang

**Affiliations:** 1 Yunnan Provincial Key Laboratory of Wood and Bamboo Biomass Materials, Southwest Forestry University, Kunming 650224, China; wangjingtong@swfu.edu.cn (J.W.); whw412@swfu.edu.cn (H.W.); kwx@swfu.edu.cn (W.K.); 20231369044@swfu.edu.cn (L.F.); 2 College of Art and Design, Southwest Forestry University, Kunming 650224, China; yuanzhe@swfu.edu.cn

**Keywords:** aging-friendly design, seat height, multi-domain ergonomic evaluation, postural exposure, surface electromyography, seated shoe donning

## Abstract

Inappropriate seat height may affect muscle activity, observational musculoskeletal postural exposure, and subjective comfort during seated shoe donning in older adults. This study characterized seat-height effects across four task stages and explored the combined pattern of these outcomes. Thirty-five community-dwelling older adults with basic self-care ability recruited from a residential community affiliated with Southwest Forestry University (12 men and 23 women; age: 74.60±7.87 years) completed seated shoe donning at 60%, 80%, 100%, and 120% of individual popliteal height. sEMG quantified muscle activity using natural-log-transformed task RMS as the primary analytical measure, with ΔRMS% retained as a secondary quiet-standing-normalized measure; REBA assessed observational postural exposure; subjective comfort was assessed using a study-specific seven-category numerical rating scale; and CRITIC provided an exploratory multi-indicator summary. Primary sEMG inference based on natural-log-transformed task RMS showed seat-height main effects for the BB and the FCU and seat-height × stage interactions for the BB, the FCU, the ES, the RF, and the MG; the LM interaction was not significant. Across the log-RMS mixed-effects, ΔRMS%-based mixed-effects, and log-RMS Gaussian GEE analyses, the BB seat-height main effect and the RF seat-height × stage interaction were the most consistently supported seat-height-related findings, while other muscle-specific effects varied across models. REBA showed a consistent seat-height × stage interaction across analyses, but the overall seat-height main effect was non-significant in the ordinal GEE and significant in the Gaussian GEE sensitivity analysis. Subjective comfort showed consistent seat-height and seat-height × stage effects across the primary and sensitivity analyses. An exploratory CRITIC integration using a within-muscle-standardized log-RMS sEMG summary placed H2 (80% of popliteal height) first among the four tested conditions; the leading position of H2 was retained across alternative sEMG scaling approaches and when the sEMG component was excluded or its orientation reversed, although H2 and H4 nearly tied under the reversed orientation. Overall, seat-height effects varied across task stages and outcome domains. H2 emerged as the highest-scoring tested condition under the present protocol, providing a task-specific basis for future validation.

## 1. Introduction

Activities of daily living (ADLs) provide a standardized framework for describing basic self-care function in older adults [1]. Footwear management is a routine component of everyday self-care. Even for older adults who retain basic ADL independence, seated shoe donning requires coordinated trunk movement, upper-limb manipulation, hand–foot coordination, and postural transitions. These combined demands make it a relevant multi-stage task for ergonomic evaluation. The demands of seated shoe donning vary across task stages. Sitting down, forward shoe manipulation, trunk re-extension, and standing up involve different combinations of lower-limb activity, upper-limb use, and trunk movement. As an important furniture parameter, seat height may therefore affect posture, reach geometry, and transition demands differently across stages. Seating research in older adults suggests that anthropometric fit, functional suitability, and perceived comfort are related but distinct aspects of seating evaluation [2]. Evaluating seat-height effects across the complete task may therefore provide more specific evidence for aging-friendly shoe-donning seat design. From the perspective of muscle activity, seat height can influence muscle-activation patterns during sit-to-stand transitions. Janssen et al. reviewed biomechanical and neuromuscular determinants of sit-to-stand performance and identified seat height as an important task constraint [3]. Yoshioka et al. reported seat-height-related differences in lower-limb joint mechanics during sit-to-stand movements [4], while Roebroeck et al. characterized coordinated lower-limb muscle activity and biomechanics during sit-to-stand transfer [5]. These studies provide a basis for understanding seat-height effects during postural transitions. However, seated shoe donning also involves forward bending and hand–foot coordination, and the muscle-activity pattern during shoe manipulation may differ from that observed during postural transitions. From the perspective of observational postural exposure, Rapid Entire Body Assessment (REBA) can be used to evaluate whole-body observational musculoskeletal postural exposure by considering the trunk, neck, upper limbs, and lower limbs [6]. It has been applied in a range of occupational and functional contexts [7,8,9,10]. Previous studies have reported seat-height-related differences in posture during sit-to-stand transitions [11]. During seated shoe donning, seat height may also affect the relative geometry of the trunk, upper limbs, and feet during forward bending and reaching. Its association with REBA-based postural exposure therefore requires stage-specific evaluation. In addition to muscle activity and observational postural exposure, subjective comfort is an important dimension for evaluating seat-height suitability [12]. In the present study, subjective comfort was treated as a study-specific, stage-specific single-item rating of overall seat-height comfort. Previous seating research has shown that perceived comfort may only partly correspond with objective ergonomic measures [13,14]. Accordingly, muscle activity, postural exposure, and subjective comfort were treated as distinct but complementary outcome domains. CRITIC was selected for exploratory multi-indicator integration because it considers both indicator variability and inter-indicator correlations [15]. This data-driven weighting approach was used to summarize the information carried by the three outcome domains without assigning prespecified subjective weights. Compared with equal weighting, subjective pairwise-weighting approaches such as AHP, and dispersion-only weighting approaches, CRITIC was selected because it is data-driven and explicitly incorporates inter-indicator correlation (information redundancy); this choice does not imply that CRITIC is theoretically superior to alternative integration methods. However, it remains unclear whether seat-height effects observed in isolated sitting or sit-to-stand tasks are consistent across the multiple stages of seated shoe donning. Muscle activity, postural exposure, and subjective comfort may also favor different seat-height conditions. A stage-resolved, multi-domain evaluation is therefore needed to characterize these task-specific differences and trade-offs. The four individualized seat-height levels were spaced at 20% increments of popliteal height to span low, near-popliteal, and high relative seat conditions while accounting for between-person anthropometric differences. Anthropometry-relative seat-height manipulations have been used in chair-rise research [11,16]. Using seat height relative to individual popliteal height also helps account for differences in body size among participants [2,17]. Accordingly, the primary objective of this study was to characterize the stage-dependent effects of four individualized seat-height conditions (60%, 80%, 100%, and 120% of popliteal height) during seated shoe donning in older adults with basic self-care ability who were able to complete the task without assistance. This study examined muscle-specific sEMG responses, REBA-based observational postural exposure, and participant-reported comfort across sitting down, forward bending for shoe donning, seated trunk re-extension, and standing up. A secondary exploratory objective was to summarize the combined pattern of these three evaluation domains by using the CRITIC method. These analyses assessed how seat-height effects differed across task stages and outcome domains. We hypothesized that seat-height effects would differ across task stages, as indicated by a seat-height × stage interaction. No directional hypotheses were specified for individual muscles or stages.

## 2. Materials and Methods

### 2.1. Participants

An a priori power analysis using G*Power version 3.1 [18] for a one-group repeated-measures design with four seat-height conditions indicated a minimum sample size of 24, assuming Cohen’s f=0.25, α=0.05, power =0.80, a within-subject correlation of 0.50, and a nonsphericity correction of ε=1.00. We did not prespecify allowance for attrition or unusable sEMG recordings. Participants were recruited by convenience sampling from a residential community affiliated with the Retirees’ Office of Southwest Forestry University between December 2024 and March 2025. We screened anthropometric characteristics against the 5th–95th percentile range specified in GB/T 10000–2023 [17]. Only right-handed participants were included to reduce laterality-related variability in manual shoe manipulation. Because all participants donned the shoe with the right foot and all sEMG signals were recorded from the right side, restricting the sample to right-handed participants kept hand dominance, task side, and recording side consistent across participants. Of 42 participants initially recruited, seven (16.7%) were excluded because one or more sEMG recordings showed abnormally high amplitudes and markedly irregular waveforms unsuitable for physiological interpretation. Because no quantitative signal-quality threshold or prespecified channel- or trial-level exclusion rule had been established, participant-level complete-case exclusion was applied. Muscle- and trial-specific exclusion logs were not retained, so the affected channels and trials could not be reconstructed retrospectively. The final sample comprised 35 participants (12 men and 23 women; age, 74.60±7.87 years). Participant characteristics are summarized in Table 1. Characteristics of included and excluded participants are compared in Supplementary Table S1. Basic activities of daily living were assessed using the 0–100-point Modified Barthel Index (MBI) described by Shah et al. [19]. An MBI score ≥80 was used as an a priori eligibility threshold for general ADL function and did not imply complete functional independence or independent shoe donning. Shoe-donning ability was assessed separately, and participants were required to complete seated shoe donning and the associated postural transitions without physical assistance. The ≥80 cutoff was a study-defined screening threshold for this protocol rather than a validated threshold for complete independence or independent shoe donning. Eligibility was determined through a pre-experimental screening interview, self-reported medical history, and observed task performance. Exclusion criteria included reported neurological disorders, recent lower-limb injury, previous spinal surgery, recurrent dizziness or unsteadiness, musculoskeletal pain or movement restriction during the experimental movements, or any other condition that could compromise safe task completion. Participants were instructed to avoid strenuous exercise for 24 h before testing. This study was approved by the Ethics Committee of Southwest Forestry University (SWFU-2024-1228). All participants provided written informed consent, and testing was terminated if discomfort or instability occurred.

### 2.2. Materials

**Figure 1:**
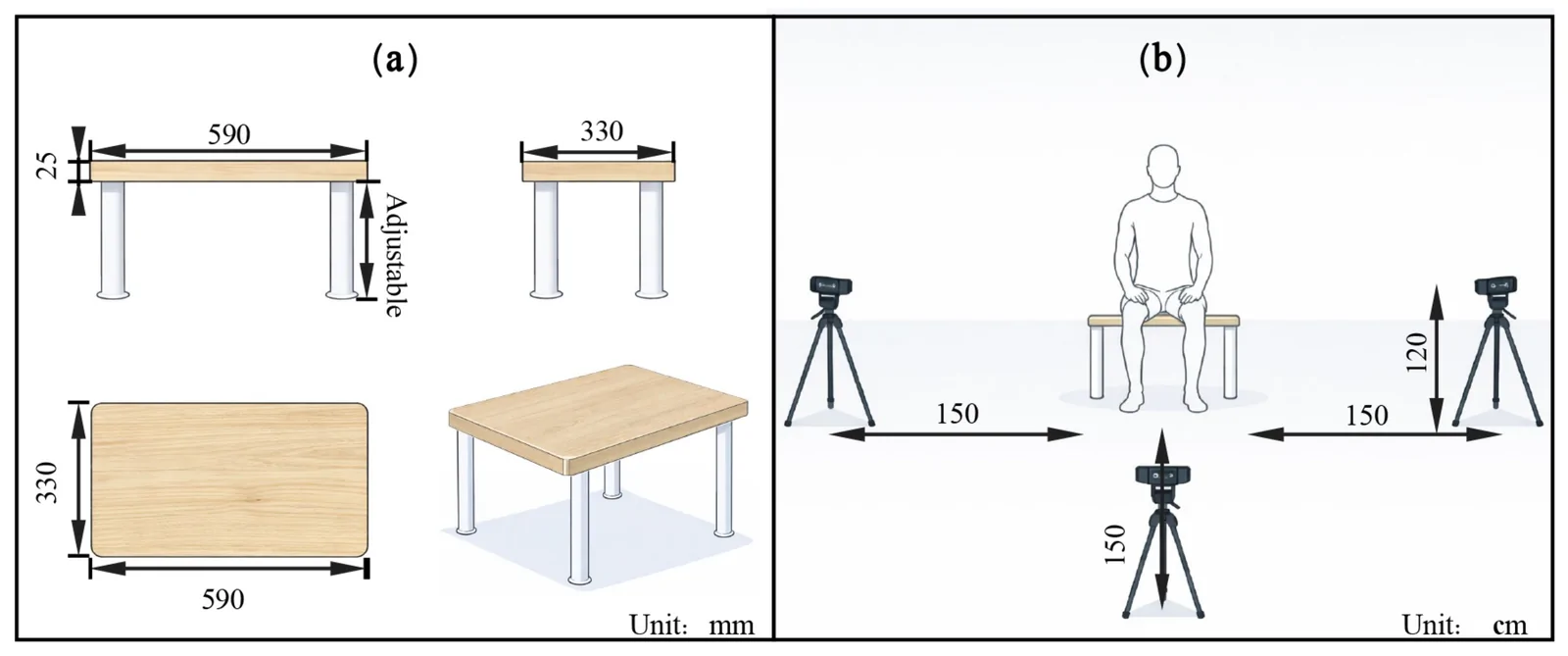
Experimental materials and video-recording setup. (**a**) Schematic of the adjustable shoe-donning chair; (**b**) experimental scene and three-camera layout.

The experiment was conducted in a simulated residential entrance environment using a standardized shoe-donning chair custom-built by the research team at Southwest Forestry University (Kunming, China). The seat was a rigid, unpadded solid-wood board (590×330×25 mm; width × depth × thickness) with a flat horizontal surface, a straight front edge, and no backrest nor armrests. No cushion nor anti-slip covering was used. The measured static coefficient of friction of the seat surface was 0.40–0.55 under the tested contact conditions. Seat height was defined as the vertical distance from the floor to the upper seat surface and individualized according to each participant’s popliteal height (Figure 1a). The same model of low-cut athletic shoe was purchased through an online retail platform in China and used throughout the experiment. The manufacturer information was not available from the retained purchase records. Participants selected the appropriate size. Each shoe had a flexible knitted-mesh upper, a soft outsole, and fixed decorative laces and weighed approximately 300 g. Participants wore their own socks. Shoe donning was performed with the right foot in all trials. Before each trial, the right shoe was positioned 200 mm in front of the seat edge with the opening facing the participant.

### 2.3. Experimental Instruments

Muscle activity was recorded using six ErgoLAB EMG wireless surface electromyography sensors integrated with the ErgoLAB Human–Machine–Environment Synchronization Platform V3.0 (Kingfar International Inc., Beijing, China). Raw sEMG signals were acquired at 1024 Hz. Video was recorded simultaneously using three Logitech C920e webcams (Logitech International S.A., Lausanne, Switzerland) positioned in front of and on the left and right sides of the participant, approximately 1.50 m away and 1.20 m above the floor. Videos were acquired at 1920×1080 pixels and 30 frames/s. Video and sEMG were acquired concurrently within the same ErgoLAB recording session and displayed on a common software timeline. Synchronization was software-based, without an external hardware trigger. The 30-frames/s video provided a nominal temporal resolution of 33.3 ms per frame; absolute synchronization error was not independently validated. The synchronized recordings were used to identify task-stage boundaries and align stage-level video and sEMG data.

### 2.4. Experimental Design and Procedure

#### 2.4.1. Experimental Design

**Figure 2:**
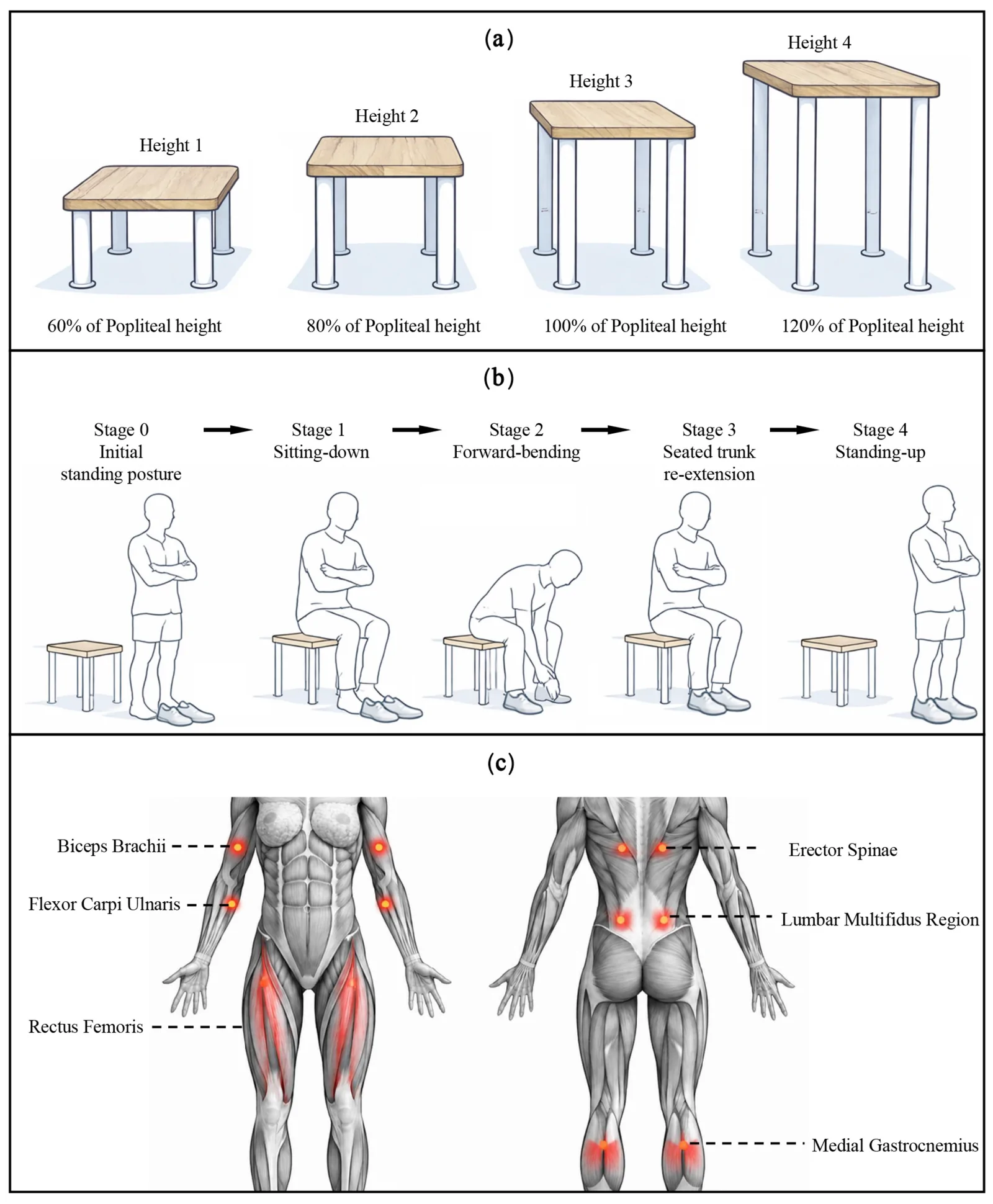
Seat-height conditions, task stages, and sEMG recording sites. (**a**) Four individualized seat-height conditions; (**b**) four stages of the seated shoe-donning task; (**c**) anatomical schematic of the six sEMG recording sites corresponding to those used during data acquisition. Panel (**c**) is an anatomical illustration and not a participant-specific electrode photograph; sites are illustrated bilaterally for orientation, whereas formal sEMG recordings were obtained from the right side only.

A within-subject repeated-measures design was used, with seat height and task stage as within-subject factors. Four individualized seat-height conditions (60%, 80%, 100%, and 120% of each participant’s popliteal height) and four task stages were evaluated (Figure 2a,b). The protocol was partially segmented, with Stages 2 and 3 performed consecutively to preserve the natural transition from shoe donning to trunk re-extension. Detailed stage definitions are provided in Section 2.4.2. The four stages represented distinct task segments and were not directly comparable. Within-stage seat-height effects indicate differences among the four seat-height conditions in the same task context. The seat-height × stage interaction indicates whether the seat-height response differed across task stages. Each of the 35 participants completed two trials at each seat height, and all completed trials were retained. Seat-height order was randomized for each participant by drawing lots without replacement. Within each condition, stages followed the fixed sequence Stage 1 → Stage 2 → Stage 3 → Stage 4. The dependent variables were muscle activity, REBA-based postural exposure, and subjective comfort. Before the formal experiment, a preliminary screening study was conducted in six older adults (three men and three women) to evaluate 12 candidate muscles across the four stages: erector spinae (ES), lumbar multifidus (LM), flexor carpi ulnaris (FCU), latissimus dorsi, medial gastrocnemius (MG), rectus femoris (RF), biceps brachii (BB), vastus lateralis, anterior deltoid, vastus medialis, brachioradialis, and lateral gastrocnemius (Supplementary Table S2). For each muscle, the relative iEMG proportion was calculated as:

(1) $$\eta_{m}=\frac{\sum_{s=1}^{4}I_{m,s}}{\sum_{j=1}^{12}\sum_{s=1}^{4}I_{j,s}}\times 100\%,$$

where $I_{m,s}$ represents the iEMG of the *m*-th candidate muscle during stage *s*. This proportion was used as a descriptive screening index and was not interpreted as a direct estimate of physiological muscle contribution. Final muscle selection considered relative iEMG proportion, task relevance, signal quality, and representative coverage of the upper limbs, trunk, and lower limbs. Six muscles were retained: biceps brachii (BB), flexor carpi ulnaris (FCU), erector spinae (ES), lumbar multifidus (LM), rectus femoris (RF), and medial gastrocnemius (MG) (Figure 2c). During Stage 2, BB and FCU were directly involved in reaching toward the foot, hand positioning, and shoe manipulation. During Stages 1, 3, and 4, the arms remained crossed over the chest; BB and FCU activity in these stages was therefore interpreted primarily as activation associated with maintaining the prescribed arm posture and was not interpreted as purposeful upper-limb task activity. Because arm posture was identical across seat-height conditions within each stage, within-stage seat-height comparisons remained interpretable under the same postural constraint. Cross-stage differences in BB and FCU were not interpreted as differences in task-specific upper-limb demand.

#### 2.4.2. Experimental Procedure

**Figure 3:**
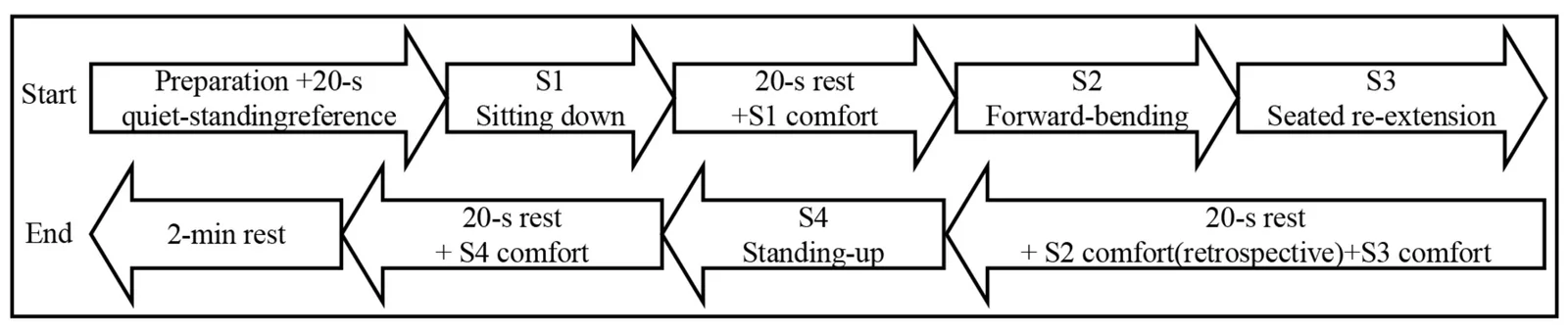
Seated shoe-donning experimental procedure within each seat-height condition. Stage 1 was followed by its comfort rating and a 20 s seated rest. Stages 2 and 3 were performed consecutively; immediately after Stage 3, participants provided the retrospective Stage 2 comfort rating followed by the Stage 3 rating. After a 20 s seated rest, Stage 4 was completed and rated immediately. A 2 min rest separated seat-height conditions.

Prior to electrode placement, hair was shaved when necessary, and the skin was cleaned with medical alcohol and allowed to dry. Disposable adhesive surface electrodes (LT-803A, 36×30 mm; Shanghai Litu Medical Equipment Co., Ltd., Shanghai, China) were placed over the right-side muscle bellies, aligned with the local muscle-fiber direction, with a 25 mm center-to-center inter-electrode distance. Muscle-specific placement followed the SENIAM locations for BB, ES, LM, RF, and MG [20]. The FCU was localized by anatomical palpation over the ulnar proximal forearm and aligned with the local muscle-fiber direction. These placement bases and the corresponding signal-selectivity limitations are identified in Supplementary Table S3. The reference/ground electrode was positioned over or adjacent to a nearby bony landmark away from the target muscle belly. All electrodes were placed by the same trained researcher, and signal quality was verified before recording. A 20 s quiet-standing reference recording was then obtained, and the middle 10 s was used to calculate the reference RMS. For each seat-height condition, participants stood approximately 100 mm in front of the chair with the anterior–posterior foot position standardized; mediolateral spacing and foot orientation were self-selected. Full plantar contact with the floor was maintained in all four seat-height conditions, including the 120% popliteal-height condition. Arms were crossed over the chest during Stages 1, 3, and 4 to standardize the postural transitions and minimize variability arising from upper-limb assistance. During Stage 2, both hands were used naturally because upper-limb involvement was an inherent part of shoe manipulation. The right foot was repositioned as required during Stage 2 for shoe donning, and the prescribed seated starting posture was re-established before Stage 4. The four stages were defined by observable events in the synchronized video: **Stage 1:** From the first observable movement toward sitting to attainment of the prescribed upright seated posture. Comfort was rated immediately, followed by a 20 s rest. **Stage 2:** From the first observable forward trunk or upper-limb movement toward the right shoe to completion of shoe donning in the forward-bent seated posture. **Stage 3:** Immediately following Stage 2, from the first observable trunk re-extension to re-establishment of upright sitting. To preserve the continuous Stage 2–3 transition, Stage 2 comfort was rated retrospectively immediately after Stage 3, followed by the Stage 3 comfort rating. **Stage 4:** After a 20 s seated rest and re-establishment of the prescribed starting posture, from the first observable movement toward standing to attainment of stable upright standing. Comfort was rated immediately afterward. Movement pace was guided using a metronome application running on an Apple iPhone (Apple Inc., Cupertino, CA, USA), with an approximately 3 s target for each stage to standardize movement speed across conditions. Exact stage durations were not quantified as a separate outcome. A 2 min rest separated seat-height conditions. Subjective-comfort assessment is described in Section 2.5.3, and the overall procedure is illustrated in Figure 3.

### 2.5. Data Processing

#### 2.5.1. Surface Electromyography Signal Processing

Raw sEMG signals were acquired at 1024 Hz and processed in ErgoLAB using the software-specified settings: Wavelet Denoise = Medium, 5 Hz high-pass, 500 Hz low-pass, and 50 Hz band-stop filtering. The available software documentation did not specify the filter family, order, cutoff convention, implementation direction, or the internal sequence of the filtering operations; therefore, these parameters were not inferred. After software-based denoising and filtering, signal amplitude was calculated using Moving RMS with a 100 ms sliding window, without separate full-wave rectification [21]:

(2) $$\mathrm{RMS}\sqrt{\frac{\sum_{i=1}^{N}x_{i}^{2}}{N}},$$

where $x_{i}$ is the *i*-th sEMG sample within the RMS window, and *N* is the number of samples in the window. Task-stage boundaries were identified from the synchronized video using the event definitions described in Section 2.4.2. For each muscle and trial, RMS values were extracted over the complete stage interval, including the beginning and end of each movement. The two repeated trials were averaged to obtain one stage-level RMS value for each participant, muscle, seat-height condition, and task stage. The middle 10 s of the 20 s quiet-standing recording were used as the muscle-specific reference. Quiet standing was treated as a standardized reference condition and was not assumed to represent physiologically equivalent resting activation across muscles. Relative RMS change was calculated as:

(3) $$\Delta \mathrm{RMS}\left(\%\right)\frac{{\mathrm{RMS}}_{\mathrm{task}}-{\mathrm{RMS}}_{\mathrm{ref}}}{{\mathrm{RMS}}_{\mathrm{ref}}}\times 100\%,$$

where ${\mathrm{RMS}}_{\mathrm{task}}$ is the stage-level RMS and ${\mathrm{RMS}}_{\mathrm{ref}}$ is the quiet-standing reference. ΔRMS% was used to quantify within-participant, within-muscle changes in muscle activity relative to quiet standing across seat-height and task-stage conditions. For the BB and the FCU, the quiet-standing reference was obtained with the arms in the prescribed reference posture, whereas Stages 1, 3, and 4 required the arms to be crossed over the chest. Therefore, ΔRMS% in these stages included activity associated with maintaining the crossed-arm posture. Negative values indicated task RMS below the quiet-standing reference. Because normalization choice can affect sEMG interpretation [22], absolute reference RMS values are reported in Supplementary Table S4. No MVC or standardized submaximal reference contraction was collected, so ΔRMS% was used only as a within-participant, within-muscle normalized measure and was not interpreted as %MVC or directly comparable physiological demand across muscles or participants. Because of its denominator sensitivity and less favorable model diagnostics, primary sEMG inference used natural-log-transformed task RMS, with ΔRMS% retained as a secondary outcome.

#### 2.5.2. REBA-Based Postural Exposure Assessment

Postural exposure was assessed using Rapid Entire Body Assessment (REBA) [6]. Scoring was based on the sagittal-plane side-view recordings and focused on the postural configurations observed within each predefined task stage. Accordingly, the resulting scores were interpreted as sagittal-plane REBA-based observational postural exposure. REBA scoring included the trunk, neck, legs, upper arms, lower arms, wrists, and applicable task modifiers [6]. No additional load/force, coupling, or activity modifier points were applied in any of the four stages. The experimental task involved no external load or force requiring an added REBA score, and no separate coupling penalty was applicable under the task-scoring procedure. The approximately 3 s metronome-guided target represented a single controlled movement episode rather than prolonged, repetitive, or unstable activity exposure; therefore, no activity modifier was applied. For each trial and task stage, the complete interval between the predefined start and end events was reviewed, and the posture with the highest final REBA score was retained as the trial-level worst posture. If multiple frames yielded the same maximum score, the earliest frame was used for documentation. For each participant × seat-height × stage condition, the higher score from the two valid trials was retained as the condition-level REBA score. Two trained raters independently scored all trials using the same procedure. Inter-rater reliability was evaluated using a two-way random-effects, absolute-agreement ICC for single ratings [ICC(2,1)] with a 95% confidence interval [23]. Discrepant condition-level ratings were jointly reviewed, and the higher score was retained as the final value for analysis. Because seat height and task stage were visible in the recordings, the raters were not blinded to these conditions.

#### 2.5.3. Subjective Comfort

**Figure 4:**
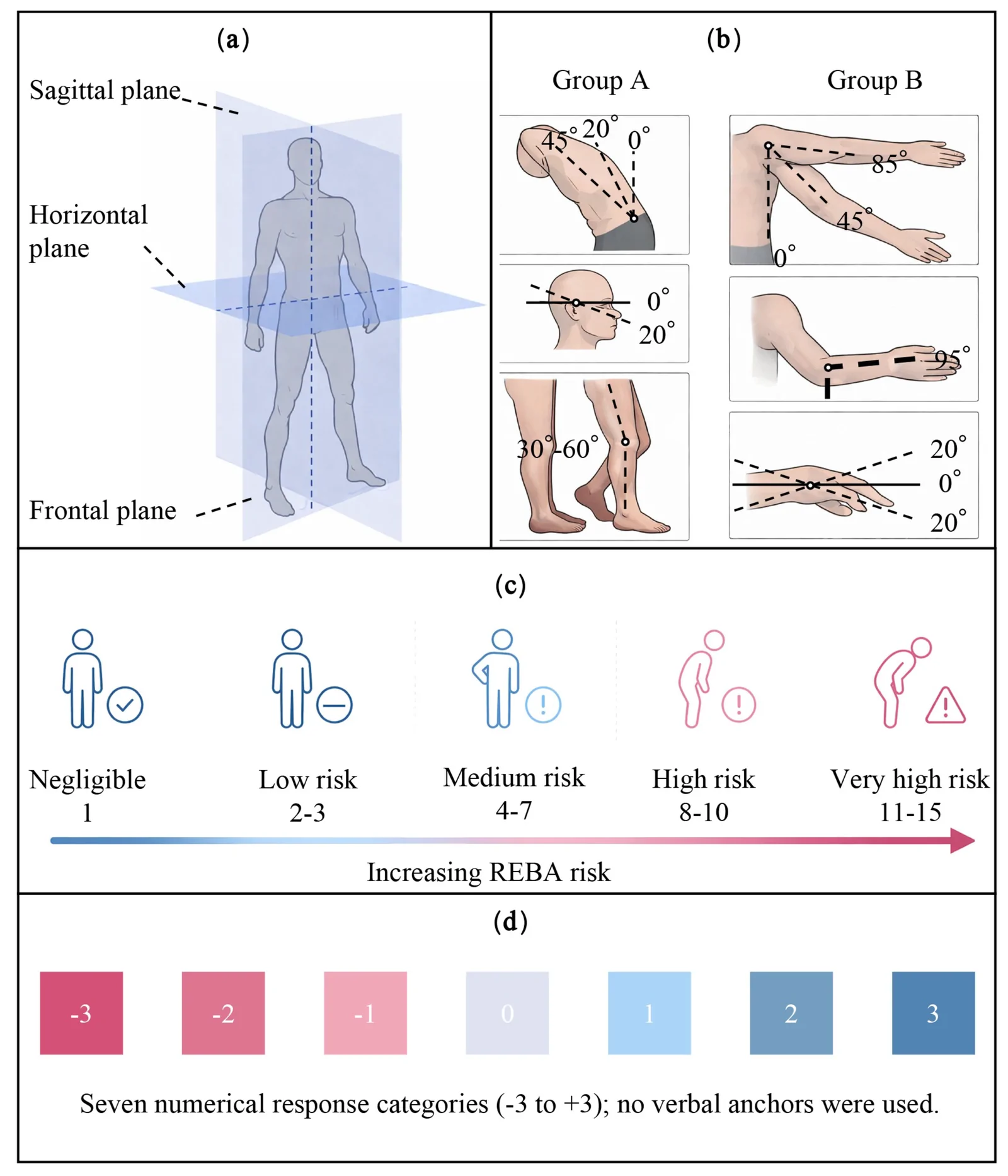
Schematic of REBA scoring and subjective-comfort evaluation. (**a**) Anatomical planes shown for orientation; only the sagittal-plane side view was used for REBA scoring in the present study; (**b**) REBA joint-angle categories and scoring criteria for Groups A and B; (**c**) REBA action/risk categories; (**d**) study-specific seven-category numerical comfort rating (−3 to +3). Angular values represent REBA scoring thresholds; they are not measured three-dimensional joint angles.

Subjective comfort was assessed using a study-specific, stage-specific single-item rating. For each task stage, participants answered the question “How comfortable is the current seat height?” using a seven-category numerical scale ranging from −3 to +3. No verbal labels were assigned to the individual numerical categories. Stage 1 and Stage 4 were rated immediately after completion. To preserve the continuous Stage 2–3 transition, the Stage 2 rating was obtained retrospectively immediately after Stage 3, followed by the Stage 3 rating. Both trial-level ratings were retained for statistical analysis. The REBA scoring framework and the subjective-comfort rating scale are illustrated in Figure 4.

### 2.6. Statistical Analysis

Analyses were performed using IBM SPSS Statistics 27.0 (IBM Corp., Armonk, NY, USA). All tests were two-sided with α=0.05. Detailed model specifications, post hoc comparisons, and multiplicity adjustments are summarized in Supplementary Tables S6 and S7. Primary sEMG inference was based on natural-log-transformed task RMS without quiet-standing normalization. Separate linear mixed-effects models (IBM SPSS MIXED procedure) were fitted for each muscle, with seat height, task stage, and their interaction entered as categorical fixed effects and participant included as a random intercept [24]. Models were estimated using restricted maximum likelihood (REML), and fixed effects were evaluated using Type III *F* tests. Holm adjustment was applied across the six muscles separately within the seat-height, stage, and seat-height × stage effects families [25]. Within-stage seat-height effects were used to assess differences among the four seat-height conditions in the same task context. The seat-height × stage interaction was used to determine whether the magnitude or direction of the seat-height effect differed across task stages. Because the stages represented distinct motor actions and procedural conditions, the stage main effect was not emphasized in the substantive interpretation. For muscles with a significant seat-height × stage interaction, stage-specific simple effects of seat height were examined within the primary log-RMS mixed-effects model. Stage-specific *p*-values were Holm-adjusted across the four stages within each muscle; within significant stages, the six pairwise seat-height contrasts were also Holm-adjusted. Exponentiated pairwise contrasts are reported as task-RMS ratios. ΔRMS%-based mixed-effects models were retained as a secondary reference-normalized analysis. Participant-clustered Gaussian GEE models of log-transformed task RMS with robust standard errors were used as an additional robustness analysis. Model diagnostics included residual distribution, variance patterns, standardized residuals, convergence, and leave-one-participant-out influence checks. Findings supported across analytical approaches were considered more robust, while effects that varied across models were treated as analysis-dependent. Although log transformation improved residual distribution and variance behavior, residual heterogeneity remained across some height × stage cells. A participant-clustered Gaussian GEE with robust sandwich standard errors therefore provided a complementary variance-robust assessment of the fixed-effects conclusions. REBA and subjective comfort were analyzed as ordinal outcomes using participant-clustered cumulative-logit GEE models with exchangeable working correlation structures and robust standard errors [26]. For REBA, the higher score from the two trials was retained for each participant × seat-height × stage condition, yielding 560 condition-level observations. For subjective comfort, both trial-level ratings were retained, yielding 1120 observations, with trial number included as an additional predictor. Model convergence was verified. Because cumulative-logit models assume proportional odds, this assumption was examined using participant-clustered cumulative binary GEE models across the observed ordinal cut-points. For REBA, sparse or empty condition-by-score cells produced instability in several threshold-specific models, so the proportional-odds assumption was not regarded as firmly established. A Gaussian GEE was therefore used as an alternative outcome-specification sensitivity analysis for REBA. For subjective comfort, a Gaussian GEE and a collapsed three-category ordinal GEE were used as additional sensitivity analyses. The Gaussian GEE provided an alternative mean-scale outcome specification that retained participant clustering and robust sandwich standard errors without imposing the proportional-odds constraint. Effects supported across the ordinal and Gaussian specifications were regarded as robust to outcome specification, whereas discordant effects were treated as model-dependent. Significant seat-height × stage interactions in the primary ordinal models were followed by stage-specific simple effects and Holm-adjusted pairwise seat-height comparisons, with odds ratios (ORs) and 95% confidence intervals reported. CRITIC was used for exploratory integration of the three measured outcome domains across the 16 seat-height × stage conditions [15]. For each muscle, condition-level log-transformed task RMS was standardized across the 16 conditions, and the six standardized values were averaged to obtain a standardized sEMG summary. This summary was used as a mathematical aggregation of the recorded muscle signals and was not interpreted as a physiologically equivalent measure of total muscle load or physiological demand. The standardized sEMG summary and REBA were coded as cost-type indicators, whereas subjective comfort was coded as a benefit-type indicator. This coding was used only to align the mathematical orientation of the composite score and did not imply that lower muscle activation was inherently physiologically preferable. To assess dependence of the composite ordering on the sEMG directional convention, two additional sensitivity analyses were performed: (1) the sEMG component was excluded, and CRITIC weights were recalculated using only REBA and subjective comfort; (2) the sEMG orientation was reversed, and the complete CRITIC calculation, including indicator weights and composite scores, was repeated. Direction-specific min–max normalization was performed as follows:

(4) $$z_{ij}=\left\{\begin{array}{cc} \frac{x_{ij}-\min\left(x_{j}\right)}{\max\left(x_{j}\right)-\min\left(x_{j}\right)},\; & \mathrm{for}\;\mathrm{benefit}-\mathrm{type}\;\mathrm{indicators},\\ \frac{\max\left(x_{j}\right)-x_{ij}}{\max\left(x_{j}\right)-\min\left(x_{j}\right)},\; & \mathrm{for}\;\cos t-\mathrm{type}\;\mathrm{indicators}. \end{array}\right.$$

where $x_{ij}$ is the original value of indicator *j* for condition *i* and $z_{ij}$ is the corresponding normalized value. The information content and CRITIC weight of indicator *j* were then calculated as:

(5) $$C_{j}=\sigma_{j}\sum_{k=1}^{m}\left(1-r_{jk}\right),\;w_{j}=\frac{C_{j}}{\sum_{k=1}^{m}C_{k}},$$

where $\sigma_{j}$ is the standard deviation of normalized indicator *j*, $r_{jk}$ is the Pearson correlation between indicators *j* and *k*, $C_{j}$ is the information content of indicator *j*, $w_{j}$ is its CRITIC weight, and *m* is the number of indicators. The composite score for condition *i* was calculated as:

(6) $$S_{i}=\sum_{j=1}^{m}w_{j}z_{ij},$$

where $S_{i}$ is the composite condition score. The overall score for each seat-height condition was calculated as the equal-weight average of the four stage-specific scores. CRITIC scores were interpreted as sample-dependent composite summaries of the three measured outcome domains. Sensitivity analyses included the original ΔRMS%-based muscle component, a min–max-standardized log-RMS component, a rank-based sEMG summary, alternative stage-weighting schemes, and stage-omission analyses, as well as exclusion of the sEMG component and reversal of the sEMG indicator orientation. Stability of the ordering was interpreted as numerical robustness of the exploratory composite and did not establish physiological validity.

## 3. Results

### 3.1. Muscle Activity

#### 3.1.1. Primary Log-RMS Mixed-Effects Model Results

Table 2 summarizes the primary mixed-effects analyses of natural-log-transformed task RMS. Significant seat-height main effects were observed for BB and FCU, while significant seat-height × stage interactions were identified for BB, FCU, ES, RF, and MG; the interaction was not significant for LM. Full Type III test statistics, including the task-stage main effects, are reported in Table 2.

#### 3.1.2. Consistency Across sEMG Analyses and Stage-Specific Results

The pattern of seat-height-related sEMG findings differed across analyses. In the secondary ΔRMS%-based mixed-effects models, seat-height main effects were significant for the BB and the MG, whereas the seat-height × stage interaction was significant only for the RF. In the primary log-RMS mixed-effects models, seat-height main effects were significant for the BB and the FCU, and interactions were significant for the BB, the FCU, the ES, the RF, and the MG. In the log-RMS Gaussian GEE analysis, seat-height main effects were significant for the BB, the FCU, the LM, and the MG, and seat-height × stage interactions were significant for all six muscles. The BB seat-height main effect and the RF seat-height × stage interaction were the only seat-height-related effects reproduced across all three analyses. Other muscle-specific effects varied across analyses. Stage-specific simple-effects analyses within the primary log-RMS model showed significant seat-height effects for the BB in S1, S3, and S4; the FCU in S1 and S4; the ES in S2 and S3; the RF in S1 and S2; and the MG in S1, S3, and S4 after Holm adjustment across the four stages. Holm-significant pairwise seat-height contrasts are reported as task-RMS ratios in Supplementary Table S6. In the secondary ΔRMS%-based model, follow-up analysis of the significant RF interaction showed a significant seat-height effect in Stage 1. H1 exceeded H3 (mean difference = 92.65 percentage points, 95% CI 44.04–141.27, Holm-adjusted p=0.001) and H4 (104.54 percentage points, 95% CI 55.92–153.16, Holm-adjusted p<0.001).

#### 3.1.3. Model Diagnostics

Negative ΔRMS% values occurred in 703 of 3360 observations (20.9%). The secondary ΔRMS%-based models showed marked residual skewness, variance heterogeneity, and several extreme standardized residuals. All primary log-RMS mixed-effects models and log-RMS Gaussian GEE models converged. The primary log-RMS mixed-effects models showed more favorable diagnostics: residual skewness ranged from 0.05 to 0.55, max/min residual-variance ratios ranged from 2.39 to 4.13, and 0–6 standardized residuals exceeded |3| across muscles; maximum leave-one-participant shifts were <0.82 SE. The small number of standardized residuals exceeding |3| and the modest leave-one-participant shifts suggest that the remaining heterogeneity was not primarily driven by isolated extreme observations or a single influential participant. Detailed diagnostics for both the primary log-RMS and secondary ΔRMS%-based mixed-effects models and additional sEMG analysis results are reported in Supplementary Table S6. The residual-variance ratios indicate that log transformation reduced but did not eliminate heterogeneity across the 16 height × stage cells. Under the participant-clustered Gaussian GEE with robust standard errors, the BB seat-height main effect remained significant [Wald $\chi^{2}\left(3\right)=68.38$, Holm-adjusted p<0.001], and the RF seat-height × stage interaction remained significant [Wald $\chi^{2}\left(9\right)=81.49$, Holm-adjusted p<0.001]. These two effects were also the only seat-height-related findings reproduced across all three sEMG analysis frameworks.

### 3.2. REBA-Based Postural Exposure

Inter-rater reliability across the 560 paired pre-consensus condition-level REBA ratings was high, with an ICC(2,1) of 0.976 (95% CI: 0.971–0.979). The two raters assigned identical scores in 496 of 560 rating pairs (88.6%), and 559 pairs (99.8%) differed by no more than one REBA point. The participant-clustered cumulative-logit GEE converged successfully (exchangeable working correlation = 0.014) and showed no significant overall main effect of seat height [Wald $\chi^{2}\left(3\right)=7.34$, p=0.062], while task stage [Wald $\chi^{2}\left(3\right)=209.95$, p<0.001] and the seat-height × stage interaction [Wald $\chi^{2}\left(9\right)=121.90$, p<0.001] were significant. Because the task stages were not equivalent experimental units, interpretation focused on the seat-height × stage interaction and within-stage seat-height comparisons. Stage-specific simple effects of seat height were significant in all four stages after Holm adjustment (all adjusted p<0.001); detailed pairwise comparisons are reported in Supplementary Table S7. The exact-score distribution was sparse within several height × stage conditions: a total of 66 of 128 condition-by-score cells were empty, and 96 contained fewer than five observations. Threshold-specific cumulative binary GEE diagnostics showed instability at sparse cut-points, so the proportional-odds assumption was not considered firmly established for REBA. In a Gaussian GEE sensitivity analysis, task stage [Wald $\chi^{2}\left(3\right)=1364.84$, p<0.001] and the seat-height × stage interaction [Wald $\chi^{2}\left(9\right)=168.82$, p<0.001] remained significant, whereas the overall seat-height main effect became significant [Wald $\chi^{2}\left(3\right)=26.37$, p<0.001]. Thus, the seat-height × stage interaction was consistently supported across the two model specifications, whereas the overall seat-height main effect was model-dependent. Accordingly, the retained REBA conclusion is the cross-specification seat-height × stage interaction, whereas the overall seat-height main effect remains model-dependent. Descriptively, higher seat conditions generally showed lower REBA scores in Stages 1, 3, and 4, whereas the opposite pattern was observed in Stage 2. Condition-level descriptive statistics and diagnostic/sensitivity results are reported in Supplementary Tables S5 and S7.

### 3.3. Subjective Comfort

**Figure 5:**
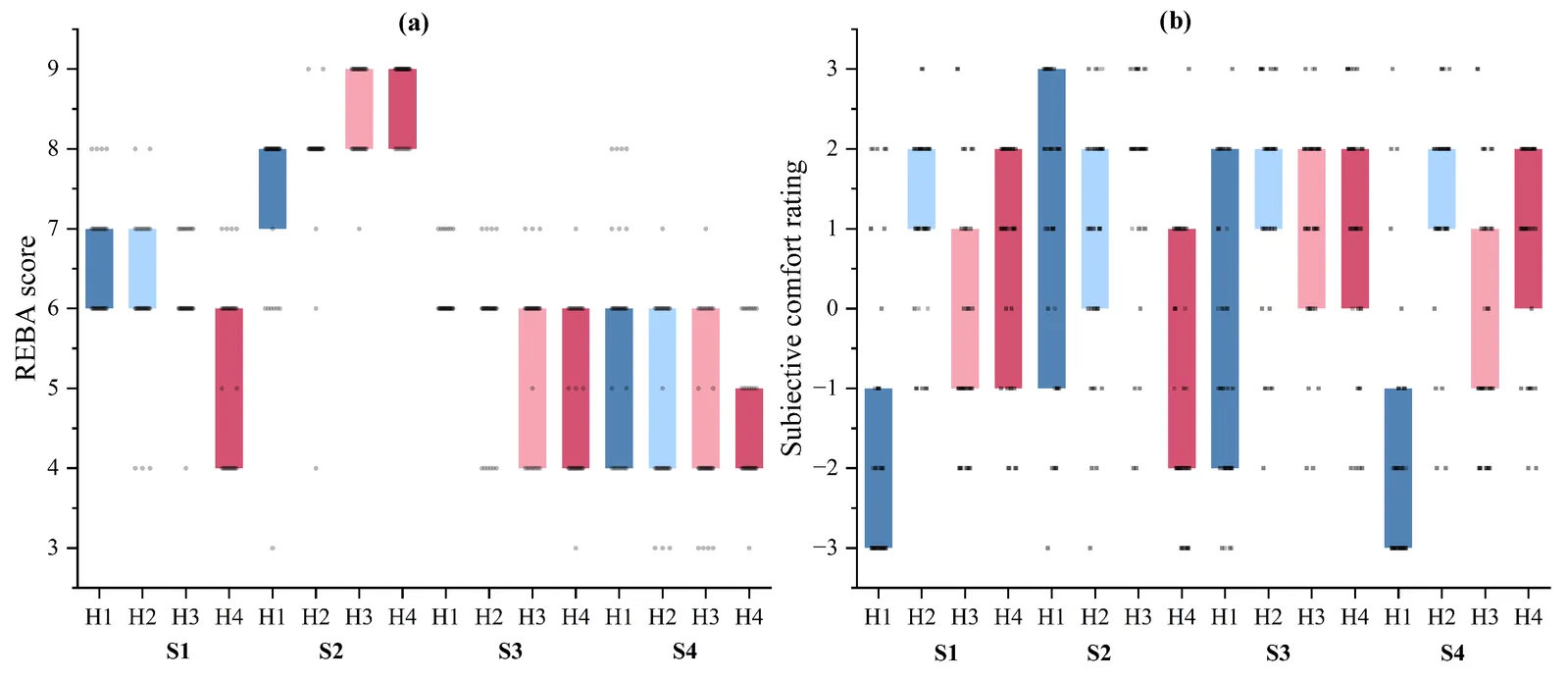
Distributions of REBA-based postural exposure and subjective comfort across seat-height and task-stage conditions. (**a**) REBA scores; (**b**) subjective-comfort ratings. Boxes indicate the interquartile range, center lines indicate medians, and jittered points represent individual observations.

The participant-clustered cumulative-logit GEE converged successfully (exchangeable working correlation = 0.100) and showed significant effects of seat height [Wald $\chi^{2}\left(3\right)=41.78$, p<0.001], task stage [Wald $\chi^{2}\left(3\right)=23.05$, p<0.001], and the seat-height × stage interaction [Wald $\chi^{2}\left(9\right)=69.49$, p<0.001]. Trial number was not significant [Wald $\chi^{2}\left(1\right)=0.60$, p=0.437]. The principal comfort findings were retained under alternative outcome specifications. The Gaussian GEE retained significant seat-height [Wald $\chi^{2}\left(3\right)=82.73$, p<0.001], task-stage [Wald $\chi^{2}\left(3\right)=26.93$, p<0.001], and interaction effects [Wald $\chi^{2}\left(9\right)=159.72$, p<0.001], with trial remaining non-significant (p=0.442). A collapsed three-category ordinal GEE likewise retained significant seat-height [Wald $\chi^{2}\left(3\right)=42.89$, p<0.001], task-stage [Wald $\chi^{2}\left(3\right)=12.12$, p=0.007], and interaction effects [Wald $\chi^{2}\left(9\right)=63.06$, p<0.001], with trial remaining non-significant (p=0.115). Agreement across all three specifications supports the retained seat-height and seat-height × stage effects for subjective comfort. Stage-specific simple effects of seat height were significant in all four stages after Holm adjustment (all p<0.001). Pairwise comparisons showed similar patterns in Stages 1 and 4, where H2 and H4 received higher comfort ratings than H1 and H3, with no significant difference between H2 and H4. In Stage 3, H1 was rated lower than H2, H3, and H4, whereas these three conditions did not differ significantly. Stage 2 showed a different pattern: H4 received lower ratings than H1, H2, and H3, and H3 was also rated higher than H2. Full pairwise contrasts and diagnostic/sensitivity results are reported in Supplementary Table S7. Distributions of REBA-based postural exposure and subjective comfort across seat-height and task-stage conditions are shown in Figure 5.

### 3.4. Exploratory CRITIC Evaluation

#### 3.4.1. Indicator Weights

Using the within-muscle-standardized log-RMS sEMG summary, the CRITIC analysis assigned weights of 30.12% to the standardized sEMG summary, 36.10% to REBA-based postural exposure, and 33.78% to subjective comfort. These coefficients reflect the variability and intercorrelation structure of the present condition-level data and were not interpreted as relative physiological, clinical, or ergonomic importance.

#### 3.4.2. Stage-Specific and Overall Rankings

**Figure 6:**
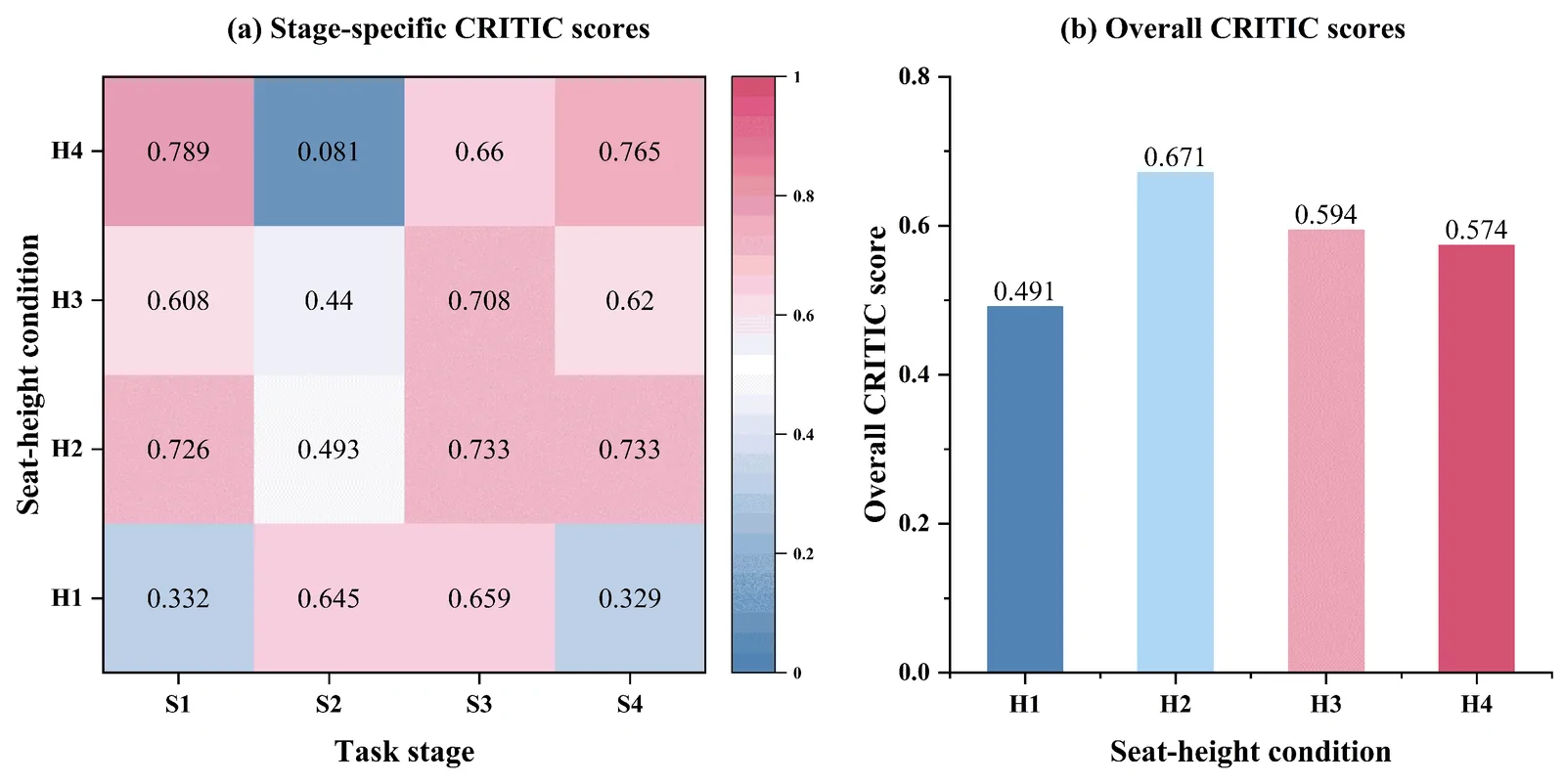
CRITIC composite scores using the within-muscle-standardized log-RMS sEMG summary. (**a**) Stage-specific scores for the 16 seat-height × stage conditions; (**b**) overall scores obtained by equally averaging the four stage-specific scores for each seat height. Higher values indicate higher composite scores under the exploratory CRITIC coding.

Stage-specific CRITIC scores differed across seat-height conditions (Figure 6a). The descending score order was H4 > H2 > H3 > H1 in Stage 1, H1 > H2 > H3 > H4 in Stage 2, H2 > H3 > H4 > H1 in Stage 3, and H4 > H2 > H3 > H1 in Stage 4. After equally averaging the four stage-specific scores, the overall CRITIC scores were 0.4911 for H1, 0.6713 for H2, 0.5941 for H3, and 0.5738 for H4 (Figure 6b). H2 had the highest composite score under the prespecified exploratory coding, followed by H3, H4, and H1. The composite scores summarize the joint pattern of the three measured outcome domains under the exploratory CRITIC coding. They were interpreted as sample-dependent mathematical summaries and did not represent direct measures of physiological demand or overall ergonomic performance.

#### 3.4.3. Sensitivity Analyses

The overall ordering H2 > H3 > H4 > H1 was retained when the muscle-activity component was alternatively constructed from the original ΔRMS% values, from min–max-standardized log-RMS values, and from a within-participant rank-based sEMG summary. This agreement indicates numerical robustness of the whole-task composite ordering across the tested sEMG scaling approaches. Physiological validity and ergonomic superiority require separate evidence. Under alternative stage-weighting schemes, H2 remained the highest when each stage was assigned a weight of 0.40 in turn and the remaining stages 0.20 each. H2 also remained the highest when Stage 1, 3, or 4 was omitted, whereas omission of Stage 2 changed the leading condition to H4. Thus, the overall ranking was stable across the tested sEMG scaling approaches and moderate stage reweighting, while the leading condition depended on the inclusion of Stage 2. Full sensitivity results are reported in Supplementary Table S8. When sEMG was excluded, the overall ordering changed to H2 > H4 > H3 > H1. Reversing the sEMG orientation produced the same ordering, with H2 and H4 nearly tied. Thus, H2 remained the leading condition in both scenarios, whereas the separation between H2 and H4 and the H3/H4 ordering were sensitive to the treatment of the sEMG component.

## 4. Discussion

### 4.1. Main Findings

Seat-height responses varied across task stages and outcome domains. For sEMG, the BB seat-height main effect and RF seat-height × stage interaction were the most consistently supported findings across the three analyses, while other muscle-specific effects varied across models. REBA showed a consistent seat-height × stage interaction, whereas its overall seat-height main effect depended on model specification. Subjective comfort showed consistent seat-height and seat-height × stage effects across analyses. Because the four task stages represented distinct motor actions, interpretation focused on within-stage seat-height differences and variation in seat-height response patterns across stages. The findings show that the same seat-height manipulation can produce different responses across components of a multi-stage functional self-care task and across muscle activity, REBA-based postural exposure, and subjective comfort. This supports evaluating the complete task and multiple outcome domains, while the specific response patterns observed here remain dependent on the present sample and standardized protocol.

### 4.2. Stage-Dependent Patterns of Seat-Height Responses

The present findings suggest that the effect of seat height depends on the functional demands of each task segment. During postural-transition stages, higher seat conditions generally corresponded to lower REBA-based postural exposure, whereas the forward shoe-manipulation stage showed a different pattern. This contrast indicates that a seat height that facilitates postural transitions may not produce the same response during forward reaching and shoe manipulation. Seat-height suitability therefore appears to reflect a balance among the different demands encountered across the complete task. Previous studies provide context for the stage-dependent patterns observed here. Seat height can influence movement performance and posture during sit-to-stand and stand-to-sit tasks in older adults [27,28,29,30], whereas seated reaching involves different trunk and lower-limb configurations [31,32]. These task-specific movement demands may help explain why seat-height responses differed across stages. Because three-dimensional kinematics, joint forces or moments, and center-of-mass displacement were not measured, the underlying biomechanical mechanisms remain uncertain. Stage-specific procedures also need to be considered when interpreting these patterns. Participants used both arms naturally during Stage 2 but maintained a crossed-arm posture during Stages 1, 3, and 4, which contributed to between-stage differences in REBA-based postural exposure. The CRITIC sensitivity analysis also showed that inclusion of the shoe-manipulation stage affected the whole-task composite ordering. Together, these findings support evaluating seat height across the complete multi-stage activity, because conclusions based only on an isolated postural transition may not fully represent the demands of seated shoe donning. Because Stage 2 comfort was rated retrospectively immediately after Stage 3, between-stage comfort comparisons were interpreted cautiously; within-Stage 2 seat-height comparisons remained internally consistent because the same rating procedure was used across all four seat-height conditions.

### 4.3. Muscle- and Stage-Specific sEMG Responses to Seat Height

Interpretation of the sEMG findings focused on effects reproduced across analytical approaches. The BB seat-height main effect and RF seat-height × stage interaction were the most consistent findings, whereas several other muscle-specific effects were more sensitive to normalization and model choice. For the RF, the higher Stage 1 activity at H1 than at H3 and H4 can be interpreted in the context of prior transition studies. Jeon et al. [33] characterized rectus femoris activation during stand-to-sit movement in older adults. Seat-height-related changes in lower-limb muscle activity have also been reported in sit-to-stand and toilet-rise studies [34,35]. Upper-limb responses require stage-specific interpretation because the BB and the FCU contributed directly to reaching and shoe manipulation during Stage 2, but mainly maintained the prescribed crossed-arm posture during Stages 1, 3, and 4. Thus, their within-stage responses provide the more informative basis for interpretation. Physiological interpretation remains limited by the recording and normalization procedures. Without MVC or standardized submaximal reference contractions, sEMG amplitudes cannot be interpreted as %MVC, muscle force, or directly comparable physiological demand across muscles or participants. Surface recordings over the lumbar multifidus region may include adjacent erector spinae activity [36,37], and FCU selectivity may also be affected by neighboring muscles. More generally, sEMG amplitude is influenced by electrode placement, recording conditions, and normalization procedures [38].

### 4.4. Cross-Domain Trade-Offs and Exploratory Integration

Muscle activity, REBA-based postural exposure, and subjective comfort showed different response patterns to seat height, indicating that these outcome domains captured complementary aspects of the task. This finding is consistent with previous seating research showing that perceived comfort may only partly correspond with posture or muscle activity [13,14] and with studies in older adults showing that functional performance and perceived comfort can respond differently to seating characteristics [2,39]. In the present multi-stage shoe-donning task, these differences suggest that seat-height suitability cannot be characterized adequately by a single physiological, postural, or perceptual measure. The individual outcome domains therefore remain central to interpreting how seat height affects the task. The exploratory CRITIC integration provided a whole-task summary of these cross-domain trade-offs. The relatively balanced weights across the standardized sEMG summary, REBA-based postural exposure, and subjective comfort indicate that the composite score was informed by all three measured domains under the present dataset. Importantly, the overall seat-height ordering remained unchanged when the muscle-activity component was constructed using log-RMS, the original ΔRMS%, or a rank-based summary. This stability suggests that the composite ordering was not driven solely by the quiet-standing normalization used for ΔRMS%. At the same time, averaging standardized activity across anatomically and functionally different muscles simplifies their distinct physiological roles. CRITIC is therefore most useful here as an exploratory synthesis of multi-domain trade-offs, while muscle-specific sEMG, REBA-based postural exposure, and subjective comfort provide the basis for substantive interpretation. The direction-sensitivity analyses further showed that H2 remained the leading condition when the sEMG component was excluded or reversed, although the reversed orientation nearly led to a tie between H2 and H4 and changed the H3/H4 order. This indicates greater stability of the leading condition than of the detailed ranking.

### 4.5. Implications for Aging-Friendly Shoe-Donning Seat Design

Among the four seat-height conditions tested, H2 (80% of popliteal height) had the highest exploratory whole-task composite score. This finding identifies the tested 80% popliteal-height condition as a candidate for future validation under comparable user and task conditions. However, the stage-specific results also showed that the same seat height did not produce uniformly favorable responses during postural transitions and forward shoe manipulation. Seat-height design for this activity should therefore consider the complete shoe-donning sequence and multiple outcome domains. In the present sample, H2 corresponded to approximately 330 mm based on a mean popliteal height of 411.9 mm. This value is sample-specific and provides a reference for future validation. Previous studies have shown that seat-height preferences and functional responses in older adults depend on the activity being performed [40]. Translation of the present anthropometry-relative finding into fixed, multiple-height, or adjustable seating therefore requires prospective evaluation in the intended user population, with consideration of anthropometric variation, functional characteristics, and natural shoe-donning strategies.

### 4.6. Limitations and Future Directions

Several limitations should be considered. First, the sample consisted of community-dwelling, right-handed older adults with basic self-care ability, and we did not assess detailed functional characteristics using standardized measures. In addition, seven participants were excluded because of unusable sEMG recordings without prespecified quantitative signal-quality thresholds. These factors limit generalizability, and future studies should include broader populations and prospectively defined signal-quality criteria. Second, the highly standardized protocol improved within-study comparability but reduced ecological validity. Prescribed arm postures, metronome-guided pacing, a fixed stage sequence, and the partially segmented task differed from natural shoe-donning behavior. Because the stages also differed in motor action and procedural conditions, the present findings are most informative for within-stage seat-height comparisons and variation in seat-height responses across task-stage conditions. Third, interpretation is constrained by measurement and analytical limitations. The absence of MVC or a standardized submaximal reference limits physiological interpretation of sEMG amplitude, and some sEMG findings varied across analytical approaches. REBA-based postural exposure was based on sagittal-plane observations, and the study-specific subjective-comfort item has not been independently validated. Sparse REBA score distributions prevented firm evaluation of the proportional-odds assumption. The seat-height × stage interaction persisted under the Gaussian GEE, whereas the overall seat-height main effect remained model-dependent. Log transformation also reduced but did not eliminate residual-variance heterogeneity in the primary sEMG mixed-effects models; the BB seat-height main effect and RF seat-height × stage interaction remained supported under the robust Gaussian GEE. Finally, CRITIC remains an exploratory, sample-dependent whole-task integration. The composite score simplifies information across different muscles and outcome domains, and its ordering was sensitive to stage composition. Direction/exclusion sensitivity analyses also showed that the detailed CRITIC ordering was more sensitive than the leading condition. Only four discrete seat heights were tested. Broader populations, more naturalistic tasks, and independently defined seat-height conditions are therefore needed before practical design guidance can be established.

## 5. Conclusions

Stage-dependent patterns of seat-height effects were observed across muscle activity, REBA-based postural exposure, and subjective comfort, with the forward shoe-manipulation segment showing a different response pattern from several postural-transition stages. Across the three sEMG analytical approaches, the BB seat-height main effect and the RF seat-height × stage interaction were the most consistently supported findings. In contrast, other muscle-specific effects varied across models. In the exploratory whole-task CRITIC analysis, H2 (80% of popliteal height) had the highest composite score among the four tested conditions, and its leading position was retained under the tested alternative sEMG scaling approaches. Excluding the sEMG component or reversing its orientation also retained H2 as the highest-scoring condition; under the reversed orientation, H2 and H4 nearly tied and the lower-ranked order changed. These findings support evaluating seat height across the complete multi-stage task and multiple outcome domains. H2 represents the highest-scoring tested condition under the present sample and protocol and requires validation in more naturalistic tasks and broader older populations before practical design application. 

## Figures and Tables

**Table 1:** Participant characteristics (n=35).

Participant Group	Age (Years)	Height (m)	Body Weight (kg)	Popliteal Height (cm)	MBI Score
Total (n=35)	74.60±7.87	1.58±0.07	62.09±9.73	41.19±1.76	86.13±6.01
Male (n=12)	76.00±9.63	1.65±0.05	67.92±10.34	42.86±1.29	87.62±6.48
Female (n=23)	73.87±6.91	1.55±0.05	59.06±8.04	40.32±1.28	85.36±5.75

*Note:* Values are presented as means ± SDs. Popliteal height was measured vertically from the floor to the lower edge of the popliteal fossa with the participant seated and the hips, knees, and ankles at approximately $90^{\circ }$. MBI, Modified Barthel Index.

**Table 2:** Type III fixed-effects tests for seat height, task stage, and their interaction in the primary natural-log-transformed task-RMS linear mixed-effects models.

Muscle	Seat Height F(3,510)	Adjusted *p*	Stage F(3,510)	Adjusted *p*	Height × Stage F(9,510)	Adjusted *p*
BB	26.03	<0.001	233.27	<0.001	4.49	<0.001
FCU	8.16	<0.001	90.10	<0.001	3.99	<0.001
ES	0.92	0.865	64.40	<0.001	3.99	<0.001
LM	3.22	0.091	7.02	<0.001	1.50	0.145
RF	0.16	0.925	60.22	<0.001	6.07	<0.001
MG	2.97	0.095	11.21	<0.001	3.89	<0.001

*Note:* Separate models were fitted for the six muscles. *p*-Values were Holm-adjusted across the six muscles separately within the seat-height, stage, and seat-height × stage omnibus effects families.

## Data Availability

De-identified supporting data for the primary study outcomes are provided in Supplementary Data S1. The dataset includes participant-level sEMG outcome data, trial-level subjective-comfort ratings, and condition-level REBA summaries corresponding to the reported analyses. Raw sEMG signals, video recordings, original rater-level REBA records, and the complete preprocessing dataset are not publicly available because of ethical and participant-privacy considerations. Additional de-identified data supporting the findings of this study are available from the corresponding author upon reasonable request, subject to applicable ethical and privacy restrictions.

## Supplementary Materials

The following supporting information can be downloaded at https://www.mdpi.com/article/10.3390/s26206654/s1, Supplementary Data S1 provides de-identified supporting data for the primary study outcomes, including participant-level sEMG outcome data, trial-level subjective-comfort ratings, and condition-level REBA summaries. Supplementary Table S1. Baseline characteristics of included and excluded participants. Supplementary Table S2. Preliminary screening of the 12 candidate muscles. Supplementary Table S3. Anatomical landmarks for sEMG electrode placement. Supplementary Table S4. Distribution of quiet-standing RMS values used for ΔRMS% normalization. Supplementary Table S5. Descriptive statistics across the 16 seat-height × task stage conditions. Supplementary Table S6. sEMG model diagnostics, cross-framework robustness, and primary-model stage-specific follow-up tests. Supplementary Table S7. Participant-clustered cumulative-logit GEE results, diagnostics, and sensitivity analyses for REBA and subjective comfort. Supplementary Table S8. CRITIC decision matrix, composite scores, and sensitivity analyses.
