# Supplementary material for: Seat-Height Effects During Seated Shoe Donning in Older Adults: A Multi-Stage Ergonomic Evaluation Integrating Muscle Activity, Postural Exposure, and Subjective Comfort
Source: Sensors (Basel). 2026 Oct 21;26(20):6654. doi: 10.3390/s26206654 (PMC13709135; doi:10.3390/s26206654)
Supplement: Supplementary file 1 [file sensors-26-06654-s001.zip › Supplementary Tables.pdf]

Supplementary Materials

Supplementary Tables S1–S8

Supplementary Table S1. Baseline characteristics of included and excluded participants.

Characteristic	Included ($n = 35$)	Excluded ($n = 7$)	Standardized mean difference
Age (years)	74.60 ± 7.87	76.29 ± 7.16	0.22
Male, n (%)	12 (34.3%)	3 (42.9%)	0.18
Height (m)	1.58 ± 0.07	1.60 ± 0.08	0.22
Body weight (kg)	62.09 ± 9.73	60.71 ± 8.44	0.14
Popliteal height (cm)	41.19 ± 1.76	41.43 ± 1.81	0.13
MBI score	86.13 ± 6.01	88.29 ± 2.06	0.39

Note: Continuous variables are presented as means ± SDs and sex as n (%). Standardized mean differences are provided descriptively to characterize differences between included and excluded participants; no inferential group comparison was performed because of the small excluded sample.

Supplementary Table S2. Preliminary screening of the 12 candidate muscles.

Muscle	Body region	Relative iEMG proportion (%)	Retained for formal experiment
Erector spinae	Trunk	12.55	Yes
Lumbar multifidus	Trunk	9.74	Yes
Flexor carpi ulnaris	Upper limb	8.89	Yes
Latissimus dorsi	Trunk	8.62	No
Medial gastrocnemius	Lower limb	8.58	Yes
Rectus femoris	Lower limb	8.44	Yes
Biceps brachii	Upper limb	7.40	Yes
Vastus lateralis	Lower limb	7.35	No
Anterior deltoid	Upper limb	7.23	No
Vastus medialis	Lower limb	7.14	No
Brachioradialis	Upper limb	7.08	No
Lateral gastrocnemius	Lower limb	6.99	No

Note: Relative iEMG proportion was used as a descriptive screening index. Final muscle selection considered relative iEMG proportion together with task relevance, signal quality, and representative anatomical coverage; therefore, the retained muscles should not be interpreted as simply the six highest-ranked muscles.

Supplementary Table S3. Anatomical landmarks for sEMG electrode placement.

Muscle	Electrode-placement landmark	Electrode orientation	Placement basis / interpretive note
BB	On the line between the medial acromion and the cubital fossa, approximately one-third of the distance from the cubital fossa	Parallel to the longitudinal muscle-fiber direction	Direct SENIAM muscle-specific placement [20].
FCU	Over the palpable muscle belly on the ulnar side of the proximal forearm, along the line from the medial epicondyle toward the pisiform	Parallel to the longitudinal forearm muscle fibers	Anatomical palpation and fiber-direction placement; no directly applicable muscle-specific SENIAM site was specified in the protocol. Potential forearm-muscle crosstalk cannot be excluded.
ES	Approximately two fingerbreadths lateral to the L1 spinous process over the lumbar erector spinae muscle belly	Longitudinal and approximately parallel to the spinal column	Direct SENIAM muscle-specific placement at approximately two fingerbreadths lateral to L1 [20].
LM	Approximately 2–3 cm lateral to the L5 spinous process, over the palpable lumbar multifidus region	Oriented superomedially, approximately parallel to the line from the posterior superior iliac spine toward the upper lumbar spine	SENIAM lumbar-multifidus-region placement [20]; surface signal may include adjacent erector spinae activity and should be interpreted regionally [36,37].
RF	At approximately 50% of the line between the anterior superior iliac spine and the superior border of the patella	Parallel to the longitudinal muscle-fiber direction	Direct SENIAM muscle-specific placement [20].
MG	Over the most prominent bulge of the medial gastrocnemius muscle	Parallel to the longitudinal muscle-fiber direction	Direct SENIAM muscle-specific placement [20].

Note: The BB, the ES, the LM, the RF, and the MG followed the muscle-specific SENIAM locations used in the protocol [20]. The FCU was localized anatomically by palpation and fiber direction rather than a directly applicable muscle-specific SENIAM site. SENIAM-compatible placement does not eliminate limitations in signal selectivity; in particular, the LM channel represents regional lumbar surface activity and may include adjacent erector spinae activity [36,37]. General skin preparation, electrode spacing, side of placement, and signal-quality procedures are described in Section 2.4.2.

Supplementary Table S4. Distribution of quiet-standing RMS values used for ΔRMS% normalization.

Muscle	n	Mean ± SD (μV)	Median (μV)	IQR (μV)	Min–Max (μV)
RF	35	9.27 ± 5.66	7.62	4.99–12.97	2.46–23.34
BB	35	4.91 ± 3.45	3.51	2.70–6.54	2.14–18.11
FCU	35	7.60 ± 4.45	5.52	4.11–12.52	2.21–15.84
ES	35	6.51 ± 4.82	4.65	2.73–9.81	1.89–18.77
MG	35	7.10 ± 3.21	6.53	4.74–8.63	1.98–15.77
LM	35	6.22 ± 3.87	5.69	3.08–7.42	2.15–17.00

Note: Values were calculated from the middle 10 s of the 20 s quiet-standing reference recording and are presented in the amplitude units exported by ErgoLAB. These values were used only as within-participant, within-muscle reference values for ΔRMS% normalization and should not be interpreted as physiological resting equivalence across muscles.

Supplementary Table S5. Descriptive statistics across the 16 seat-height × task stage conditions. Panel A. Muscle-specific natural-log-transformed task RMS (mean ± SD).

Height	Stage	BB log-RMS	FCU log-RMS	ES log-RMS	LM log-RMS	RF log-RMS	MG log-RMS
H1	S1	2.89 ± 0.66	3.16 ± 0.77	3.98 ± 0.82	3.45 ± 0.77	3.41 ± 0.51	3.24 ± 0.56
H1	S2	3.66 ± 0.59	3.86 ± 0.70	2.87 ± 1.04	3.35 ± 0.75	2.45 ± 0.70	3.01 ± 0.90
H1	S3	3.41 ± 0.81	3.44 ± 0.89	2.72 ± 1.09	3.40 ± 0.76	2.77 ± 0.74	2.78 ± 0.80
H1	S4	2.77 ± 0.66	3.19 ± 0.87	4.21 ± 0.65	3.50 ± 0.65	3.44 ± 0.45	3.43 ± 0.39
H2	S1	2.58 ± 0.60	3.08 ± 0.87	3.81 ± 0.68	3.17 ± 0.69	3.25 ± 0.53	3.04 ± 0.54
H2	S2	3.61 ± 0.60	3.88 ± 0.72	3.20 ± 1.01	3.39 ± 0.66	2.62 ± 0.68	3.17 ± 0.91
H2	S3	3.39 ± 0.66	3.53 ± 0.64	3.26 ± 0.83	3.54 ± 0.72	2.91 ± 0.69	2.99 ± 0.75
H2	S4	2.50 ± 0.65	3.07 ± 0.93	4.01 ± 0.69	3.47 ± 0.74	3.38 ± 0.43	3.39 ± 0.46
H3	S1	2.26 ± 0.68	2.65 ± 0.62	3.75 ± 0.68	2.99 ± 0.83	3.00 ± 0.55	2.89 ± 0.56
H3	S2	3.54 ± 0.54	3.86 ± 0.67	3.27 ± 0.96	3.31 ± 0.74	2.81 ± 0.66	2.94 ± 0.71
H3	S3	3.07 ± 0.78	3.20 ± 0.81	2.95 ± 0.90	3.28 ± 0.78	2.98 ± 0.71	3.01 ± 0.70
H3	S4	2.26 ± 0.74	2.70 ± 0.77	3.93 ± 0.65	3.39 ± 0.74	3.24 ± 0.38	3.11 ± 0.43
H4	S1	2.25 ± 0.58	2.78 ± 0.77	3.58 ± 0.69	3.04 ± 0.84	2.97 ± 0.50	2.84 ± 0.49
H4	S2	3.72 ± 0.68	4.06 ± 0.63	3.38 ± 0.95	3.45 ± 0.69	2.82 ± 0.61	3.14 ± 0.85
H4	S3	3.11 ± 0.79	3.55 ± 0.88	3.24 ± 0.87	3.44 ± 0.64	3.04 ± 0.67	3.17 ± 0.73
H4	S4	1.98 ± 0.42	2.39 ± 0.60	3.84 ± 0.63	3.31 ± 0.82	3.25 ± 0.37	3.18 ± 0.40

Panel B. REBA-based postural exposure.

Height	Stage	Median [IQR]	Low risk, n (%)	Medium risk, n (%)	High risk, n (%)	Very high risk, n (%)
H1	S1	6 [6, 7]	0 (0.0%)	31 (88.6%)	4 (11.4%)	0 (0.0%)
H1	S2	8 [8]	1 (2.9%)	7 (20.0%)	27 (77.1%)	0 (0.0%)
H1	S3	6 [6]	0 (0.0%)	35 (100.0%)	0 (0.0%)	0 (0.0%)
H1	S4	6 [4.5, 6]	0 (0.0%)	31 (88.6%)	4 (11.4%)	0 (0.0%)
H2	S1	6 [6, 7]	0 (0.0%)	33 (94.3%)	2 (5.7%)	0 (0.0%)
H2	S2	8 [8]	0 (0.0%)	3 (8.6%)	32 (91.4%)	0 (0.0%)
H2	S3	6 [6]	0 (0.0%)	35 (100.0%)	0 (0.0%)	0 (0.0%)
H2	S4	4 [4, 6]	3 (8.6%)	32 (91.4%)	0 (0.0%)	0 (0.0%)
H3	S1	6 [6, 7]	0 (0.0%)	35 (100.0%)	0 (0.0%)	0 (0.0%)
H3	S2	9 [8, 9]	0 (0.0%)	1 (2.9%)	34 (97.1%)	0 (0.0%)
H3	S3	6 [5.5, 6]	0 (0.0%)	35 (100.0%)	0 (0.0%)	0 (0.0%)
H3	S4	4 [4, 6]	4 (11.4%)	31 (88.6%)	0 (0.0%)	0 (0.0%)
H4	S1	6 [4, 6]	0 (0.0%)	35 (100.0%)	0 (0.0%)	0 (0.0%)
H4	S2	9 [8.5, 9]	0 (0.0%)	0 (0.0%)	35 (100.0%)	0 (0.0%)
H4	S3	5 [4, 6]	1 (2.9%)	34 (97.1%)	0 (0.0%)	0 (0.0%)
H4	S4	4 [4, 5.5]	2 (5.7%)	33 (94.3%)	0 (0.0%)	0 (0.0%)

Panel C. Subjective comfort.

Height	Stage	Median [IQR]
H1	S1	−2.5 [−3, −1]
H1	S2	2 [−1, 2.75]
H1	S3	−1 [−2, 1.75]
H1	S4	−2.5 [−3, −1]
H2	S1	1 [1, 2]
H2	S2	1 [0, 2]
H2	S3	2 [1, 2]
H2	S4	2 [1, 2]
H3	S1	−1 [−1, 1]
H3	S2	2 [2]
H3	S3	1.5 [0.25, 2]
H3	S4	−1 [−1, 1]
H4	S1	1 [−0.75, 2]
H4	S2	−2 [−2, 1]
H4	S3	1 [0, 2]
H4	S4	1 [0.25, 2]

Note: Panel A presents muscle-specific natural-log-transformed task RMS as mean ± SD; each height × stage cell contains 35 participant-level values after averaging the two repeated sEMG trials within participant before log transformation. Secondary ΔRMS% participant-level values are provided in Supplementary Data S1. Panel B presents final condition-level REBA scores as median [IQR] and risk-category frequencies; each cell contains 35 condition-level observations. REBA action/risk categories follow the original REBA framework: negligible = 1, low = 2–3, medium = 4–7, high = 8–10, and very high = 11–15 [6]. No score of 1 occurred in these condition-level data, so a separate negligible-risk column is omitted. Panel C presents subjective-comfort ratings as median [IQR]; both experimental trials were retained without averaging, yielding 70 trial-level ratings per height × stage cell.

Supplementary Table S6. sEMG model diagnostics, cross-framework robustness, and primary-model stage-specific follow-up tests. Panel A1. Diagnostic summary for the secondary ΔRMS%-based mixed-effects models.

Muscle	Residual skewness	Max/min residual variance	|Std. residual| > 3, *n*	Max leave-one-participant shift (SE)	AIC (ML)	BIC (ML)	Converged
BB	2.24	9.09	10	1.18	8435.61	8513.51	Yes
FCU	2.84	18.66	9	1.30	8786.22	8864.12	Yes
ES	3.71	17.81	9	1.67	8615.00	8692.91	Yes
LM	1.63	5.56	10	1.04	7111.75	7189.65	Yes
RF	1.42	6.09	9	0.69	6870.36	6948.26	Yes
MG	2.05	4.92	14	0.90	7246.44	7324.35	Yes

Panel A2. Diagnostic summary for the primary log-RMS mixed-effects models.

Muscle	Residual skewness	Max/min residual variance	|Std. residual| > 3, *n*	Max leave-one-participant shift (SE)	AIC (ML)	BIC (ML)	Converged
BB	0.55	2.78	6	0.81	855.20	933.10	Yes
FCU	0.07	2.79	0	0.53	1160.20	1238.11	Yes
ES	0.18	3.32	2	0.79	1252.41	1330.31	Yes
LM	0.05	2.39	1	0.81	1019.83	1097.73	Yes
RF	0.21	4.13	4	0.75	765.95	843.85	Yes
MG	0.32	3.65	3	0.54	867.19	945.09	Yes

Panel B. Gaussian GEE robustness analysis of natural-log-transformed task RMS without quiet-standing normalization.

Muscle	Height Wald $\chi^2(3)$	Holm *p*	Stage Wald $\chi^2(3)$	Holm *p*	Height × Stage Wald $\chi^2(9)$	Holm *p*
BB	68.38	< 0.001	271.17	< 0.001	79.36	< 0.001
FCU	18.58	0.001	73.94	< 0.001	89.23	< 0.001
ES	6.85	0.154	85.22	< 0.001	141.71	< 0.001
LM	19.94	< 0.001	76.14	< 0.001	33.31	< 0.001
RF	0.48	0.923	70.85	< 0.001	81.49	< 0.001
MG	11.46	0.029	64.97	< 0.001	65.54	< 0.001

Panel C. Cross-framework consistency of seat-height-related sEMG findings.

Muscle	Effect	Secondary ΔRMS% LMM	Primary log-RMS LMM	Gaussian GEE log-RMS	Interpretation
BB	Seat height	Significant	Significant	Significant	Consistent across frameworks
FCU	Seat height	Not significant	Significant	Significant	Analysis-dependent
ES	Seat height	Not significant	Not significant	Not significant	Consistently not supported
LM	Seat height	Not significant	Not significant	Significant	Model-dependent
RF	Seat height	Not significant	Not significant	Not significant	Consistently not supported
MG	Seat height	Significant	Not significant	Significant	Analysis-dependent
BB	Height × Stage	Not significant	Significant	Significant	Analysis-dependent
FCU	Height × Stage	Not significant	Significant	Significant	Analysis-dependent
ES	Height × Stage	Not significant	Significant	Significant	Analysis-dependent
LM	Height × Stage	Not significant	Not significant	Significant	Model-specific
RF	Height × Stage	Significant	Significant	Significant	Consistent across frameworks
MG	Height × Stage	Not significant	Significant	Significant	Analysis-dependent

Panel D. Stage-specific simple effects of seat height from the primary log-RMS mixed-effects model.

Muscle	Stage	$F(3, 510)$	Holm-adjusted p across four stages
BB	S1	14.885	< 0.001
BB	S2	0.928	0.427
BB	S3	5.307	0.003
BB	S4	18.379	< 0.001
FCU	S1	5.247	0.004
FCU	S2	0.802	0.493
FCU	S3	2.261	0.161
FCU	S4	11.808	< 0.001
ES	S1	2.066	0.208
ES	S2	3.756	0.033
ES	S3	5.164	0.006
ES	S4	1.891	0.208
RF	S1	8.321	< 0.001
RF	S2	5.757	0.002
RF	S3	2.594	0.104
RF	S4	1.700	0.166
MG	S1	5.048	0.007
MG	S2	1.824	0.142
MG	S3	3.977	0.024
MG	S4	3.778	0.024

Panel E. Holm-significant pairwise seat-height contrasts within stages with significant primary-model simple effects.

Muscle	Stage	Comparison	Task-RMS ratio (A/B)	95% CI low	95% CI high	Holm-adjusted p
BB	S1	H1 vs H2	1.364	1.095	1.698	0.013
BB	S1	H1 vs H3	1.877	1.507	2.337	< 0.001
BB	S1	H1 vs H4	1.896	1.523	2.362	< 0.001
BB	S1	H2 vs H3	1.376	1.105	1.714	0.013
BB	S1	H2 vs H4	1.391	1.117	1.732	0.013
BB	S3	H1 vs H3	1.407	1.130	1.752	0.014
BB	S3	H1 vs H4	1.357	1.090	1.690	0.026
BB	S3	H2 vs H3	1.379	1.108	1.718	0.021
BB	S3	H2 vs H4	1.331	1.069	1.657	0.032
BB	S4	H1 vs H2	1.316	1.057	1.638	0.043
BB	S4	H1 vs H3	1.680	1.349	2.092	< 0.001
BB	S4	H1 vs H4	2.208	1.773	2.749	< 0.001
BB	S4	H2 vs H3	1.277	1.025	1.590	0.043
BB	S4	H2 vs H4	1.678	1.348	2.090	< 0.001
BB	S4	H3 vs H4	1.314	1.056	1.637	0.043
FCU	S1	H1 vs H3	1.666	1.242	2.235	0.004
FCU	S1	H1 vs H4	1.463	1.091	1.963	0.045
FCU	S1	H2 vs H3	1.537	1.146	2.062	0.021

Panel E. Continued.

Muscle	Stage	Comparison	Task-RMS ratio (A/B)	95% CI low	95% CI high	Holm-adjusted p
FCU	S4	H1 vs H3	1.630	1.215	2.186	0.005
FCU	S4	H1 vs H4	2.222	1.656	2.981	< 0.001
FCU	S4	H2 vs H3	1.446	1.078	1.940	0.042
FCU	S4	H2 vs H4	1.972	1.470	2.646	< 0.001
ES	S2	H1 vs H4	0.596	0.434	0.820	0.009
ES	S3	H1 vs H2	0.579	0.421	0.796	0.005
ES	S3	H1 vs H4	0.592	0.430	0.814	0.007
RF	S1	H1 vs H3	1.505	1.228	1.845	< 0.001
RF	S1	H1 vs H4	1.557	1.270	1.909	< 0.001
RF	S1	H2 vs H3	1.290	1.052	1.581	0.043
RF	S1	H2 vs H4	1.335	1.089	1.636	0.022
RF	S2	H1 vs H3	0.697	0.568	0.854	0.003
RF	S2	H1 vs H4	0.691	0.564	0.847	0.002
MG	S1	H1 vs H3	1.414	1.132	1.765	0.011
MG	S1	H1 vs H4	1.491	1.194	1.862	0.003
MG	S3	H1 vs H4	0.678	0.543	0.847	0.004
MG	S4	H1 vs H3	1.369	1.096	1.709	0.034

Note: Panel A1 summarizes diagnostics for the denominator-dependent ΔRMS%-based mixed-effects models, and Panel A2 reports the corresponding diagnostics for the primary log-RMS mixed-effects models. Residual-variance ratios were calculated across the 16 height × stage cells; leave-one-participant shifts are expressed relative to the full-model SE of the corresponding condition estimate. Panel B reports the log-RMS Gaussian GEE robustness analysis, and Panel C summarizes cross-framework consistency of seat-height-related findings. Panels D–E report stage-specific simple effects and Holm-significant pairwise contrasts from the primary log-RMS mixed-effects model. Holm adjustment was applied as specified in Section 2.6. All primary log-RMS mixed-effects and Gaussian GEE models converged. Task-RMS ratios > 1 indicate higher RMS for Height A than Height B.

Supplementary Table S7. Participant-clustered cumulative-logit GEE results, diagnostics, and sensitivity analyses for REBA and subjective comfort. Panel A. Omnibus tests.

Outcome	Effect	Wald χ^2	df	p
REBA	Seat height	7.34	3	0.062
REBA	Stage	209.95	3	< 0.001
REBA	Height × Stage	121.90	9	< 0.001
Comfort	Seat height	41.78	3	< 0.001
Comfort	Stage	23.05	3	< 0.001
Comfort	Height × Stage	69.49	9	< 0.001
Comfort	Trial	0.60	1	0.437

Panel B1. REBA: simple effects of seat height within stage.

Stage	Wald $\chi^2(3)$	df	Holm-adjusted p
S1	52.85	3	< 0.001
S2	40.29	3	< 0.001
S3	56.86	3	< 0.001
S4	17.79	3	< 0.001

Panel B2. REBA: pairwise seat-height contrasts within stage.

Stage	Comparison	OR (A vs B)	95% CI	Holm-adjusted p
S1	H1–H2	2.324	0.994 to 5.436	0.103
S1	H1–H3	2.172	1.200 to 3.932	0.031
S1	H1–H4	16.587	7.727 to 35.610	< 0.001
S1	H2–H3	0.934	0.435 to 2.007	0.862
S1	H2–H4	7.137	2.881 to 17.678	< 0.001
S1	H3–H4	7.638	3.707 to 15.735	< 0.001
S2	H1–H2	0.271	0.052 to 1.410	0.121
S2	H1–H3	0.016	0.003 to 0.073	< 0.001
S2	H1–H4	0.005	0.001 to 0.035	< 0.001
S2	H2–H3	0.058	0.015 to 0.230	< 0.001
S2	H2–H4	0.019	0.005 to 0.083	< 0.001
S2	H3–H4	0.334	0.126 to 0.884	0.055

Panel B2. Continued.

Stage	Comparison	OR (A vs B)	95% CI	Holm-adjusted p
S3	H1–H2	2.785	1.401 to 5.536	0.010
S3	H1–H3	4.059	2.117 to 7.782	< 0.001
S3	H1–H4	13.862	6.676 to 28.782	< 0.001
S3	H2–H3	1.457	0.761 to 2.791	0.256
S3	H2–H4	4.977	2.164 to 11.449	< 0.001
S3	H3–H4	3.415	1.454 to 8.023	0.010
S4	H1–H2	5.156	1.787 to 14.877	0.010
S4	H1–H3	8.624	3.052 to 24.365	< 0.001
S4	H1–H4	8.178	2.750 to 24.318	< 0.001
S4	H2–H3	1.672	0.750 to 3.727	0.626
S4	H2–H4	1.586	0.563 to 4.468	0.766
S4	H3–H4	0.948	0.446 to 2.015	0.890

Panel C1. Subjective comfort: simple effects of seat height within stage.

Stage	Wald $\chi^2(3)$	df	Holm-adjusted p
S1	33.17	3	< 0.001
S2	41.61	3	< 0.001
S3	25.75	3	< 0.001
S4	37.37	3	< 0.001

Panel C2. Subjective comfort: pairwise seat-height contrasts within stage.

Stage	Comparison	OR (A vs B)	95% CI	Holm-adjusted p
S1	H1–H2	0.047	0.016 to 0.137	< 0.001
S1	H1–H3	0.215	0.099 to 0.468	< 0.001
S1	H1–H4	0.081	0.025 to 0.262	< 0.001
S1	H2–H3	4.597	2.500 to 8.453	< 0.001
S1	H2–H4	1.730	0.820 to 3.650	0.150
S1	H3–H4	0.376	0.161 to 0.877	0.047
S2	H1–H2	1.279	0.549 to 2.982	0.568
S2	H1–H3	0.431	0.183 to 1.017	0.109
S2	H1–H4	8.691	3.363 to 22.464	< 0.001
S2	H2–H3	0.337	0.187 to 0.609	< 0.001
S2	H2–H4	6.793	3.326 to 13.874	< 0.001
S2	H3–H4	20.143	7.991 to 50.771	< 0.001

Panel C2. Continued.

Stage	Comparison	OR (A vs B)	95% CI	Holm-adjusted p
S3	H1–H2	0.132	0.057 to 0.308	< 0.001
S3	H1–H3	0.184	0.093 to 0.367	< 0.001
S3	H1–H4	0.204	0.083 to 0.503	0.002
S3	H2–H3	1.393	0.803 to 2.416	0.714
S3	H2–H4	1.546	0.736 to 3.247	0.714
S3	H3–H4	1.109	0.534 to 2.304	0.781
S4	H1–H2	0.027	0.008 to 0.089	< 0.001
S4	H1–H3	0.139	0.057 to 0.343	< 0.001
S4	H1–H4	0.045	0.014 to 0.142	< 0.001
S4	H2–H3	5.231	2.558 to 10.697	< 0.001
S4	H2–H4	1.692	0.758 to 3.779	0.200
S4	H3–H4	0.323	0.143 to 0.733	0.014

Panel D. Cumulative-logit GEE diagnostics and proportional-odds assessment.

Outcome	Converged	Working correlation	Empty condition × category cells	Cells with $n < 5$	Proportional-odds diagnostic
REBA	Yes	0.014	66/128	96/128	Not firmly supported; sparse thresholds produced quasi-separation/unstable covariance in several cumulative binary GEE fits.
Comfort	Yes	0.100	15/112	44/112	All categories represented overall; threshold heterogeneity/sparsity prompted alternative-specification sensitivity analyses.

Panel E. Alternative outcome-specification sensitivity analyses.

Outcome/model	Effect	Wald χ^2	df	p
REBA Gaussian GEE	Seat height	26.37	3	< 0.001
REBA Gaussian GEE	Stage	1364.84	3	< 0.001
REBA Gaussian GEE	Height × Stage	168.82	9	< 0.001
Comfort Gaussian GEE	Seat height	82.73	3	< 0.001
Comfort Gaussian GEE	Stage	26.93	3	< 0.001
Comfort Gaussian GEE	Height × Stage	159.72	9	< 0.001
Comfort Gaussian GEE	Trial	0.59	1	0.442
Comfort 3-category ordinal GEE	Seat height	42.89	3	< 0.001
Comfort 3-category ordinal GEE	Stage	12.12	3	0.007
Comfort 3-category ordinal GEE	Height × Stage	63.06	9	< 0.001
Comfort 3-category ordinal GEE	Trial	2.49	1	0.115

Note: The primary REBA and subjective-comfort analyses used a participant-clustered cumulative-logit GEE with exchangeable working correlation and robust standard errors, and both primary models converged. Panel D reports category sparsity and proportional-odds diagnostics. Because the proportional-odds assumption could not be regarded as firmly established for sparse REBA scores, Panel E reports Gaussian GEE sensitivity results; comfort robustness was assessed with a Gaussian GEE and a collapsed three-category ordinal GEE. For the primary ordinal models, OR > 1 indicates greater odds of a higher ordinal outcome category for Height A relative to Height B. REBA stage-specific simple-effects p-values were Holm-adjusted across the four stages, and pairwise p-values were Holm-adjusted across the six seat-height comparisons within each stage.

Supplementary Table S8. CRITIC decision matrix, composite scores, and sensitivity analyses. Panel A. Primary 16-condition decision matrix using the within-muscle-standardized log-RMS sEMG summary.

Height	Stage	Standardized log-RMS sEMG summary	REBA mean	Comfort mean	CRITIC score
H1	S1	0.1542	6.57	−1.60	0.3322
H1	S2	−0.5939	7.49	1.03	0.6448
H1	S3	−0.8225	6.20	−0.44	0.6587
H1	S4	0.4265	5.74	−1.86	0.3288
H2	S1	−0.3452	6.20	1.19	0.7258
H2	S2	−0.0683	7.86	0.81	0.4934
H2	S3	−0.0739	5.77	1.40	0.7330
H2	S4	0.3062	4.89	1.34	0.7330
H3	S1	−0.4583	6.23	−0.23	0.6084
H3	S2	0.3562	8.49	1.66	0.4400
H3	S3	0.0074	5.60	1.14	0.7079
H3	S4	0.2751	4.60	−0.16	0.6200
H4	S1	−0.5110	5.31	0.71	0.7891
H4	S2	0.7855	8.74	−1.01	0.0810
H4	S3	0.5261	4.94	1.06	0.6600
H4	S4	0.0359	4.63	0.91	0.7652

Panel B. Direction-normalized 16-condition decision matrix used for CRITIC weighting.

Height	Stage	Normalized standardized sEMG summary	Normalized REBA	Normalized subjective comfort
H1	S1	0.3926	0.5242	0.0732
H1	S2	0.8578	0.3019	0.8211
H1	S3	1.0000	0.6135	0.4024
H1	S4	0.2233	0.7246	0.0000
H2	S1	0.7032	0.6135	0.8659
H2	S2	0.5310	0.2126	0.7602
H2	S3	0.5344	0.7174	0.9268
H2	S4	0.2981	0.9300	0.9106
H3	S1	0.7735	0.6063	0.4634
H3	S2	0.2670	0.0604	1.0000
H3	S3	0.4839	0.7585	0.8537
H3	S4	0.3174	1.0000	0.4837
H4	S1	0.8063	0.8285	0.7317
H4	S2	0.0000	0.0000	0.2398
H4	S3	0.1613	0.9179	0.8293
H4	S4	0.4662	0.9928	0.7886

Panel C. CRITIC correlation, conflict, information-content, and weight calculations.

Indicator	SD (σ_j)	r with muscle	r with REBA	r with comfort	$\sum(1 - r_{jk})$	Information C_j	Weight
Standardized sEMG summary	0.2794	1.0000	0.0787	0.1811	1.7402	0.4861	0.3012
REBA	0.3188	0.0787	1.0000	0.0941	1.8272	0.5825	0.3610
Subjective comfort	0.3160	0.1811	0.0941	1.0000	1.7248	0.5451	0.3378

Panel D. Overall CRITIC scores and ranks.

Height	Overall CRITIC score	Overall rank
H1	0.4911	4
H2	0.6713	1
H3	0.5941	2
H4	0.5738	3

Panel E. Sensitivity-analysis rankings.

Sensitivity scenario	Overall ranking
Original ΔRMS%-based component	H2 > H3 > H4 > H1
Log-RMS min–max-standardized component	H2 > H3 > H4 > H1
Rank-based sEMG component	H2 > H3 > H4 > H1
S1 emphasized (0.40; others 0.20)	H2 > H4 > H3 > H1
S2 emphasized (0.40; others 0.20)	H2 > H3 > H1 > H4
S3 emphasized (0.40; others 0.20)	H2 > H3 > H4 > H1
S4 emphasized (0.40; others 0.20)	H2 > H4 > H3 > H1
Omit S1	H2 > H3 > H1 > H4
Omit S2	H4 > H2 > H3 > H1
Omit S3	H2 > H3 > H4 > H1
Omit S4	H2 > H3 > H1 > H4
sEMG component excluded	H2 > H4 > H3 > H1
sEMG orientation reversed	H2 > H4 > H3 > H1

Note: CRITIC was used as an exploratory mathematical integration and not as an inferential or physiological model. The primary muscle component was a within-muscle-standardized log-RMS sEMG summary. Lower values of this summary and REBA were coded in the cost direction solely to align mathematical score orientation; this did not imply that lower muscle activation was inherently physiologically preferable. Panels A–C document the log-RMS-based CRITIC calculation, Panel D reports the overall scores and ranks, and Panel E compares alternative sEMG scaling, indicator-direction/exclusion, and stage-weighting/omission scenarios. Persistence of an ordering across sensitivity analyses indicates numerical robustness only and should not be interpreted as physiological validity or ergonomic superiority.
