# Supplementary material for: Seat-Height Effects During Seated Shoe Donning in Older Adults: A Multi-Stage Ergonomic Evaluation Integrating Muscle Activity, Postural Exposure, and Subjective Comfort
Source: Sensors (Basel). 2026 Oct 21;26(20):6654. doi: 10.3390/s26206654 (PMC13709135; doi:10.3390/s26206654)
Supplement: Supplementary file 1 [file sensors-26-06654-s001.zip › Supplementary_Data_S1_Deidentified_Supporting_Data.pdf]

Supplementary Data S1

De-identified Supporting Data for the Primary Outcomes

Article: Seat-Height Effects During Seated Shoe Donning in Older Adults: A Multi-Stage Ergonomic Evaluation Integrating Muscle Activity, Postural Exposure, and Subjective Comfort

Manuscript ID: sensors-4525074

Purpose of this PDF. This document is a human-readable LaTeX/PDF rendering of the accompanying Excel workbook `Supplementary_Data_S1_Deidentified_Supporting_Data.xlsx`. The values below are reproduced directly from the workbook without analytical transformation. The Excel workbook remains the machine-readable primary supplementary-data file.

Workbook SHA-256: `e2e5e0b02a07f33688da63825993d33bac1230c548a3787aadb20bafe92b0bb9`

Workbook contents: README (10 entries); sEMG_Primary (3360 data records); REBA (16 condition-level summary records); Comfort (1120 trial-level records); Data_Dictionary (23 variable definitions).

README

Supplementary Data S1: De-identified supporting data for the primary outcomes

Dataset scope: This workbook contains de-identified participant-level sEMG and subjective-comfort data supporting the primary outcomes, together with the manuscript-authoritative condition-level REBA summary. It is not a complete raw or preprocessing dataset and does not include raw sEMG signals, video recordings, intermediate cleaning variables, original rater-level REBA records, or every sensitivity-analysis input.

Participants: 35 older adults included in the final analysis.

Seat-height coding: H1 = 60%, H2 = 80%, H3 = 100%, and H4 = 120% of each participant's popliteal height.

Task-stage coding: S1 = sitting down; S2 = forward bending and shoe donning; S3 = seated trunk re-extension; S4 = standing up.

sEMG outcome: Delta_RMS_pct contains the as-analyzed stage-level ΔRMS% values for BB, FCU, ES, LM, RF, and MG. The manuscript protocol defines ΔRMS% relative to a muscle-specific quiet-standing reference derived from the middle 10 s of a 20-s quiet-standing recording.

REBA outcome: The REBA sheet reports the manuscript-authoritative summary for the 16 Seat Height × Stage conditions (n = 35 per condition), matching Supplementary Table S5 Panel B and the REBA means used in Supplementary Table S8. Exact participant-level REBA scores are not reconstructed from summary statistics in this public supporting file.

Comfort outcome: Comfort_Rating retains both trial-level ratings for each Participant × Seat Height × Stage condition (n = 1120). Ratings range from -3 to +3.

De-identification: Participant identifiers are study codes only. Direct personal identifiers are not included.

Recommended dataset name: De-identified supporting dataset for the primary outcomes.

sEMG_Primary

Participant_ID	Seat_Height	Seat_Height_pct_PH	Stage	Muscle	Delta_RMS_pct
1	H1	60	S1	RF	295.739910313901
1	H1	60	S2	RF	-39.1816143497758
1	H1	60	S3	RF	-72.1973094170404
1	H1	60	S4	RF	477.186098654708
1	H1	60	S1	BB	109.586776859504
1	H1	60	S2	BB	840.330578512397
1	H1	60	S3	BB	256.363636363636
1	H1	60	S4	BB	111.570247933884
1	H1	60	S1	FCU	-15.4850746268657
1	H1	60	S2	FCU	694.029850746269
1	H1	60	S3	FCU	229.850746268657
1	H1	60	S4	FCU	4.47761194029849
1	H1	60	S1	ES	-62.8803245436105
1	H1	60	S2	ES	-30.3752535496957
1	H1	60	S3	ES	-39.2494929006085
1	H1	60	S4	ES	22.2616632860041
1	H1	60	S1	MG	-55.704270235819
1	H1	60	S2	MG	6.62842574888463
1	H1	60	S3	MG	5.84236243892076
1	H1	60	S4	MG	-41.2789462502656
1	H1	60	S1	LM	1.44454799627213
1	H1	60	S2	LM	-11.8825722273998
1	H1	60	S3	LM	245.433364398882
1	H1	60	S4	LM	48.0428704566636
1	H2	80	S1	RF	421.915103652517
1	H2	80	S2	RF	22.4086870681145
1	H2	80	S3	RF	-22.2112537018756
1	H2	80	S4	RF	613.030602171767
1	H2	80	S1	BB	177.13717693837
1	H2	80	S2	BB	634.791252485089
1	H2	80	S3	BB	262.823061630219
1	H2	80	S4	BB	142.544731610338
1	H2	80	S1	FCU	222.033898305085
1	H2	80	S2	FCU	1061.86440677966
1	H2	80	S3	FCU	581.638418079096
1	H2	80	S4	FCU	112.146892655367
1	H2	80	S1	ES	177.13717693837
1	H2	80	S2	ES	634.791252485089
1	H2	80	S3	ES	262.823061630219
1	H2	80	S4	ES	142.544731610338
1	H2	80	S1	MG	-5.68181818181817
1	H2	80	S2	MG	14.7727272727273
1	H2	80	S3	MG	4.56864564007423
1	H2	80	S4	MG	76.8552875695733
1	H2	80	S1	LM	-25.6428571428571
1	H2	80	S2	LM	-18.75
1	H2	80	S3	LM	60.5714285714286
1	H2	80	S4	LM	16.3928571428572
1	H3	100	S1	RF	320.818505338078
1	H3	100	S2	RF	1.15658362989323
1	H3	100	S3	RF	-35.9430604982206

Continued on next page

Participant_ID	Seat_Height	Seat_Height_pct_PH	Stage	Muscle	Delta_RMS_pct
1	H3	100	S4	RF	527.491103202847
1	H3	100	S1	BB	55.0724637681159
1	H3	100	S2	BB	529.710144927536
1	H3	100	S3	BB	144.782608695652
1	H3	100	S4	BB	-13.6231884057971
1	H3	100	S1	FCU	135.87786259542
1	H3	100	S2	FCU	1059.03307888041
1	H3	100	S3	FCU	552.162849872774
1	H3	100	S4	FCU	28.4987277353689
1	H3	100	S1	ES	165.714285714286
1	H3	100	S2	ES	598.666666666667
1	H3	100	S3	ES	117.428571428571
1	H3	100	S4	ES	225.428571428571
1	H3	100	S1	MG	-34.4241922120961
1	H3	100	S2	MG	107.580778790389
1	H3	100	S3	MG	62.3446561723281
1	H3	100	S4	MG	35.5840927920464
1	H3	100	S1	LM	83.572710951526
1	H3	100	S2	LM	68.6714542190305
1	H3	100	S3	LM	25.7630161579892
1	H3	100	S4	LM	155.834829443447
1	H4	120	S1	RF	94.2629482071713
1	H4	120	S2	RF	58.9641434262948
1	H4	120	S3	RF	81.9123505976095
1	H4	120	S4	RF	205.019920318725
1	H4	120	S1	BB	317.07492795389
1	H4	120	S2	BB	585.086455331412
1	H4	120	S3	BB	219.524495677233
1	H4	120	S4	BB	-19.0922190201729
1	H4	120	S1	FCU	100.250626566416
1	H4	120	S2	FCU	1254.88721804511
1	H4	120	S3	FCU	903.508771929824
1	H4	120	S4	FCU	17.0426065162907
1	H4	120	S1	ES	104.490500863558
1	H4	120	S2	ES	381.088082901554
1	H4	120	S3	ES	178.065630397237
1	H4	120	S4	ES	248.100172711572
1	H4	120	S1	MG	-5.46906187624751
1	H4	120	S2	MG	122.395209580838
1	H4	120	S3	MG	28.5429141716567
1	H4	120	S4	MG	60.9181636726547
1	H4	120	S1	LM	56.5928777670837
1	H4	120	S2	LM	49.7593840230991
1	H4	120	S3	LM	143.599615014437
1	H4	120	S4	LM	170.452358036574
2	H1	60	S1	RF	73.9906103286385
2	H1	60	S2	RF	106.948356807512
2	H1	60	S3	RF	77.1830985915493
2	H1	60	S4	RF	68.169014084507
2	H1	60	S1	BB	164.459930313589
2	H1	60	S2	BB	1025.43554006969
2	H1	60	S3	BB	557.142857142857
2	H1	60	S4	BB	201.742160278746
2	H1	60	S1	FCU	-45.9242250287027
2	H1	60	S2	FCU	420.89552238806
2	H1	60	S3	FCU	43.2835820895522

Continued on next page

Participant_ID	Seat_Height	Seat_Height_pct_PH	Stage	Muscle	Delta_RMS_pct
2	H1	60	S4	FCU	54.7646383467279
2	H1	60	S1	ES	31.844316674038
2	H1	60	S2	ES	472.224679345422
2	H1	60	S3	ES	-2.12295444493587
2	H1	60	S4	ES	248.695267580716
2	H1	60	S1	MG	76.1214630779848
2	H1	60	S2	MG	319.668737060041
2	H1	60	S3	MG	92.8916494133885
2	H1	60	S4	MG	206.901311249137
2	H1	60	S1	LM	194.982394366197
2	H1	60	S2	LM	35.9154929577465
2	H1	60	S3	LM	235.739436619718
2	H1	60	S4	LM	174.911971830986
2	H2	80	S1	RF	50.7377049180328
2	H2	80	S2	RF	25.7377049180328
2	H2	80	S3	RF	142.622950819672
2	H2	80	S4	RF	33.1967213114754
2	H2	80	S1	BB	58.5365853658536
2	H2	80	S2	BB	588.15331010453
2	H2	80	S3	BB	702.787456445993
2	H2	80	S4	BB	33.1010452961672
2	H2	80	S1	FCU	76.9230769230769
2	H2	80	S2	FCU	896.394230769231
2	H2	80	S3	FCU	757.692307692308
2	H2	80	S4	FCU	-3.36538461538463
2	H2	80	S1	ES	-43.0400798270414
2	H2	80	S2	ES	-22.734076168302
2	H2	80	S3	ES	-74.322301679694
2	H2	80	S4	ES	-49.2599368035922
2	H2	80	S1	MG	462.061855670103
2	H2	80	S2	MG	1014.32989690722
2	H2	80	S3	MG	195.051546391753
2	H2	80	S4	MG	177.938144329897
2	H2	80	S1	LM	-31.8620689655172
2	H2	80	S2	LM	21.8620689655172
2	H2	80	S3	LM	28.7586206896552
2	H2	80	S4	LM	-31.5172413793103
2	H3	100	S1	RF	74.7021081576535
2	H3	100	S2	RF	73.8771769019248
2	H3	100	S3	RF	83.3180568285976
2	H3	100	S4	RF	51.7873510540788
2	H3	100	S1	BB	45.3488372093023
2	H3	100	S2	BB	500.290697674418
2	H3	100	S3	BB	476.162790697674
2	H3	100	S4	BB	2.32558139534884
2	H3	100	S1	FCU	106.344410876133
2	H3	100	S2	FCU	1777.03927492447
2	H3	100	S3	FCU	841.691842900302
2	H3	100	S4	FCU	425.075528700906
2	H3	100	S1	ES	-7.73168103448275
2	H3	100	S2	ES	427.289870689655
2	H3	100	S3	ES	-58.9978448275862
2	H3	100	S4	ES	-17.4568965517241
2	H3	100	S1	MG	385.114885114885
2	H3	100	S2	MG	760.03996003996
2	H3	100	S3	MG	194.405594405594

Continued on next page

Participant_ID	Seat_Height	Seat_Height_pct_PH	Stage	Muscle	Delta_RMS_pct
2	H3	100	S4	MG	116.883116883117
2	H3	100	S1	LM	-15.7575757575758
2	H3	100	S2	LM	-8.38383838383838
2	H3	100	S3	LM	19.1919191919192
2	H3	100	S4	LM	14.4444444444444
2	H4	120	S1	RF	64.5537948290242
2	H4	120	S2	RF	46.7055879899917
2	H4	120	S3	RF	528.106755629691
2	H4	120	S4	RF	111.175979983319
2	H4	120	S1	BB	150
2	H4	120	S2	BB	565.350877192983
2	H4	120	S3	BB	1183.77192982456
2	H4	120	S4	BB	132.017543859649
2	H4	120	S1	FCU	771.698113207547
2	H4	120	S2	FCU	3111.32075471698
2	H4	120	S3	FCU	1529.43396226415
2	H4	120	S4	FCU	258.867924528302
2	H4	120	S1	ES	113.984710050345
2	H4	120	S2	ES	116.48331157934
2	H4	120	S3	ES	40.4251351855305
2	H4	120	S4	ES	340.275964944994
2	H4	120	S1	MG	110
2	H4	120	S2	MG	908.518518518519
2	H4	120	S3	MG	480
2	H4	120	S4	MG	215.925925925926
2	H4	120	S1	LM	76.2886597938144
2	H4	120	S2	LM	180.412371134021
2	H4	120	S3	LM	204.712812960236
2	H4	120	S4	LM	40.500736377025
3	H1	60	S1	RF	228.262046561992
3	H1	60	S2	RF	162.154845695723
3	H1	60	S3	RF	167.785598267461
3	H1	60	S4	RF	194.748240389821
3	H1	60	S1	BB	448.636698599853
3	H1	60	S2	BB	693.146647015475
3	H1	60	S3	BB	273.986735445836
3	H1	60	S4	BB	333.677229182019
3	H1	60	S1	FCU	923.68
3	H1	60	S2	FCU	1104.48
3	H1	60	S3	FCU	1384
3	H1	60	S4	FCU	533.28
3	H1	60	S1	ES	1127.39726027397
3	H1	60	S2	ES	-12.7316680096696
3	H1	60	S3	ES	101.531023368251
3	H1	60	S4	ES	1176.63174858985
3	H1	60	S1	MG	179.030837004405
3	H1	60	S2	MG	38.7665198237886
3	H1	60	S3	MG	-17.7973568281938
3	H1	60	S4	MG	143.52422907489
3	H1	60	S1	LM	8.1019077517508
3	H1	60	S2	LM	-63.4629316590196
3	H1	60	S3	LM	7.00313933832409
3	H1	60	S4	LM	-28.4472349673992
3	H2	80	S1	RF	-43.524207421971
3	H2	80	S2	RF	57.1639223396412
3	H2	80	S3	RF	-22.2167608749078

Continued on next page

Participant_ID	Seat_Height	Seat_Height_pct_PH	Stage	Muscle	Delta_RMS_pct
3	H2	80	S4	RF	-47.9970508724502
3	H2	80	S1	BB	136.726546906188
3	H2	80	S2	BB	283.13373253493
3	H2	80	S3	BB	156.88622754491
3	H2	80	S4	BB	190.219560878243
3	H2	80	S1	FCU	54.9881235154394
3	H2	80	S2	FCU	1492.75534441805
3	H2	80	S3	FCU	394.893111638955
3	H2	80	S4	FCU	58.7885985748218
3	H2	80	S1	ES	740.452755905512
3	H2	80	S2	ES	25.7874015748031
3	H2	80	S3	ES	-2.80511811023622
3	H2	80	S4	ES	733.366141732283
3	H2	80	S1	MG	130.400696864111
3	H2	80	S2	MG	27.7003484320557
3	H2	80	S3	MG	12.3693379790941
3	H2	80	S4	MG	238.850174216028
3	H2	80	S1	LM	-46.3930701505254
3	H2	80	S2	LM	-67.0264129508662
3	H2	80	S3	LM	-27.6341948310139
3	H2	80	S4	LM	-68.2760579380858
3	H3	100	S1	RF	304.490322580645
3	H3	100	S2	RF	433.161290322581
3	H3	100	S3	RF	52.541935483871
3	H3	100	S4	RF	33.8064516129032
3	H3	100	S1	BB	117.142857142857
3	H3	100	S2	BB	837.428571428572
3	H3	100	S3	BB	693.428571428571
3	H3	100	S4	BB	245.428571428571
3	H3	100	S1	FCU	1000.91463414634
3	H3	100	S2	FCU	3040.85365853659
3	H3	100	S3	FCU	999.390243902439
3	H3	100	S4	FCU	1327.43902439024
3	H3	100	S1	ES	645.24236983842
3	H3	100	S2	ES	72.591262716936
3	H3	100	S3	ES	85.9365649311789
3	H3	100	S4	ES	476.72052663076
3	H3	100	S1	MG	86.3060016906171
3	H3	100	S2	MG	4.81825866441251
3	H3	100	S3	MG	11.4116652578191
3	H3	100	S4	MG	109.38292476754
3	H3	100	S1	LM	39.5892351274788
3	H3	100	S2	LM	25.3541076487252
3	H3	100	S3	LM	623.796033994334
3	H3	100	S4	LM	134.13597733711
3	H4	120	S1	RF	61.4829744351331
3	H4	120	S2	RF	-21.2793041264453
3	H4	120	S3	RF	-40.4158268802376
3	H4	120	S4	RF	-56.2745306035854
3	H4	120	S1	BB	352.571428571429
3	H4	120	S2	BB	1007.71428571429
3	H4	120	S3	BB	931.142857142857
3	H4	120	S4	BB	130
3	H4	120	S1	FCU	341.714285714286
3	H4	120	S2	FCU	2516.28571428571
3	H4	120	S3	FCU	1801.42857142857

Continued on next page

Participant_ID	Seat_Height	Seat_Height_pct_PH	Stage	Muscle	Delta_RMS_pct
3	H4	120	S4	FCU	67.4285714285714
3	H4	120	S1	ES	169.428284854564
3	H4	120	S2	ES	8.72617853560682
3	H4	120	S3	ES	13.9618856569709
3	H4	120	S4	ES	83.4503510531595
3	H4	120	S1	MG	150.883095037847
3	H4	120	S2	MG	22.3717409587889
3	H4	120	S3	MG	60.7232968881413
3	H4	120	S4	MG	126.661059714045
3	H4	120	S1	LM	478.021978021978
3	H4	120	S2	LM	51.7948717948718
3	H4	120	S3	LM	333.113553113553
3	H4	120	S4	LM	84.1758241758242
4	H1	60	S1	RF	175.768321513002
4	H1	60	S2	RF	-10.4018912529551
4	H1	60	S3	RF	26.7139479905437
4	H1	60	S4	RF	235.224586288416
4	H1	60	S1	BB	162.861736334405
4	H1	60	S2	BB	299.83922829582
4	H1	60	S3	BB	375.72347266881
4	H1	60	S4	BB	274.75884244373
4	H1	60	S1	FCU	173.2
4	H1	60	S2	FCU	609.4
4	H1	60	S3	FCU	737.2
4	H1	60	S4	FCU	204.2
4	H1	60	S1	ES	323.356807511737
4	H1	60	S2	ES	-57.3552425665102
4	H1	60	S3	ES	-24.6478873239436
4	H1	60	S4	ES	899.374021909233
4	H1	60	S1	MG	236.200716845878
4	H1	60	S2	MG	123.118279569892
4	H1	60	S3	MG	107.52688172043
4	H1	60	S4	MG	675.985663082437
4	H1	60	S1	LM	-70.1320132013201
4	H1	60	S2	LM	-49.9009900990099
4	H1	60	S3	LM	-69.4389438943894
4	H1	60	S4	LM	-27.7227722772277
4	H2	80	S1	RF	53.9336492890995
4	H2	80	S2	RF	34.1232227488152
4	H2	80	S3	RF	74.0284360189573
4	H2	80	S4	RF	124.739336492891
4	H2	80	S1	BB	279.752704791345
4	H2	80	S2	BB	527.511591962906
4	H2	80	S3	BB	358.732612055641
4	H2	80	S4	BB	360.278207109737
4	H2	80	S1	FCU	496.651785714286
4	H2	80	S2	FCU	981.25
4	H2	80	S3	FCU	277.901785714286
4	H2	80	S4	FCU	695.982142857143
4	H2	80	S1	ES	158.66077656359
4	H2	80	S2	ES	-9.88142292490119
4	H2	80	S3	ES	38.9444315275517
4	H2	80	S4	ES	124.552429667519
4	H2	80	S1	MG	143.873085339169
4	H2	80	S2	MG	42.3413566739606
4	H2	80	S3	MG	84.9015317286652

Continued on next page

Participant_ID	Seat_Height	Seat_Height_pct_PH	Stage	Muscle	Delta_RMS_pct
4	H2	80	S4	MG	235.229759299781
4	H2	80	S1	LM	-24.8027613412229
4	H2	80	S2	LM	18.2938856015779
4	H2	80	S3	LM	-10.4536489151874
4	H2	80	S4	LM	-10.4536489151874
4	H3	100	S1	RF	70.1164294954722
4	H3	100	S2	RF	10.8667529107374
4	H3	100	S3	RF	122.121604139715
4	H3	100	S4	RF	132.729624838292
4	H3	100	S1	BB	259.467455621302
4	H3	100	S2	BB	586.094674556213
4	H3	100	S3	BB	582.840236686391
4	H3	100	S4	BB	165.976331360947
4	H3	100	S1	FCU	85.7385398981324
4	H3	100	S2	FCU	675.042444821732
4	H3	100	S3	FCU	278.098471986418
4	H3	100	S4	FCU	243.123938879457
4	H3	100	S1	ES	418.901890189019
4	H3	100	S2	ES	8.64086408640865
4	H3	100	S3	ES	395.049504950495
4	H3	100	S4	ES	683.438343834384
4	H3	100	S1	MG	179.84962406015
4	H3	100	S2	MG	38.9473684210526
4	H3	100	S3	MG	161.804511278195
4	H3	100	S4	MG	322.105263157895
4	H3	100	S1	LM	-37.2749099639856
4	H3	100	S2	LM	-17.5270108043217
4	H3	100	S3	LM	-37.875150060024
4	H3	100	S4	LM	-22.7490996398559
4	H4	120	S1	RF	69.5928753180661
4	H4	120	S2	RF	77.6081424936387
4	H4	120	S3	RF	106.870229007634
4	H4	120	S4	RF	112.595419847328
4	H4	120	S1	BB	92.3295454545454
4	H4	120	S2	BB	698.295454545455
4	H4	120	S3	BB	382.386363636364
4	H4	120	S4	BB	178.125
4	H4	120	S1	FCU	287.571701720841
4	H4	120	S2	FCU	895.219885277246
4	H4	120	S3	FCU	1760.61185468451
4	H4	120	S4	FCU	40.3441682600382
4	H4	120	S1	ES	111.967545638945
4	H4	120	S2	ES	155.882352941176
4	H4	120	S3	ES	-22.7180527383367
4	H4	120	S4	ES	295.233265720081
4	H4	120	S1	MG	196.018376722818
4	H4	120	S2	MG	69.5252679938744
4	H4	120	S3	MG	219.295558958652
4	H4	120	S4	MG	322.205206738132
4	H4	120	S1	LM	-6.19158878504674
4	H4	120	S2	LM	66.588785046729
4	H4	120	S3	LM	-16.0046728971963
4	H4	120	S4	LM	38.9018691588785
5	H1	60	S1	RF	69.4922031187525
5	H1	60	S2	RF	24.9900039984006
5	H1	60	S3	RF	33.0267892842863

Continued on next page

Participant_ID	Seat_Height	Seat_Height_pct_PH	Stage	Muscle	Delta_RMS_pct
5	H1	60	S4	RF	100.679728108757
5	H1	60	S1	BB	475.462962962963
5	H1	60	S2	BB	786.111111111111
5	H1	60	S3	BB	963.888888888889
5	H1	60	S4	BB	488.888888888889
5	H1	60	S1	FCU	811.897106109325
5	H1	60	S2	FCU	1649.51768488746
5	H1	60	S3	FCU	122.186495176849
5	H1	60	S4	FCU	69.7749196141479
5	H1	60	S1	ES	243.051616562677
5	H1	60	S2	ES	-70.1077708451503
5	H1	60	S3	ES	-86.3301191151446
5	H1	60	S4	ES	296.5399886557
5	H1	60	S1	MG	114.10373066424
5	H1	60	S2	MG	80.0727934485896
5	H1	60	S3	MG	60.0545950864422
5	H1	60	S4	MG	328.571428571429
5	H1	60	S1	LM	12.3428571428572
5	H1	60	S2	LM	603.771428571428
5	H1	60	S3	LM	-52.6857142857143
5	H1	60	S4	LM	159.542857142857
5	H2	80	S1	RF	52.2688356164384
5	H2	80	S2	RF	54.8801369863014
5	H2	80	S3	RF	25.6849315068493
5	H2	80	S4	RF	98.2448630136986
5	H2	80	S1	BB	1412.5
5	H2	80	S2	BB	617.424242424242
5	H2	80	S3	BB	823.106060606061
5	H2	80	S4	BB	175.378787878788
5	H2	80	S1	FCU	1434.13793103448
5	H2	80	S2	FCU	421.034482758621
5	H2	80	S3	FCU	1680.34482758621
5	H2	80	S4	FCU	209.655172413793
5	H2	80	S1	ES	491.308691308691
5	H2	80	S2	ES	-52.5474525474525
5	H2	80	S3	ES	102.097902097902
5	H2	80	S4	ES	758.041958041958
5	H2	80	S1	MG	77.9498525073746
5	H2	80	S2	MG	74.1150442477876
5	H2	80	S3	MG	53.6135693215339
5	H2	80	S4	MG	122.713864306785
5	H2	80	S1	LM	112.805755395683
5	H2	80	S2	LM	834.820143884892
5	H2	80	S3	LM	660.143884892086
5	H2	80	S4	LM	335.68345323741
5	H3	100	S1	RF	81.2790697674419
5	H3	100	S2	RF	81.9186046511628
5	H3	100	S3	RF	158.779069767442
5	H3	100	S4	RF	58.1395348837209
5	H3	100	S1	BB	50.9433962264151
5	H3	100	S2	BB	598.820754716981
5	H3	100	S3	BB	354.481132075472
5	H3	100	S4	BB	66.7452830188679
5	H3	100	S1	FCU	35.6340288924558
5	H3	100	S2	FCU	873.033707865168
5	H3	100	S3	FCU	24.5585874799358

Continued on next page

Participant_ID	Seat_Height	Seat_Height_pct_PH	Stage	Muscle	Delta_RMS_pct
5	H3	100	S4	FCU	-38.6837881219904
5	H3	100	S1	ES	125.716145833333
5	H3	100	S2	ES	-73.2421875
5	H3	100	S3	ES	-80.9895833333333
5	H3	100	S4	ES	149.934895833333
5	H3	100	S1	MG	80.9726962457338
5	H3	100	S2	MG	66.9795221843003
5	H3	100	S3	MG	118.344709897611
5	H3	100	S4	MG	33.7883959044368
5	H3	100	S1	LM	74.3515850144092
5	H3	100	S2	LM	373.390970220941
5	H3	100	S3	LM	110.951008645533
5	H3	100	S4	LM	126.224783861671
5	H4	120	S1	RF	62.4497094322754
5	H4	120	S2	RF	12.2038444345105
5	H4	120	S3	RF	81.3142601698704
5	H4	120	S4	RF	44.7027268663388
5	H4	120	S1	BB	143.367346938776
5	H4	120	S2	BB	1546.68367346939
5	H4	120	S3	BB	481.632653061225
5	H4	120	S4	BB	80.3571428571429
5	H4	120	S1	FCU	857.055214723926
5	H4	120	S2	FCU	819.325153374233
5	H4	120	S3	FCU	116.257668711657
5	H4	120	S4	FCU	67.1779141104295
5	H4	120	S1	ES	250
5	H4	120	S2	ES	-12.5528913963329
5	H4	120	S3	ES	-18.6177715091678
5	H4	120	S4	ES	102.397743300423
5	H4	120	S1	MG	30.8159136884693
5	H4	120	S2	MG	47.8084962913014
5	H4	120	S3	MG	74.9157113958193
5	H4	120	S4	MG	48.6176668914363
5	H4	120	S1	LM	61.2353567625133
5	H4	120	S2	LM	373.269435569755
5	H4	120	S3	LM	216.613418530351
5	H4	120	S4	LM	92.5452609158679
6	H1	60	S1	RF	24.9596122778675
6	H1	60	S2	RF	-41.1954765751212
6	H1	60	S3	RF	0.646203554119548
6	H1	60	S4	RF	68.7399030694669
6	H1	60	S1	BB	467.563025210084
6	H1	60	S2	BB	647.563025210084
6	H1	60	S3	BB	910.756302521008
6	H1	60	S4	BB	208.571428571429
6	H1	60	S1	FCU	399.470899470899
6	H1	60	S2	FCU	587.830687830688
6	H1	60	S3	FCU	532.539682539683
6	H1	60	S4	FCU	42.3280423280423
6	H1	60	S1	ES	184.641755227974
6	H1	60	S2	ES	-75.659924580048
6	H1	60	S3	ES	-57.8676722660268
6	H1	60	S4	ES	256.907781967775
6	H1	60	S1	MG	233.448275862069
6	H1	60	S2	MG	-30.8620689655172
6	H1	60	S3	MG	68.6206896551724

Continued on next page

Participant_ID	Seat_Height	Seat_Height_pct_PH	Stage	Muscle	Delta_RMS_pct
6	H1	60	S4	MG	323.793103448276
6	H1	60	S1	LM	-3.26996197718633
6	H1	60	S2	LM	11.128010139417
6	H1	60	S3	LM	-4.4106463878327
6	H1	60	S4	LM	-3.97972116603295
6	H2	80	S1	RF	-29.3238993710692
6	H2	80	S2	RF	-50.1572327044025
6	H2	80	S3	RF	-30.5817610062893
6	H2	80	S4	RF	96.8553459119497
6	H2	80	S1	BB	35.6410256410256
6	H2	80	S2	BB	1689.48717948718
6	H2	80	S3	BB	805.384615384616
6	H2	80	S4	BB	55.8974358974359
6	H2	80	S1	FCU	105.369127516779
6	H2	80	S2	FCU	728.187919463087
6	H2	80	S3	FCU	756.040268456376
6	H2	80	S4	FCU	143.959731543624
6	H2	80	S1	ES	35.1171875
6	H2	80	S2	ES	-87.91015625
6	H2	80	S3	ES	-83.73046875
6	H2	80	S4	ES	50.37109375
6	H2	80	S1	MG	82.8105395232121
6	H2	80	S2	MG	-21.0790464240903
6	H2	80	S3	MG	19.1969887076537
6	H2	80	S4	MG	206.524466750314
6	H2	80	S1	LM	-68.0860501219783
6	H2	80	S2	LM	23.8190286094478
6	H2	80	S3	LM	1.2197826569084
6	H2	80	S4	LM	-35.4180527833223
6	H3	100	S1	RF	21.205597416577
6	H3	100	S2	RF	-27.9870828848224
6	H3	100	S3	RF	44.0258342303552
6	H3	100	S4	RF	71.9052744886975
6	H3	100	S1	BB	142.172523961661
6	H3	100	S2	BB	1355.5910543131
6	H3	100	S3	BB	1444.4089456869
6	H3	100	S4	BB	156.549520766773
6	H3	100	S1	FCU	233.744855967078
6	H3	100	S2	FCU	987.654320987654
6	H3	100	S3	FCU	426.748971193416
6	H3	100	S4	FCU	194.238683127572
6	H3	100	S1	ES	-5.27169505271695
6	H3	100	S2	ES	-70.154095701541
6	H3	100	S3	ES	-76.0746147607461
6	H3	100	S4	ES	43.3292781832928
6	H3	100	S1	MG	80.5747126436782
6	H3	100	S2	MG	5.74712643678161
6	H3	100	S3	MG	127.126436781609
6	H3	100	S4	MG	131.724137931035
6	H3	100	S1	LM	-59.6691729323308
6	H3	100	S2	LM	65.6842105263158
6	H3	100	S3	LM	52.2406015037594
6	H3	100	S4	LM	-29.6541353383459
6	H4	120	S1	RF	63.9386189258312
6	H4	120	S2	RF	-11.6368286445013
6	H4	120	S3	RF	93.0946291560102

Continued on next page

Participant_ID	Seat_Height	Seat_Height_pct_PH	Stage	Muscle	Delta_RMS_pct
6	H4	120	S4	RF	134.526854219949
6	H4	120	S1	BB	353.559322033898
6	H4	120	S2	BB	2158.30508474576
6	H4	120	S3	BB	533.220338983051
6	H4	120	S4	BB	29.8305084745763
6	H4	120	S1	FCU	205.208333333333
6	H4	120	S2	FCU	1203.81944444444
6	H4	120	S3	FCU	280.902777777778
6	H4	120	S4	FCU	89.9305555555556
6	H4	120	S1	ES	-33.9892364614867
6	H4	120	S2	ES	-69.8789101917255
6	H4	120	S3	ES	-63.5553313151699
6	H4	120	S4	ES	-14.7830474268416
6	H4	120	S1	MG	249.720670391061
6	H4	120	S2	MG	89.0130353817505
6	H4	120	S3	MG	294.040968342644
6	H4	120	S4	MG	491.247672253259
6	H4	120	S1	LM	-37.7811666031262
6	H4	120	S2	LM	63.9725505146778
6	H4	120	S3	LM	17.4990468928708
6	H4	120	S4	LM	-0.381242851696536
7	H1	60	S1	RF	86.986301369863
7	H1	60	S2	RF	-21.8555417185554
7	H1	60	S3	RF	-12.1419676214197
7	H1	60	S4	RF	53.4246575342466
7	H1	60	S1	BB	785.608856088561
7	H1	60	S2	BB	607.749077490775
7	H1	60	S3	BB	172.324723247232
7	H1	60	S4	BB	729.520295202952
7	H1	60	S1	FCU	125.188227241615
7	H1	60	S2	FCU	215.195071868583
7	H1	60	S3	FCU	167.35112936345
7	H1	60	S4	FCU	1282.06707734429
7	H1	60	S1	ES	-63.8220521406362
7	H1	60	S2	ES	-82.2004305190146
7	H1	60	S3	ES	-90.112413298254
7	H1	60	S4	ES	-83.0184166467352
7	H1	60	S1	MG	146.145251396648
7	H1	60	S2	MG	-28.3798882681564
7	H1	60	S3	MG	-20.5586592178771
7	H1	60	S4	MG	246.815642458101
7	H1	60	S1	LM	-8.42343733909998
7	H1	60	S2	LM	-49.6344351766039
7	H1	60	S3	LM	-74.4619503655648
7	H1	60	S4	LM	-0.803212851405624
7	H2	80	S1	RF	22.0813875917278
7	H2	80	S2	RF	-6.27084723148766
7	H2	80	S3	RF	34.8232154769847
7	H2	80	S4	RF	39.2261507671781
7	H2	80	S1	BB	49.6782496782497
7	H2	80	S2	BB	624.066924066924
7	H2	80	S3	BB	165.765765765766
7	H2	80	S4	BB	59.7168597168597
7	H2	80	S1	FCU	143.008225616921
7	H2	80	S2	FCU	872.385428907168
7	H2	80	S3	FCU	275.793184488837

Continued on next page

Participant_ID	Seat_Height	Seat_Height_pct_PH	Stage	Muscle	Delta_RMS_pct
7	H2	80	S4	FCU	223.266745005875
7	H2	80	S1	ES	9.66232029421597
7	H2	80	S2	ES	28.1511200267469
7	H2	80	S3	ES	29.9231026412571
7	H2	80	S4	ES	33.4670678702775
7	H2	80	S1	MG	75.6608933454877
7	H2	80	S2	MG	41.3855970829535
7	H2	80	S3	MG	63.5369188696445
7	H2	80	S4	MG	279.671832269827
7	H2	80	S1	LM	-11.3245437876539
7	H2	80	S2	LM	-51.8816030734305
7	H2	80	S3	LM	28.0101283506505
7	H2	80	S4	LM	89.8279926656771
7	H3	100	S1	RF	18.3561643835617
7	H3	100	S2	RF	-11.3013698630137
7	H3	100	S3	RF	10.8904109589041
7	H3	100	S4	RF	29.1095890410959
7	H3	100	S1	BB	195.983935742972
7	H3	100	S2	BB	806.024096385542
7	H3	100	S3	BB	451.40562248996
7	H3	100	S4	BB	103.614457831325
7	H3	100	S1	FCU	186.903304773562
7	H3	100	S2	FCU	599.143206854345
7	H3	100	S3	FCU	284.210526315789
7	H3	100	S4	FCU	139.657282741738
7	H3	100	S1	ES	-14.0925234495948
7	H3	100	S2	ES	-71.9469993625353
7	H3	100	S3	ES	-84.163555231764
7	H3	100	S4	ES	-41.7448319825153
7	H3	100	S1	MG	82.2033898305085
7	H3	100	S2	MG	37.0056497175141
7	H3	100	S3	MG	28.6723163841808
7	H3	100	S4	MG	129.661016949153
7	H3	100	S1	LM	226.457066587572
7	H3	100	S2	LM	-26.6498591131544
7	H3	100	S3	LM	-33.3531069257007
7	H3	100	S4	LM	28.7112561174551
7	H4	120	S1	RF	-3.08641975308641
7	H4	120	S2	RF	17.6638176638177
7	H4	120	S3	RF	27.7302943969611
7	H4	120	S4	RF	3.65622032288699
7	H4	120	S1	BB	63.9871382636656
7	H4	120	S2	BB	44.3729903536978
7	H4	120	S3	BB	184.726688102894
7	H4	120	S4	BB	40.5144694533762
7	H4	120	S1	FCU	301.946721311475
7	H4	120	S2	FCU	176.639344262295
7	H4	120	S3	FCU	320.799180327869
7	H4	120	S4	FCU	30.6352459016394
7	H4	120	S1	ES	-61.4400476048795
7	H4	120	S2	ES	-67.3906575423981
7	H4	120	S3	ES	-75.5429931567986
7	H4	120	S4	ES	-29.3811365664981
7	H4	120	S1	MG	-44.5411392405063
7	H4	120	S2	MG	-27.9272151898734
7	H4	120	S3	MG	18.8291139240506

Continued on next page

Participant_ID	Seat_Height	Seat_Height_pct_PH	Stage	Muscle	Delta_RMS_pct
7	H4	120	S4	MG	54.9841772151899
7	H4	120	S1	LM	-75.492518032081
7	H4	120	S2	LM	-22.5374098395952
7	H4	120	S3	LM	-42.8571428571429
7	H4	120	S4	LM	-42.173538594036
8	H1	60	S1	RF	540.16393442623
8	H1	60	S2	RF	14.1803278688525
8	H1	60	S3	RF	300.573770491803
8	H1	60	S4	RF	54.016393442623
8	H1	60	S1	BB	686.111111111111
8	H1	60	S2	BB	2676.38888888889
8	H1	60	S3	BB	765.625
8	H1	60	S4	BB	1360.06944444444
8	H1	60	S1	FCU	52.2172949002218
8	H1	60	S2	FCU	1006.65188470067
8	H1	60	S3	FCU	646.89578713969
8	H1	60	S4	FCU	44.5676274944568
8	H1	60	S1	ES	340.320082601962
8	H1	60	S2	ES	-51.4197212183789
8	H1	60	S3	ES	25.9163655136809
8	H1	60	S4	ES	458.027878162106
8	H1	60	S1	MG	377.142857142857
8	H1	60	S2	MG	127.142857142857
8	H1	60	S3	MG	105.357142857143
8	H1	60	S4	MG	472.5
8	H1	60	S1	LM	14.9708360337006
8	H1	60	S2	LM	124.659753726507
8	H1	60	S3	LM	111.211924821776
8	H1	60	S4	LM	-36.6817887232664
8	H2	80	S1	RF	256.18073316283
8	H2	80	S2	RF	31.4578005115089
8	H2	80	S3	RF	42.7962489343563
8	H2	80	S4	RF	42.2847399829497
8	H2	80	S1	BB	230.49853372434
8	H2	80	S2	BB	2051.90615835777
8	H2	80	S3	BB	1224.04692082111
8	H2	80	S4	BB	215.24926686217
8	H2	80	S1	FCU	-42.3327895595432
8	H2	80	S2	FCU	832.381729200653
8	H2	80	S3	FCU	-2.44698205546492
8	H2	80	S4	FCU	-16.9657422512235
8	H2	80	S1	ES	316.583333333333
8	H2	80	S2	ES	-70
8	H2	80	S3	ES	-73.3333333333333
8	H2	80	S4	ES	206.791666666667
8	H2	80	S1	MG	133.585619678335
8	H2	80	S2	MG	195.26963103122
8	H2	80	S3	MG	-9.17691579943236
8	H2	80	S4	MG	215.610217596973
8	H2	80	S1	LM	-48.3722576079264
8	H2	80	S2	LM	135.244161358811
8	H2	80	S3	LM	-35.3503184713376
8	H2	80	S4	LM	5.41401273885349
8	H3	100	S1	RF	65.7414750198255
8	H3	100	S2	RF	58.2870737509913
8	H3	100	S3	RF	103.409992069786

Continued on next page

Participant_ID	Seat_Height	Seat_Height_pct_PH	Stage	Muscle	Delta_RMS_pct
8	H3	100	S4	RF	66.4551942902458
8	H3	100	S1	BB	73.4513274336283
8	H3	100	S2	BB	1573.89380530973
8	H3	100	S3	BB	304.424778761062
8	H3	100	S4	BB	60.3982300884956
8	H3	100	S1	FCU	119.32059447983
8	H3	100	S2	FCU	2652.01698513801
8	H3	100	S3	FCU	94.0552016985138
8	H3	100	S4	FCU	52.2292993630573
8	H3	100	S1	ES	248.775247099269
8	H3	100	S2	ES	-63.5152556940266
8	H3	100	S3	ES	-80.4469273743017
8	H3	100	S4	ES	79.0717662226042
8	H3	100	S1	MG	204.09429280397
8	H3	100	S2	MG	98.6352357320099
8	H3	100	S3	MG	74.9379652605459
8	H3	100	S4	MG	283.374689826303
8	H3	100	S1	LM	-39.9557277255119
8	H3	100	S2	LM	306.917542888766
8	H3	100	S3	LM	-46.5965688987272
8	H3	100	S4	LM	6.08743774211401
8	H4	120	S1	RF	-38.2768361581921
8	H4	120	S2	RF	-42.090395480226
8	H4	120	S3	RF	-61.3465160075329
8	H4	120	S4	RF	-22.2222222222222
8	H4	120	S1	BB	-6.47482014388489
8	H4	120	S2	BB	2829.25659472422
8	H4	120	S3	BB	297.841726618705
8	H4	120	S4	BB	105.275779376499
8	H4	120	S1	FCU	43.5087719298245
8	H4	120	S2	FCU	1984.21052631579
8	H4	120	S3	FCU	263.859649122807
8	H4	120	S4	FCU	159.298245614035
8	H4	120	S1	ES	86.3277826468011
8	H4	120	S2	ES	-17.0026292725679
8	H4	120	S3	ES	-45.5740578439965
8	H4	120	S4	ES	104.820333041192
8	H4	120	S1	MG	548.960739030023
8	H4	120	S2	MG	167.898383371825
8	H4	120	S3	MG	139.491916859122
8	H4	120	S4	MG	680.13856812933
8	H4	120	S1	LM	-39.6290873596877
8	H4	120	S2	LM	264.812103465105
8	H4	120	S3	LM	2.29380185456321
8	H4	120	S4	LM	-14.5436798438262
9	H1	60	S1	RF	47.8260869565217
9	H1	60	S2	RF	4.68227424749163
9	H1	60	S3	RF	-2.34113712374583
9	H1	60	S4	RF	68.2274247491639
9	H1	60	S1	BB	614.666666666667
9	H1	60	S2	BB	1033.77777777778
9	H1	60	S3	BB	551.555555555556
9	H1	60	S4	BB	261.777777777778
9	H1	60	S1	FCU	702.332361516035
9	H1	60	S2	FCU	1319.8250728863
9	H1	60	S3	FCU	281.34110787172

Continued on next page

Participant_ID	Seat_Height	Seat_Height_pct_PH	Stage	Muscle	Delta_RMS_pct
9	H1	60	S4	FCU	1217.20116618076
9	H1	60	S1	ES	119.269521410579
9	H1	60	S2	ES	-45.7178841309824
9	H1	60	S3	ES	-55.4156171284635
9	H1	60	S4	ES	241.435768261965
9	H1	60	S1	MG	129.280397022332
9	H1	60	S2	MG	28.287841191067
9	H1	60	S3	MG	9.42928039702233
9	H1	60	S4	MG	93.5483870967742
9	H1	60	S1	LM	38.6400431732326
9	H1	60	S2	LM	99.5143011332973
9	H1	60	S3	LM	59.1473286562331
9	H1	60	S4	LM	14.5709660010793
9	H2	80	S1	RF	68.4981684981685
9	H2	80	S2	RF	2.93040293040293
9	H2	80	S3	RF	20.3296703296703
9	H2	80	S4	RF	124.908424908425
9	H2	80	S1	BB	266.783216783217
9	H2	80	S2	BB	299.300699300699
9	H2	80	S3	BB	366.433566433567
9	H2	80	S4	BB	284.965034965035
9	H2	80	S1	FCU	318.693693693694
9	H2	80	S2	FCU	1196.3963963964
9	H2	80	S3	FCU	597.072072072072
9	H2	80	S4	FCU	536.036036036036
9	H2	80	S1	ES	175.935828877005
9	H2	80	S2	ES	-39.9732620320856
9	H2	80	S3	ES	139.973262032086
9	H2	80	S4	ES	230.882352941176
9	H2	80	S1	MG	32.3913043478261
9	H2	80	S2	MG	-12.3913043478261
9	H2	80	S3	MG	30.4347826086957
9	H2	80	S4	MG	93.6956521739131
9	H2	80	S1	LM	37.2862029646522
9	H2	80	S2	LM	493.614595210947
9	H2	80	S3	LM	839.110604332953
9	H2	80	S4	LM	110.262257696693
9	H3	100	S1	RF	39.6959459459459
9	H3	100	S2	RF	-6.58783783783783
9	H3	100	S3	RF	2.02702702702703
9	H3	100	S4	RF	65.8783783783784
9	H3	100	S1	BB	311.344537815126
9	H3	100	S2	BB	940.756302521008
9	H3	100	S3	BB	657.983193277311
9	H3	100	S4	BB	164.285714285714
9	H3	100	S1	FCU	825.2
9	H3	100	S2	FCU	1365.2
9	H3	100	S3	FCU	1391.6
9	H3	100	S4	FCU	683.6
9	H3	100	S1	ES	56.9757727652464
9	H3	100	S2	ES	4.92898913951545
9	H3	100	S3	ES	122.222222222222
9	H3	100	S4	ES	129.490392648287
9	H3	100	S1	MG	5.28634361233481
9	H3	100	S2	MG	-1.98237885462555
9	H3	100	S3	MG	73.1277533039648

Continued on next page

Participant_ID	Seat_Height	Seat_Height_pct_PH	Stage	Muscle	Delta_RMS_pct
9	H3	100	S4	MG	50.2202643171806
9	H3	100	S1	LM	-53.7447988904299
9	H3	100	S2	LM	206.24133148405
9	H3	100	S3	LM	305.201109570042
9	H3	100	S4	LM	-28.502080443828
9	H4	120	S1	RF	85.0287907869482
9	H4	120	S2	RF	79.2706333973129
9	H4	120	S3	RF	6.90978886756239
9	H4	120	S4	RF	85.2207293666027
9	H4	120	S1	BB	83.1775700934579
9	H4	120	S2	BB	517.133956386293
9	H4	120	S3	BB	494.704049844237
9	H4	120	S4	BB	17.7570093457944
9	H4	120	S1	FCU	64.5865834633385
9	H4	120	S2	FCU	968.330733229329
9	H4	120	S3	FCU	710.296411856474
9	H4	120	S4	FCU	20.5928237129485
9	H4	120	S1	ES	129.126213592233
9	H4	120	S2	ES	183.171521035599
9	H4	120	S3	ES	370.226537216828
9	H4	120	S4	ES	380.744336569579
9	H4	120	S1	MG	159.125964010283
9	H4	120	S2	MG	18.2519280205655
9	H4	120	S3	MG	30.5912596401028
9	H4	120	S4	MG	88.6889460154242
9	H4	120	S1	LM	107.580824972129
9	H4	120	S2	LM	467.335562987737
9	H4	120	S3	LM	161.426978818283
9	H4	120	S4	LM	24.5261984392419
10	H1	60	S1	RF	154.212454212454
10	H1	60	S2	RF	54.8229548229548
10	H1	60	S3	RF	135.286935286935
10	H1	60	S4	RF	400.976800976801
10	H1	60	S1	BB	43.3333333333333
10	H1	60	S2	BB	791.25
10	H1	60	S3	BB	637.083333333333
10	H1	60	S4	BB	95.8333333333333
10	H1	60	S1	FCU	10.250569476082
10	H1	60	S2	FCU	1452.39179954442
10	H1	60	S3	FCU	775.626423690205
10	H1	60	S4	FCU	155.353075170843
10	H1	60	S1	ES	331.215846994536
10	H1	60	S2	ES	82.9918032786885
10	H1	60	S3	ES	45.6284153005464
10	H1	60	S4	ES	669.672131147541
10	H1	60	S1	MG	359.375
10	H1	60	S2	MG	103.3203125
10	H1	60	S3	MG	71.09375
10	H1	60	S4	MG	471.2890625
10	H1	60	S1	LM	-71.7770655270655
10	H1	60	S2	LM	8.67165242165243
10	H1	60	S3	LM	-56.6417378917379
10	H1	60	S4	LM	-39.0491452991453
10	H2	80	S1	RF	64.5598194130926
10	H2	80	S2	RF	-22.7990970654627
10	H2	80	S3	RF	103.085026335591

Continued on next page

Participant_ID	Seat_Height	Seat_Height_pct_PH	Stage	Muscle	Delta_RMS_pct
10	H2	80	S4	RF	144.394281414597
10	H2	80	S1	BB	85.8895705521472
10	H2	80	S2	BB	934.969325153374
10	H2	80	S3	BB	255.828220858896
10	H2	80	S4	BB	138.036809815951
10	H2	80	S1	FCU	40.6593406593407
10	H2	80	S2	FCU	3953.84615384615
10	H2	80	S3	FCU	1395.60439560439
10	H2	80	S4	FCU	107.692307692308
10	H2	80	S1	ES	123.859144807761
10	H2	80	S2	ES	-11.103126122889
10	H2	80	S3	ES	37.1900826446281
10	H2	80	S4	ES	222.673374056773
10	H2	80	S1	MG	244.318181818182
10	H2	80	S2	MG	371.915584415584
10	H2	80	S3	MG	122.88961038961
10	H2	80	S4	MG	483.279220779221
10	H2	80	S1	LM	3.37448559670782
10	H2	80	S2	LM	295.555555555555
10	H2	80	S3	LM	138.683127572017
10	H2	80	S4	LM	150.699588477366
10	H3	100	S1	RF	83.7579617834395
10	H3	100	S2	RF	116.772823779193
10	H3	100	S3	RF	106.050955414013
10	H3	100	S4	RF	203.821656050956
10	H3	100	S1	BB	42.0353982300885
10	H3	100	S2	BB	382.743362831859
10	H3	100	S3	BB	163.274336283186
10	H3	100	S4	BB	44.6902654867257
10	H3	100	S1	FCU	63.0541871921182
10	H3	100	S2	FCU	1704.43349753695
10	H3	100	S3	FCU	338.423645320197
10	H3	100	S4	FCU	66.9950738916256
10	H3	100	S1	ES	470.191431175934
10	H3	100	S2	ES	191.886964448496
10	H3	100	S3	ES	79.3983591613491
10	H3	100	S4	ES	444.849589790337
10	H3	100	S1	MG	341.607142857143
10	H3	100	S2	MG	423.392857142857
10	H3	100	S3	MG	189.464285714286
10	H3	100	S4	MG	155.892857142857
10	H3	100	S1	LM	28.8311688311688
10	H3	100	S2	LM	165.142857142857
10	H3	100	S3	LM	-28.2077922077922
10	H3	100	S4	LM	26.1298701298701
10	H4	120	S1	RF	77.5045537340619
10	H4	120	S2	RF	71.8579234972678
10	H4	120	S3	RF	58.287795992714
10	H4	120	S4	RF	135.974499089253
10	H4	120	S1	BB	36.7713004484305
10	H4	120	S2	BB	999.103139013453
10	H4	120	S3	BB	154.260089686099
10	H4	120	S4	BB	56.5022421524664
10	H4	120	S1	FCU	20.4347826086957
10	H4	120	S2	FCU	3410.86956521739
10	H4	120	S3	FCU	106.521739130435

Continued on next page

Participant_ID	Seat_Height	Seat_Height_pct_PH	Stage	Muscle	Delta_RMS_pct
10	H4	120	S4	FCU	50.4347826086957
10	H4	120	S1	ES	434.529702970297
10	H4	120	S2	ES	525.618811881188
10	H4	120	S3	ES	263.861386138614
10	H4	120	S4	ES	562.995049504951
10	H4	120	S1	MG	219.844357976654
10	H4	120	S2	MG	207.782101167315
10	H4	120	S3	MG	160.570687418936
10	H4	120	S4	MG	194.163424124514
10	H4	120	S1	LM	-5.95802301963439
10	H4	120	S2	LM	239.810426540284
10	H4	120	S3	LM	11.5775220040623
10	H4	120	S4	LM	25.727826675694
11	H1	60	S1	RF	-41.0194174757281
11	H1	60	S2	RF	-55.0364077669903
11	H1	60	S3	RF	-50.6067961165049
11	H1	60	S4	RF	26.6383495145631
11	H1	60	S1	BB	343.680709534368
11	H1	60	S2	BB	405.543237250554
11	H1	60	S3	BB	1085.36585365854
11	H1	60	S4	BB	191.79600886918
11	H1	60	S1	FCU	132.575757575758
11	H1	60	S2	FCU	151.041666666667
11	H1	60	S3	FCU	183.238636363636
11	H1	60	S4	FCU	278.125
11	H1	60	S1	ES	158.644656820156
11	H1	60	S2	ES	-43.0060816681147
11	H1	60	S3	ES	14.0747176368375
11	H1	60	S4	ES	289.748045178106
11	H1	60	S1	MG	144.352331606218
11	H1	60	S2	MG	747.150259067357
11	H1	60	S3	MG	-11.2953367875648
11	H1	60	S4	MG	259.067357512953
11	H1	60	S1	LM	119.915890657855
11	H1	60	S2	LM	-23.9110844097327
11	H1	60	S3	LM	-38.2697506758786
11	H1	60	S4	LM	-5.7374586963052
11	H2	80	S1	RF	-30.6592039800995
11	H2	80	S2	RF	-60.3855721393035
11	H2	80	S3	RF	-63.1218905472637
11	H2	80	S4	RF	-4.6641791044776
11	H2	80	S1	BB	109.351145038168
11	H2	80	S2	BB	610.30534351145
11	H2	80	S3	BB	501.717557251908
11	H2	80	S4	BB	164.312977099237
11	H2	80	S1	FCU	164.387917329094
11	H2	80	S2	FCU	427.662957074722
11	H2	80	S3	FCU	82.670906200318
11	H2	80	S4	FCU	46.2639109697933
11	H2	80	S1	ES	164.158918005072
11	H2	80	S2	ES	-15.3000845308538
11	H2	80	S3	ES	5.24091293322062
11	H2	80	S4	ES	159.256128486898
11	H2	80	S1	MG	-17.7059019673224
11	H2	80	S2	MG	57.6192064021341
11	H2	80	S3	MG	-71.457152384128

Continued on next page

Participant_ID	Seat_Height	Seat_Height_pct_PH	Stage	Muscle	Delta_RMS_pct
11	H2	80	S4	MG	29.8766255418473
11	H2	80	S1	LM	-12.8950695322377
11	H2	80	S2	LM	26.9279393173198
11	H2	80	S3	LM	277.24399494311
11	H2	80	S4	LM	90.3919089759798
11	H3	100	S1	RF	-21.8181818181818
11	H3	100	S2	RF	102.770562770563
11	H3	100	S3	RF	96.6233766233766
11	H3	100	S4	RF	92.7272727272727
11	H3	100	S1	BB	57.356608478803
11	H3	100	S2	BB	928.927680798005
11	H3	100	S3	BB	364.588528678304
11	H3	100	S4	BB	249.376558603491
11	H3	100	S1	FCU	456.349206349206
11	H3	100	S2	FCU	1192.85714285714
11	H3	100	S3	FCU	383.730158730159
11	H3	100	S4	FCU	926.587301587302
11	H3	100	S1	ES	117.820658342792
11	H3	100	S2	ES	59.2508513053348
11	H3	100	S3	ES	-25.539160045403
11	H3	100	S4	ES	318.728717366629
11	H3	100	S1	MG	0.212879191059069
11	H3	100	S2	MG	-0.851516764236297
11	H3	100	S3	MG	-1.64981373070782
11	H3	100	S4	MG	8.51516764236297
11	H3	100	S1	LM	27.2309107635695
11	H3	100	S2	LM	75.6209751609936
11	H3	100	S3	LM	-19.8712051517939
11	H3	100	S4	LM	159.153633854646
11	H4	120	S1	RF	-48.1194372667241
11	H4	120	S2	RF	-57.70887166236
11	H4	120	S3	RF	-14.6999712891186
11	H4	120	S4	RF	57.4217628481194
11	H4	120	S1	BB	139.628482972136
11	H4	120	S2	BB	1008.66873065016
11	H4	120	S3	BB	1672.44582043344
11	H4	120	S4	BB	185.758513931889
11	H4	120	S1	FCU	380.444856348471
11	H4	120	S2	FCU	656.441149212234
11	H4	120	S3	FCU	996.570898980538
11	H4	120	S4	FCU	165.060240963855
11	H4	120	S1	ES	-51.2746899402848
11	H4	120	S2	ES	-21.0610932475884
11	H4	120	S3	ES	-38.2521819016996
11	H4	120	S4	ES	-45.016077170418
11	H4	120	S1	MG	270.541082164329
11	H4	120	S2	MG	191.783567134269
11	H4	120	S3	MG	237.4749498998
11	H4	120	S4	MG	401.803607214429
11	H4	120	S1	LM	19.7102425876011
11	H4	120	S2	LM	81.6711590296496
11	H4	120	S3	LM	43.0929919137466
11	H4	120	S4	LM	87.7358490566038
12	H1	60	S1	RF	332.656514382403
12	H1	60	S2	RF	203.722504230118
12	H1	60	S3	RF	89.678510998308

Continued on next page

Participant_ID	Seat_Height	Seat_Height_pct_PH	Stage	Muscle	Delta_RMS_pct
12	H1	60	S4	RF	275.972927241963
12	H1	60	S1	BB	263.612565445026
12	H1	60	S2	BB	1223.56020942408
12	H1	60	S3	BB	445.549738219895
12	H1	60	S4	BB	172.251308900524
12	H1	60	S1	FCU	53.4201954397394
12	H1	60	S2	FCU	1474.2671009772
12	H1	60	S3	FCU	330.293159609121
12	H1	60	S4	FCU	19.5439739413681
12	H1	60	S1	ES	331.07476635514
12	H1	60	S2	ES	-29.2056074766355
12	H1	60	S3	ES	-44.7429906542056
12	H1	60	S4	ES	406.542056074766
12	H1	60	S1	MG	574.232081911263
12	H1	60	S2	MG	18.259385665529
12	H1	60	S3	MG	43.6860068259386
12	H1	60	S4	MG	472.354948805461
12	H1	60	S1	LM	-25.8513053348468
12	H1	60	S2	LM	46.311010215664
12	H1	60	S3	LM	21.4245175936436
12	H1	60	S4	LM	-34.4494892167991
12	H2	80	S1	RF	216.211878009631
12	H2	80	S2	RF	112.038523274478
12	H2	80	S3	RF	171.268057784912
12	H2	80	S4	RF	133.22632423756
12	H2	80	S1	BB	106.578947368421
12	H2	80	S2	BB	1100.65789473684
12	H2	80	S3	BB	999.342105263158
12	H2	80	S4	BB	129.605263157895
12	H2	80	S1	FCU	29.0657439446367
12	H2	80	S2	FCU	1384.0830449827
12	H2	80	S3	FCU	1082.69896193772
12	H2	80	S4	FCU	70.9342560553633
12	H2	80	S1	ES	132.562076749436
12	H2	80	S2	ES	-18.1715575620767
12	H2	80	S3	ES	-52.313769751693
12	H2	80	S4	ES	117.437923250564
12	H2	80	S1	MG	791.681735985533
12	H2	80	S2	MG	224.231464737794
12	H2	80	S3	MG	159.855334538879
12	H2	80	S4	MG	441.772151898734
12	H2	80	S1	LM	-23.70942812983
12	H2	80	S2	LM	48.5316846986089
12	H2	80	S3	LM	62.3183925811437
12	H2	80	S4	LM	28.8408037094281
12	H3	100	S1	RF	390.992366412214
12	H3	100	S2	RF	149.465648854962
12	H3	100	S3	RF	137.862595419847
12	H3	100	S4	RF	140.916030534351
12	H3	100	S1	BB	9.46291560102302
12	H3	100	S2	BB	894.62915601023
12	H3	100	S3	BB	562.148337595908
12	H3	100	S4	BB	92.0716112531969
12	H3	100	S1	FCU	-0.701754385964913
12	H3	100	S2	FCU	1511.57894736842
12	H3	100	S3	FCU	1070.52631578947

Continued on next page

Participant_ID	Seat_Height	Seat_Height_pct_PH	Stage	Muscle	Delta_RMS_pct
12	H3	100	S4	FCU	65.9649122807018
12	H3	100	S1	ES	95.5145118733509
12	H3	100	S2	ES	12.9947229551451
12	H3	100	S3	ES	-12.532981530343
12	H3	100	S4	ES	151.97889182058
12	H3	100	S1	MG	353.430821147356
12	H3	100	S2	MG	31.2710911136108
12	H3	100	S3	MG	55.793025871766
12	H3	100	S4	MG	204.949381327334
12	H3	100	S1	LM	-67.003367003367
12	H3	100	S2	LM	62.5252525252525
12	H3	100	S3	LM	111.952861952862
12	H3	100	S4	LM	0.875420875420881
12	H4	120	S1	RF	269.918699186992
12	H4	120	S2	RF	207.926829268293
12	H4	120	S3	RF	331.910569105691
12	H4	120	S4	RF	543.69918699187
12	H4	120	S1	BB	69.3939393939394
12	H4	120	S2	BB	510.30303030303
12	H4	120	S3	BB	655.151515151515
12	H4	120	S4	BB	76.3636363636364
12	H4	120	S1	FCU	4.7457627118644
12	H4	120	S2	FCU	1332.54237288136
12	H4	120	S3	FCU	1215.59322033898
12	H4	120	S4	FCU	34.9152542372881
12	H4	120	S1	ES	259.709241952233
12	H4	120	S2	ES	89.7196261682243
12	H4	120	S3	ES	121.391484942887
12	H4	120	S4	ES	277.673935617861
12	H4	120	S1	MG	899.374021909233
12	H4	120	S2	MG	179.81220657277
12	H4	120	S3	MG	237.245696400626
12	H4	120	S4	MG	290.766823161189
12	H4	120	S1	LM	-33.9347675225538
12	H4	120	S2	LM	297.848716169327
12	H4	120	S3	LM	319.361554476058
12	H4	120	S4	LM	86.6065232477446
13	H1	60	S1	RF	135.060639470783
13	H1	60	S2	RF	-31.6427783902977
13	H1	60	S3	RF	94.3770672546858
13	H1	60	S4	RF	184.564498346196
13	H1	60	S1	BB	-38.1781981085117
13	H1	60	S2	BB	58.2877053260329
13	H1	60	S3	BB	22.2498755599801
13	H1	60	S4	BB	-22.84718765555
13	H1	60	S1	FCU	-28.8924194280527
13	H1	60	S2	FCU	67.9073990013618
13	H1	60	S3	FCU	61.9609623241035
13	H1	60	S4	FCU	-37.2900590104403
13	H1	60	S1	ES	888.523610280932
13	H1	60	S2	ES	-65.9294680215182
13	H1	60	S3	ES	-68.5594739988045
13	H1	60	S4	ES	167.872086072923
13	H1	60	S1	MG	137.033398821218
13	H1	60	S2	MG	-49.1159135559921
13	H1	60	S3	MG	65.4223968565815

Continued on next page

Participant_ID	Seat_Height	Seat_Height_pct_PH	Stage	Muscle	Delta_RMS_pct
13	H1	60	S4	MG	269.941060903733
13	H1	60	S1	LM	-64.9143717458127
13	H1	60	S2	LM	-73.5963866758673
13	H1	60	S3	LM	-61.1065805156515
13	H1	60	S4	LM	-79.7503293394392
13	H2	80	S1	RF	-34.631700732742
13	H2	80	S2	RF	-22.6764365599691
13	H2	80	S3	RF	-50.8291554184342
13	H2	80	S4	RF	-3.35518704203625
13	H2	80	S1	BB	-7.6923076923077
13	H2	80	S2	BB	100.160256410256
13	H2	80	S3	BB	113.060897435897
13	H2	80	S4	BB	-4.6474358974359
13	H2	80	S1	FCU	848.015122873346
13	H2	80	S2	FCU	380.340264650284
13	H2	80	S3	FCU	334.971644612476
13	H2	80	S4	FCU	174.291115311909
13	H2	80	S1	ES	182.471482889734
13	H2	80	S2	ES	-55.2471482889734
13	H2	80	S3	ES	-11.1787072243346
13	H2	80	S4	ES	632.471482889734
13	H2	80	S1	MG	76.8192048012003
13	H2	80	S2	MG	-38.9347336834209
13	H2	80	S3	MG	-46.5116279069767
13	H2	80	S4	MG	143.885971492873
13	H2	80	S1	LM	-79.7794904731321
13	H2	80	S2	LM	-76.9642474844787
13	H2	80	S3	LM	-35.103118532791
13	H2	80	S4	LM	-80.4538642688932
13	H3	100	S1	RF	-22.0379146919431
13	H3	100	S2	RF	-3.90995260663507
13	H3	100	S3	RF	11.4928909952607
13	H3	100	S4	RF	34.5379146919431
13	H3	100	S1	BB	54.5045045045045
13	H3	100	S2	BB	686.711711711712
13	H3	100	S3	BB	413.288288288288
13	H3	100	S4	BB	178.153153153153
13	H3	100	S1	FCU	-78.2496413199426
13	H3	100	S2	FCU	30.6743185078909
13	H3	100	S3	FCU	23.012912482066
13	H3	100	S4	FCU	-75.5236728837877
13	H3	100	S1	ES	-47.9344729344729
13	H3	100	S2	ES	-13.0484330484331
13	H3	100	S3	ES	-44.045584045584
13	H3	100	S4	ES	-49.2165242165242
13	H3	100	S1	MG	-18.0780780780781
13	H3	100	S2	MG	28.948948948949
13	H3	100	S3	MG	21.0810810810811
13	H3	100	S4	MG	58.7387387387388
13	H3	100	S1	LM	88.0022637238257
13	H3	100	S2	LM	406.196943972835
13	H3	100	S3	LM	444.567062818336
13	H3	100	S4	LM	503.508771929824
13	H4	120	S1	RF	53.5310734463277
13	H4	120	S2	RF	138.276836158192
13	H4	120	S3	RF	191.80790960452

Continued on next page

Participant_ID	Seat_Height	Seat_Height_pct_PH	Stage	Muscle	Delta_RMS_pct
13	H4	120	S4	RF	167.937853107345
13	H4	120	S1	BB	3.48387096774193
13	H4	120	S2	BB	188.903225806452
13	H4	120	S3	BB	53.8064516129032
13	H4	120	S4	BB	-3.48387096774193
13	H4	120	S1	FCU	48.9859594383775
13	H4	120	S2	FCU	699.531981279251
13	H4	120	S3	FCU	407.644305772231
13	H4	120	S4	FCU	40.7176287051482
13	H4	120	S1	ES	187.155963302752
13	H4	120	S2	ES	189.646133682831
13	H4	120	S3	ES	52.1625163826999
13	H4	120	S4	ES	348.885976408912
13	H4	120	S1	MG	113.77331420373
13	H4	120	S2	MG	89.8134863701578
13	H4	120	S3	MG	302.725968436155
13	H4	120	S4	MG	204.447632711621
13	H4	120	S1	LM	-60.5559025608994
13	H4	120	S2	LM	-17.738913179263
13	H4	120	S3	LM	-38.9756402248595
13	H4	120	S4	LM	-67.7076826983135
14	H1	60	S1	RF	238.213914849429
14	H1	60	S2	RF	5.81516095534786
14	H1	60	S3	RF	68.951194184839
14	H1	60	S4	RF	162.097611630322
14	H1	60	S1	BB	807.993474714519
14	H1	60	S2	BB	1625.12234910277
14	H1	60	S3	BB	1564.92659053834
14	H1	60	S4	BB	1863.94779771615
14	H1	60	S1	FCU	372.199170124481
14	H1	60	S2	FCU	1460.16597510373
14	H1	60	S3	FCU	547.579529737206
14	H1	60	S4	FCU	389.349930843707
14	H1	60	S1	ES	308.773045136681
14	H1	60	S2	ES	-58.6141131595677
14	H1	60	S3	ES	7.94659885568976
14	H1	60	S4	ES	223.140495867768
14	H1	60	S1	MG	843.565147881695
14	H1	60	S2	MG	5.35571542765787
14	H1	60	S3	MG	-37.330135891287
14	H1	60	S4	MG	317.186250999201
14	H1	60	S1	LM	-10.3259726603575
14	H1	60	S2	LM	-42.4815983175604
14	H1	60	S3	LM	-26.8980021030494
14	H1	60	S4	LM	9.25341745531021
14	H2	80	S1	RF	137.47572815534
14	H2	80	S2	RF	76.3106796116505
14	H2	80	S3	RF	29.8058252427184
14	H2	80	S4	RF	212.233009708738
14	H2	80	S1	BB	596.808510638298
14	H2	80	S2	BB	1785.28368794326
14	H2	80	S3	BB	699.822695035461
14	H2	80	S4	BB	1213.47517730496
14	H2	80	S1	FCU	190.694444444445
14	H2	80	S2	FCU	960.277777777778
14	H2	80	S3	FCU	690.833333333333

Continued on next page

Participant_ID	Seat_Height	Seat_Height_pct_PH	Stage	Muscle	Delta_RMS_pct
14	H2	80	S4	FCU	785.277777777778
14	H2	80	S1	ES	339.68253968254
14	H2	80	S2	ES	-48.5569985569986
14	H2	80	S3	ES	274.386724386724
14	H2	80	S4	ES	328.210678210678
14	H2	80	S1	MG	505.62091503268
14	H2	80	S2	MG	63.2679738562092
14	H2	80	S3	MG	147.843137254902
14	H2	80	S4	MG	582.875816993464
14	H2	80	S1	LM	182.28055783429
14	H2	80	S2	LM	-10.2543068088597
14	H2	80	S3	LM	28.0968006562756
14	H2	80	S4	LM	226.497128794093
14	H3	100	S1	RF	33.4519572953737
14	H3	100	S2	RF	62.3665480427046
14	H3	100	S3	RF	87.2775800711744
14	H3	100	S4	RF	151.601423487545
14	H3	100	S1	BB	1336.78532901834
14	H3	100	S2	BB	805.501618122977
14	H3	100	S3	BB	1209.49298813377
14	H3	100	S4	BB	2116.07335490831
14	H3	100	S1	FCU	88.599348534202
14	H3	100	S2	FCU	481.867535287731
14	H3	100	S3	FCU	777.850162866449
14	H3	100	S4	FCU	293.485342019544
14	H3	100	S1	ES	134.213547646383
14	H3	100	S2	ES	-49.7703788748565
14	H3	100	S3	ES	51.9517795637198
14	H3	100	S4	ES	190.52812858783
14	H3	100	S1	MG	-2.86184210526315
14	H3	100	S2	MG	-19.2434210526316
14	H3	100	S3	MG	-49.3421052631579
14	H3	100	S4	MG	-2.89473684210526
14	H3	100	S1	LM	-30.0768122899664
14	H3	100	S2	LM	-29.9807969275084
14	H3	100	S3	LM	15.0504080652904
14	H3	100	S4	LM	-25.8521363418147
14	H4	120	S1	RF	17.6294820717132
14	H4	120	S2	RF	48.9043824701195
14	H4	120	S3	RF	135.159362549801
14	H4	120	S4	RF	145.119521912351
14	H4	120	S1	BB	845.238095238095
14	H4	120	S2	BB	2013.55311355311
14	H4	120	S3	BB	948.534798534798
14	H4	120	S4	BB	472.710622710623
14	H4	120	S1	FCU	9.90799716914366
14	H4	120	S2	FCU	649.186128803963
14	H4	120	S3	FCU	317.551309271054
14	H4	120	S4	FCU	47.2753007784855
14	H4	120	S1	ES	217.038539553753
14	H4	120	S2	ES	34.8884381338743
14	H4	120	S3	ES	159.499661933739
14	H4	120	S4	ES	255.578093306288
14	H4	120	S1	MG	56.3888888888889
14	H4	120	S2	MG	148.333333333333
14	H4	120	S3	MG	69.0277777777778

Continued on next page

Participant_ID	Seat_Height	Seat_Height_pct_PH	Stage	Muscle	Delta_RMS_pct
14	H4	120	S4	MG	223.611111111111
14	H4	120	S1	LM	205.438644438371
14	H4	120	S2	LM	-0.519267559442484
14	H4	120	S3	LM	-19.376878928669
14	H4	120	S4	LM	245.066958185296
15	H1	60	S1	RF	88.702201622248
15	H1	60	S2	RF	6.95249130938586
15	H1	60	S3	RF	54.1135573580533
15	H1	60	S4	RF	46.234067207416
15	H1	60	S1	BB	108.558558558559
15	H1	60	S2	BB	244.294294294294
15	H1	60	S3	BB	313.663663663664
15	H1	60	S4	BB	56.1561561561562
15	H1	60	S1	FCU	82.5757575757576
15	H1	60	S2	FCU	201.631701631702
15	H1	60	S3	FCU	-54.5454545454545
15	H1	60	S4	FCU	61.013986013986
15	H1	60	S1	ES	288.754534461911
15	H1	60	S2	ES	-40.5683192261185
15	H1	60	S3	ES	-45.6469165659008
15	H1	60	S4	ES	207.315598548972
15	H1	60	S1	MG	760.349854227405
15	H1	60	S2	MG	-4.81049562682216
15	H1	60	S3	MG	47.3760932944606
15	H1	60	S4	MG	280.393586005831
15	H1	60	S1	LM	-40.0278790478233
15	H1	60	S2	LM	-53.838730431053
15	H1	60	S3	LM	-36.2427621702766
15	H1	60	S4	LM	-65.3870898563157
15	H2	80	S1	RF	105.51445864156
15	H2	80	S2	RF	27.4377942165434
15	H2	80	S3	RF	129.320780094149
15	H2	80	S4	RF	177.942165433759
15	H2	80	S1	BB	134.235976789168
15	H2	80	S2	BB	373.307543520309
15	H2	80	S3	BB	243.133462282399
15	H2	80	S4	BB	122.630560928433
15	H2	80	S1	FCU	-30.5941845764855
15	H2	80	S2	FCU	-29.0613147914033
15	H2	80	S3	FCU	-37.7844500632111
15	H2	80	S4	FCU	25.3318584070797
15	H2	80	S1	ES	83.3433193529059
15	H2	80	S2	ES	-49.0113840623128
15	H2	80	S3	ES	-43.0796884361893
15	H2	80	S4	ES	263.571000599161
15	H2	80	S1	MG	232.633420822397
15	H2	80	S2	MG	146.106736657918
15	H2	80	S3	MG	172.965879265092
15	H2	80	S4	MG	251.793525809274
15	H2	80	S1	LM	-67.9643618051521
15	H2	80	S2	LM	-38.9889599070308
15	H2	80	S3	LM	-56.1107883013752
15	H2	80	S4	LM	-61.0885144295952
15	H3	100	S1	RF	53.734302709848
15	H3	100	S2	RF	20.8195637805684
15	H3	100	S3	RF	101.520158625248

Continued on next page

Participant_ID	Seat_Height	Seat_Height_pct_PH	Stage	Muscle	Delta_RMS_pct
15	H3	100	S4	RF	197.224058162591
15	H3	100	S1	BB	62.3024830699774
15	H3	100	S2	BB	362.7539503386
15	H3	100	S3	BB	176.975169300226
15	H3	100	S4	BB	81.2641083521445
15	H3	100	S1	FCU	128.608695652174
15	H3	100	S2	FCU	156.173913043478
15	H3	100	S3	FCU	243.391304347826
15	H3	100	S4	FCU	538.869565217391
15	H3	100	S1	ES	214.330874604847
15	H3	100	S2	ES	15.4899894625922
15	H3	100	S3	ES	-21.0748155953635
15	H3	100	S4	ES	527.502634351949
15	H3	100	S1	MG	238.228155339806
15	H3	100	S2	MG	96.2378640776699
15	H3	100	S3	MG	218.446601941748
15	H3	100	S4	MG	427.184466019417
15	H3	100	S1	LM	238.228155339806
15	H3	100	S2	LM	96.2378640776699
15	H3	100	S3	LM	218.446601941748
15	H3	100	S4	LM	427.184466019417
15	H4	120	S1	RF	57.0731707317073
15	H4	120	S2	RF	9.65853658536586
15	H4	120	S3	RF	58.390243902439
15	H4	120	S4	RF	125.560975609756
15	H4	120	S1	BB	136.904761904762
15	H4	120	S2	BB	739.68253968254
15	H4	120	S3	BB	457.936507936508
15	H4	120	S4	BB	218.253968253968
15	H4	120	S1	FCU	189.957264957265
15	H4	120	S2	FCU	569.65811965812
15	H4	120	S3	FCU	600.42735042735
15	H4	120	S4	FCU	296.794871794872
15	H4	120	S1	ES	117.115689381933
15	H4	120	S2	ES	40.8082408874802
15	H4	120	S3	ES	4.67511885895405
15	H4	120	S4	ES	220.126782884311
15	H4	120	S1	MG	91.2591687041565
15	H4	120	S2	MG	-13.3863080684597
15	H4	120	S3	MG	90.9535452322738
15	H4	120	S4	MG	141.870415647922
15	H4	120	S1	LM	-51.7746662324976
15	H4	120	S2	LM	11.1690003256268
15	H4	120	S3	LM	-19.8957994138717
15	H4	120	S4	LM	14.750895473787
16	H1	60	S1	RF	-0.216294160057674
16	H1	60	S2	RF	-55.2992069214131
16	H1	60	S3	RF	-10.8868060562365
16	H1	60	S4	RF	57.9668348954578
16	H1	60	S1	BB	102.765957446808
16	H1	60	S2	BB	1261.48936170213
16	H1	60	S3	BB	1050.21276595745
16	H1	60	S4	BB	105.31914893617
16	H1	60	S1	FCU	647.759932375317
16	H1	60	S2	FCU	412.256973795435
16	H1	60	S3	FCU	443.026204564666

Continued on next page

Participant_ID	Seat_Height	Seat_Height_pct_PH	Stage	Muscle	Delta_RMS_pct
16	H1	60	S4	FCU	776.838546069315
16	H1	60	S1	ES	168.147100424328
16	H1	60	S2	ES	-80.4809052333805
16	H1	60	S3	ES	-47.2418670438472
16	H1	60	S4	ES	240.678925035361
16	H1	60	S1	MG	-1.65217391304347
16	H1	60	S2	MG	-29.5652173913044
16	H1	60	S3	MG	-41.9130434782609
16	H1	60	S4	MG	111.478260869565
16	H1	60	S1	LM	-43.0147058823529
16	H1	60	S2	LM	39.9754901960784
16	H1	60	S3	LM	77.5245098039216
16	H1	60	S4	LM	-52.1078431372549
16	H2	80	S1	RF	95.9420289855072
16	H2	80	S2	RF	5.50724637681159
16	H2	80	S3	RF	167.391304347826
16	H2	80	S4	RF	118.985507246377
16	H2	80	S1	BB	301.261829652997
16	H2	80	S2	BB	1736.27760252366
16	H2	80	S3	BB	1179.81072555205
16	H2	80	S4	BB	60.5678233438486
16	H2	80	S1	FCU	1290.35916824197
16	H2	80	S2	FCU	1006.04914933837
16	H2	80	S3	FCU	630.812854442344
16	H2	80	S4	FCU	107.183364839319
16	H2	80	S1	ES	214.673913043478
16	H2	80	S2	ES	-62.0652173913044
16	H2	80	S3	ES	-32.0652173913044
16	H2	80	S4	ES	384.565217391304
16	H2	80	S1	MG	61.7151607963247
16	H2	80	S2	MG	-0.153139356814712
16	H2	80	S3	MG	102.909647779479
16	H2	80	S4	MG	276.87595712098
16	H2	80	S1	LM	-52.0156438026474
16	H2	80	S2	LM	81.2876052948255
16	H2	80	S3	LM	103.369434416366
16	H2	80	S4	LM	-69.3140794223827
16	H3	100	S1	RF	61.0687022900764
16	H3	100	S2	RF	-4.73282442748091
16	H3	100	S3	RF	122.290076335878
16	H3	100	S4	RF	171.75572519084
16	H3	100	S1	BB	49.1349480968858
16	H3	100	S2	BB	1586.15916955017
16	H3	100	S3	BB	279.584775086505
16	H3	100	S4	BB	130.103806228374
16	H3	100	S1	FCU	106.907894736842
16	H3	100	S2	FCU	647.861842105263
16	H3	100	S3	FCU	252.796052631579
16	H3	100	S4	FCU	36.8421052631579
16	H3	100	S1	ES	398.459845984598
16	H3	100	S2	ES	-12.6512651265126
16	H3	100	S3	ES	-54.6204620462046
16	H3	100	S4	ES	347.414741474147
16	H3	100	S1	MG	-1.88916876574308
16	H3	100	S2	MG	-6.80100755667506
16	H3	100	S3	MG	52.6448362720403

Continued on next page

Participant_ID	Seat_Height	Seat_Height_pct_PH	Stage	Muscle	Delta_RMS_pct
16	H3	100	S4	MG	83.6272040302267
16	H3	100	S1	LM	-2.44150559511699
16	H3	100	S2	LM	102.288911495422
16	H3	100	S3	LM	119.328585961343
16	H3	100	S4	LM	-44.2522889114954
16	H4	120	S1	RF	95.114006514658
16	H4	120	S2	RF	-9.28338762214983
16	H4	120	S3	RF	161.563517915309
16	H4	120	S4	RF	184.85342019544
16	H4	120	S1	BB	232.806324110672
16	H4	120	S2	BB	1320.55335968379
16	H4	120	S3	BB	825.296442687747
16	H4	120	S4	BB	192.490118577075
16	H4	120	S1	FCU	307.299270072993
16	H4	120	S2	FCU	631.386861313869
16	H4	120	S3	FCU	270.21897810219
16	H4	120	S4	FCU	164.817518248175
16	H4	120	S1	ES	343.267651888342
16	H4	120	S2	ES	97.0443349753695
16	H4	120	S3	ES	28.6535303776683
16	H4	120	S4	ES	249.507389162562
16	H4	120	S1	MG	148.673946957878
16	H4	120	S2	MG	23.4009360374415
16	H4	120	S3	MG	181.435257410296
16	H4	120	S4	MG	443.057722308892
16	H4	120	S1	LM	85.4545454545454
16	H4	120	S2	LM	341.818181818182
16	H4	120	S3	LM	534.659090909091
16	H4	120	S4	LM	168.295454545455
17	H1	60	S1	RF	333.599202392822
17	H1	60	S2	RF	-44.0677966101695
17	H1	60	S3	RF	-44.0677966101695
17	H1	60	S4	RF	47.357926221336
17	H1	60	S1	BB	76.9230769230769
17	H1	60	S2	BB	251.282051282051
17	H1	60	S3	BB	422.527472527473
17	H1	60	S4	BB	131.318681318681
17	H1	60	S1	FCU	-20.5365126676602
17	H1	60	S2	FCU	195.64828614009
17	H1	60	S3	FCU	209.329359165425
17	H1	60	S4	FCU	236.45305514158
17	H1	60	S1	ES	150.659501709819
17	H1	60	S2	ES	-63.409868099658
17	H1	60	S3	ES	-60.3321934538349
17	H1	60	S4	ES	442.745481191988
17	H1	60	S1	MG	209.547738693467
17	H1	60	S2	MG	-17.7554438860972
17	H1	60	S3	MG	4.35510887772196
17	H1	60	S4	MG	271.859296482412
17	H1	60	S1	LM	-34.1644562334218
17	H1	60	S2	LM	24.2970822281167
17	H1	60	S3	LM	15.8090185676392
17	H1	60	S4	LM	0.90185676392572
17	H2	80	S1	RF	65.4104979811575
17	H2	80	S2	RF	-12.2476446837147
17	H2	80	S3	RF	57.6043068640646

Continued on next page

Participant_ID	Seat_Height	Seat_Height_pct_PH	Stage	Muscle	Delta_RMS_pct
17	H2	80	S4	RF	201.615074024226
17	H2	80	S1	BB	59.7727272727273
17	H2	80	S2	BB	248.863636363636
17	H2	80	S3	BB	827.272727272727
17	H2	80	S4	BB	126.363636363636
17	H2	80	S1	FCU	601.052631578947
17	H2	80	S2	FCU	1652.48120300752
17	H2	80	S3	FCU	1175.03759398496
17	H2	80	S4	FCU	2159.84962406015
17	H2	80	S1	ES	463.310185185185
17	H2	80	S2	ES	193.981481481481
17	H2	80	S3	ES	238.773148148148
17	H2	80	S4	ES	986.921296296296
17	H2	80	S1	MG	65.5913978494624
17	H2	80	S2	MG	7.8853046594982
17	H2	80	S3	MG	10.3942652329749
17	H2	80	S4	MG	190.322580645161
17	H2	80	S1	LM	-50
17	H2	80	S2	LM	143.422404933196
17	H2	80	S3	LM	38.1294964028777
17	H2	80	S4	LM	3.13463514902363
17	H3	100	S1	RF	83.1592689295039
17	H3	100	S2	RF	46.7362924281984
17	H3	100	S3	RF	100.130548302872
17	H3	100	S4	RF	192.42819843342
17	H3	100	S1	BB	33.3836858006042
17	H3	100	S2	BB	22.4068479355488
17	H3	100	S3	BB	49.8489425981873
17	H3	100	S4	BB	-7.09969788519637
17	H3	100	S1	FCU	215.137614678899
17	H3	100	S2	FCU	378.047182175622
17	H3	100	S3	FCU	92.3328964613368
17	H3	100	S4	FCU	235.058977719528
17	H3	100	S1	ES	94.2403628117914
17	H3	100	S2	ES	127.800453514739
17	H3	100	S3	ES	1.63265306122449
17	H3	100	S4	ES	511.519274376417
17	H3	100	S1	MG	-48.7242394504416
17	H3	100	S2	MG	-65.9470068694799
17	H3	100	S3	MG	-23.3071638861629
17	H3	100	S4	MG	-52.5024533856722
17	H3	100	S1	LM	-10.9475620975161
17	H3	100	S2	LM	-4.4158233670653
17	H3	100	S3	LM	-36.1545538178473
17	H3	100	S4	LM	43.7902483900644
17	H4	120	S1	RF	32.5829383886256
17	H4	120	S2	RF	43.957345971564
17	H4	120	S3	RF	90.4028436018957
17	H4	120	S4	RF	157.464454976303
17	H4	120	S1	BB	-49.1092636579572
17	H4	120	S2	BB	248.396674584323
17	H4	120	S3	BB	56.2945368171021
17	H4	120	S4	BB	-45.8432304038005
17	H4	120	S1	FCU	835.204941044357
17	H4	120	S2	FCU	263.559797866367
17	H4	120	S3	FCU	966.367209432903

Continued on next page

Participant_ID	Seat_Height	Seat_Height_pct_PH	Stage	Muscle	Delta_RMS_pct
17	H4	120	S4	FCU	102.021336327906
17	H4	120	S1	ES	174.955804360636
17	H4	120	S2	ES	271.83264584561
17	H4	120	S3	ES	291.809074837949
17	H4	120	S4	ES	323.924572775486
17	H4	120	S1	MG	9.06630581867389
17	H4	120	S2	MG	81.7320703653586
17	H4	120	S3	MG	417.050067658999
17	H4	120	S4	MG	145.73748308525
17	H4	120	S1	LM	-42.2931314223905
17	H4	120	S2	LM	12.3039480800433
17	H4	120	S3	LM	200.919415900487
17	H4	120	S4	LM	2.19037317468903
18	H1	60	S1	RF	16.9896640826873
18	H1	60	S2	RF	-11.7571059431524
18	H1	60	S3	RF	-12.3385012919897
18	H1	60	S4	RF	105.038759689923
18	H1	60	S1	BB	17.4565086982603
18	H1	60	S2	BB	136.352729454109
18	H1	60	S3	BB	-0.539892021595701
18	H1	60	S4	BB	-10.9778044391122
18	H1	60	S1	FCU	-13.7844611528822
18	H1	60	S2	FCU	401.87969924812
18	H1	60	S3	FCU	64.6616541353383
18	H1	60	S4	FCU	-13.7844611528822
18	H1	60	S1	ES	96.4472917880023
18	H1	60	S2	ES	112.376237623762
18	H1	60	S3	ES	-24.3447874199185
18	H1	60	S4	ES	201.834595224228
18	H1	60	S1	MG	50.1607717041801
18	H1	60	S2	MG	78.8853161843516
18	H1	60	S3	MG	46.1950696677385
18	H1	60	S4	MG	182.315112540193
18	H1	60	S1	LM	-29.5758502101643
18	H1	60	S2	LM	-27.3786778754299
18	H1	60	S3	LM	-19.6025983951089
18	H1	60	S4	LM	-58.1773022544899
18	H2	80	S1	RF	62.9535864978903
18	H2	80	S2	RF	10.7172995780591
18	H2	80	S3	RF	87.5949367088608
18	H2	80	S4	RF	107.088607594937
18	H2	80	S1	BB	267.609254498715
18	H2	80	S2	BB	686.632390745501
18	H2	80	S3	BB	452.442159383033
18	H2	80	S4	BB	372.493573264781
18	H2	80	S1	FCU	134.622823984526
18	H2	80	S2	FCU	1105.99613152805
18	H2	80	S3	FCU	491.295938104449
18	H2	80	S4	FCU	84.7195357833656
18	H2	80	S1	ES	193.019480519481
18	H2	80	S2	ES	97.9978354978355
18	H2	80	S3	ES	60.7683982683983
18	H2	80	S4	ES	320.995670995671
18	H2	80	S1	MG	4.54545454545456
18	H2	80	S2	MG	39.0151515151515
18	H2	80	S3	MG	49.8484848484849

Continued on next page

Participant_ID	Seat_Height	Seat_Height_pct_PH	Stage	Muscle	Delta_RMS_pct
18	H2	80	S4	MG	70.4545454545455
18	H2	80	S1	LM	-32.137030995106
18	H2	80	S2	LM	-2.51223491027732
18	H2	80	S3	LM	-5.48123980424143
18	H2	80	S4	LM	-13.1484502446982
18	H3	100	S1	RF	19.3403298350825
18	H3	100	S2	RF	6.59670164917542
18	H3	100	S3	RF	-1.79910044977511
18	H3	100	S4	RF	27.136431784108
18	H3	100	S1	BB	158.258258258258
18	H3	100	S2	BB	710.21021021021
18	H3	100	S3	BB	388.588588588589
18	H3	100	S4	BB	74.1741741741742
18	H3	100	S1	FCU	186.756756756757
18	H3	100	S2	FCU	1552.97297297297
18	H3	100	S3	FCU	228.108108108108
18	H3	100	S4	FCU	83.5135135135135
18	H3	100	S1	ES	81.1244979919679
18	H3	100	S2	ES	115.037929495761
18	H3	100	S3	ES	-10.3078982597055
18	H3	100	S4	ES	165.640339134315
18	H3	100	S1	MG	10.1741521539872
18	H3	100	S2	MG	60.4032997250229
18	H3	100	S3	MG	13.1072410632447
18	H3	100	S4	MG	40.788267644363
18	H3	100	S1	LM	-36.4947310038824
18	H3	100	S2	LM	129.229062673322
18	H3	100	S3	LM	45.757071547421
18	H3	100	S4	LM	45.146977260122
18	H4	120	S1	RF	95.114006514658
18	H4	120	S2	RF	-9.28338762214983
18	H4	120	S3	RF	161.563517915309
18	H4	120	S4	RF	184.85342019544
18	H4	120	S1	BB	-49.1092636579572
18	H4	120	S2	BB	248.396674584323
18	H4	120	S3	BB	81.4726840855107
18	H4	120	S4	BB	-45.8432304038005
18	H4	120	S1	FCU	-31.8360471645143
18	H4	120	S2	FCU	263.559797866367
18	H4	120	S3	FCU	966.367209432903
18	H4	120	S4	FCU	102.021336327906
18	H4	120	S1	ES	174.955804360636
18	H4	120	S2	ES	271.83264584561
18	H4	120	S3	ES	291.809074837949
18	H4	120	S4	ES	323.924572775486
18	H4	120	S1	MG	9.06630581867389
18	H4	120	S2	MG	81.7320703653586
18	H4	120	S3	MG	417.050067658999
18	H4	120	S4	MG	145.73748308525
18	H4	120	S1	LM	-42.2931314223905
18	H4	120	S2	LM	12.3039480800433
18	H4	120	S3	LM	200.919415900487
18	H4	120	S4	LM	2.19037317468903
19	H1	60	S1	RF	164.488017429194
19	H1	60	S2	RF	39.760348583878
19	H1	60	S3	RF	141.176470588235

Continued on next page

Participant_ID	Seat_Height	Seat_Height_pct_PH	Stage	Muscle	Delta_RMS_pct
19	H1	60	S4	RF	232.897603485839
19	H1	60	S1	BB	153.779069767442
19	H1	60	S2	BB	1041.86046511628
19	H1	60	S3	BB	521.511627906977
19	H1	60	S4	BB	197.96511627907
19	H1	60	S1	FCU	503.645833333333
19	H1	60	S2	FCU	813.194444444444
19	H1	60	S3	FCU	2271.44097222222
19	H1	60	S4	FCU	486.284722222222
19	H1	60	S1	ES	359.64924787909
19	H1	60	S2	ES	-47.750766379126
19	H1	60	S3	ES	-53.9174449276396
19	H1	60	S4	ES	228.445141512797
19	H1	60	S1	MG	110.997732426304
19	H1	60	S2	MG	99.2063492063492
19	H1	60	S3	MG	62.6984126984127
19	H1	60	S4	MG	208.390022675737
19	H1	60	S1	LM	199.647978464565
19	H1	60	S2	LM	-25.5629756173319
19	H1	60	S3	LM	-1.38737899259718
19	H1	60	S4	LM	233.322979758762
19	H2	80	S1	RF	536.647173489279
19	H2	80	S2	RF	-21.8973359324237
19	H2	80	S3	RF	36.7771280051982
19	H2	80	S4	RF	83.8206627680312
19	H2	80	S1	BB	1049.6875
19	H2	80	S2	BB	702.8125
19	H2	80	S3	BB	3110
19	H2	80	S4	BB	365.3125
19	H2	80	S1	FCU	1104.17457305503
19	H2	80	S2	FCU	1546.29981024668
19	H2	80	S3	FCU	1403.79506641366
19	H2	80	S4	FCU	1263.56736242884
19	H2	80	S1	ES	402.555871095197
19	H2	80	S2	ES	-64.9956784788245
19	H2	80	S3	ES	-25.5340165452525
19	H2	80	S4	ES	221.805161131004
19	H2	80	S1	MG	164.437984496124
19	H2	80	S2	MG	74.6124031007752
19	H2	80	S3	MG	101.647286821705
19	H2	80	S4	MG	319.186046511628
19	H2	80	S1	LM	37.2454286193592
19	H2	80	S2	LM	-74.5747357825868
19	H2	80	S3	LM	-58.9867471900688
19	H2	80	S4	LM	97.1984566347928
19	H3	100	S1	RF	67.9755671902269
19	H3	100	S2	RF	51.5706806282722
19	H3	100	S3	RF	129.493891797557
19	H3	100	S4	RF	161.256544502618
19	H3	100	S1	BB	52.5806451612903
19	H3	100	S2	BB	1622.58064516129
19	H3	100	S3	BB	156.774193548387
19	H3	100	S4	BB	60.9677419354839
19	H3	100	S1	FCU	570.487106017192
19	H3	100	S2	FCU	2363.89684813754
19	H3	100	S3	FCU	717.191977077364

Continued on next page

Participant_ID	Seat_Height	Seat_Height_pct_PH	Stage	Muscle	Delta_RMS_pct
19	H3	100	S4	FCU	1313.18051575931
19	H3	100	S1	ES	275.091689426453
19	H3	100	S2	ES	-35.7237612173234
19	H3	100	S3	ES	-64.0889582520484
19	H3	100	S4	ES	317.557549746391
19	H3	100	S1	MG	102.574002574003
19	H3	100	S2	MG	197.168597168597
19	H3	100	S3	MG	79.021879021879
19	H3	100	S4	MG	180.566280566281
19	H3	100	S1	LM	108.440059736616
19	H3	100	S2	LM	-59.8497533601846
19	H3	100	S3	LM	-54.9305335565914
19	H3	100	S4	LM	126.066886907725
19	H4	120	S1	RF	47.8232618583496
19	H4	120	S2	RF	-0.584795321637426
19	H4	120	S3	RF	79.9870045484081
19	H4	120	S4	RF	90.2534113060429
19	H4	120	S1	BB	31.2380952380952
19	H4	120	S2	BB	628.190476190476
19	H4	120	S3	BB	136
19	H4	120	S4	BB	-14.0952380952381
19	H4	120	S1	FCU	557.530120481928
19	H4	120	S2	FCU	2785.84337349398
19	H4	120	S3	FCU	760.843373493976
19	H4	120	S4	FCU	819.578313253012
19	H4	120	S1	ES	380.729866639098
19	H4	120	S2	ES	-46.7486818980668
19	H4	120	S3	ES	-0.754677969606124
19	H4	120	S4	ES	280.771218856611
19	H4	120	S1	MG	210.068027210884
19	H4	120	S2	MG	240.680272108843
19	H4	120	S3	MG	382.721088435374
19	H4	120	S4	MG	988.571428571429
19	H4	120	S1	LM	78.7268031630235
19	H4	120	S2	LM	-64.6928831971032
19	H4	120	S3	LM	-62.7253121755105
19	H4	120	S4	LM	74.6345749354349
20	H1	60	S1	RF	218.587360594796
20	H1	60	S2	RF	116.976456009913
20	H1	60	S3	RF	-16.6047087980173
20	H1	60	S4	RF	246.592317224288
20	H1	60	S1	BB	256.413043478261
20	H1	60	S2	BB	107.934782608696
20	H1	60	S3	BB	-19.2391304347826
20	H1	60	S4	BB	151.739130434783
20	H1	60	S1	FCU	155.467720685112
20	H1	60	S2	FCU	1391.69960474308
20	H1	60	S3	FCU	52.5691699604743
20	H1	60	S4	FCU	977.997364953887
20	H1	60	S1	ES	452.385406922357
20	H1	60	S2	ES	-13.0028063610851
20	H1	60	S3	ES	-78.6716557530402
20	H1	60	S4	ES	718.615528531338
20	H1	60	S1	MG	65.3399668325041
20	H1	60	S2	MG	-14.2620232172471
20	H1	60	S3	MG	103.758982863461

Continued on next page

Participant_ID	Seat_Height	Seat_Height_pct_PH	Stage	Muscle	Delta_RMS_pct
20	H1	60	S4	MG	61.9679380873411
20	H1	60	S1	LM	30.2414718282867
20	H1	60	S2	LM	-17.1330011498658
20	H1	60	S3	LM	-32.6178612495209
20	H1	60	S4	LM	-8.27903411268685
20	H2	80	S1	RF	318.636995827538
20	H2	80	S2	RF	36.5785813630042
20	H2	80	S3	RF	251.112656467316
20	H2	80	S4	RF	83.1710709318498
20	H2	80	S1	BB	747.214076246334
20	H2	80	S2	BB	427.859237536657
20	H2	80	S3	BB	486.217008797654
20	H2	80	S4	BB	282.697947214076
20	H2	80	S1	FCU	343.872549019608
20	H2	80	S2	FCU	1004.04411764706
20	H2	80	S3	FCU	315.073529411765
20	H2	80	S4	FCU	597.058823529412
20	H2	80	S1	ES	261.642228739003
20	H2	80	S2	ES	-46.0410557184751
20	H2	80	S3	ES	-32.6686217008798
20	H2	80	S4	ES	427.741935483871
20	H2	80	S1	MG	114.180929095355
20	H2	80	S2	MG	-24.2053789731051
20	H2	80	S3	MG	-8.55745721271393
20	H2	80	S4	MG	121.18989405053
20	H2	80	S1	LM	-51.8282988871224
20	H2	80	S2	LM	-63.1614808085396
20	H2	80	S3	LM	-33.4317510788099
20	H2	80	S4	LM	-48.7394957983193
20	H3	100	S1	RF	5.46282245827011
20	H3	100	S2	RF	46.4339908952959
20	H3	100	S3	RF	20.0303490136571
20	H3	100	S4	RF	120.333839150228
20	H3	100	S1	BB	129.323308270677
20	H3	100	S2	BB	667.919799498747
20	H3	100	S3	BB	125.062656641604
20	H3	100	S4	BB	52.3809523809524
20	H3	100	S1	FCU	260
20	H3	100	S2	FCU	4608.63636363636
20	H3	100	S3	FCU	277.727272727273
20	H3	100	S4	FCU	1147.27272727273
20	H3	100	S1	ES	847.832167832168
20	H3	100	S2	ES	359.58041958042
20	H3	100	S3	ES	16.9230769230769
20	H3	100	S4	ES	996.643356643357
20	H3	100	S1	MG	13.2879045996593
20	H3	100	S2	MG	5.62180579216354
20	H3	100	S3	MG	31.5161839863714
20	H3	100	S4	MG	303.747870528109
20	H3	100	S1	LM	31.8478260869565
20	H3	100	S2	LM	102.391304347826
20	H3	100	S3	LM	124.782608695652
20	H3	100	S4	LM	101.195652173913
20	H4	120	S1	RF	162.882882882883
20	H4	120	S2	RF	104.504504504504
20	H4	120	S3	RF	38.5585585585586

Continued on next page

Participant_ID	Seat_Height	Seat_Height_pct_PH	Stage	Muscle	Delta_RMS_pct
20	H4	120	S4	RF	119.81981981982
20	H4	120	S1	BB	-28.7746170678337
20	H4	120	S2	BB	165.317286652079
20	H4	120	S3	BB	30.6345733041575
20	H4	120	S4	BB	-19.3654266958425
20	H4	120	S1	FCU	21.9759926131117
20	H4	120	S2	FCU	834.072022160665
20	H4	120	S3	FCU	39.9815327793167
20	H4	120	S4	FCU	3.69344413665744
20	H4	120	S1	ES	272.078593588418
20	H4	120	S2	ES	175.853154084798
20	H4	120	S3	ES	26.5770423991727
20	H4	120	S4	ES	358.066184074457
20	H4	120	S1	MG	68.0602006688963
20	H4	120	S2	MG	42.3076923076923
20	H4	120	S3	MG	93.8127090301003
20	H4	120	S4	MG	136.28762541806
20	H4	120	S1	LM	172.83105022831
20	H4	120	S2	LM	215.639269406393
20	H4	120	S3	LM	65.1826484018265
20	H4	120	S4	LM	112.899543378995
21	H1	60	S1	RF	293.027210884354
21	H1	60	S2	RF	267.34693877551
21	H1	60	S3	RF	54.7619047619048
21	H1	60	S4	RF	261.394557823129
21	H1	60	S1	BB	129.154518950437
21	H1	60	S2	BB	600
21	H1	60	S3	BB	170.553935860058
21	H1	60	S4	BB	32.6530612244898
21	H1	60	S1	FCU	69.3328278999242
21	H1	60	S2	FCU	396.057619408643
21	H1	60	S3	FCU	34.8749052312358
21	H1	60	S4	FCU	-29.0371493555724
21	H1	60	S1	ES	1942.92379471229
21	H1	60	S2	ES	104.199066874028
21	H1	60	S3	ES	2.48833592534992
21	H1	60	S4	ES	2253.03265940902
21	H1	60	S1	MG	22.2972972972973
21	H1	60	S2	MG	9.59459459459458
21	H1	60	S3	MG	-18.1081081081081
21	H1	60	S4	MG	47.7027027027027
21	H1	60	S1	LM	-90.3862091828278
21	H1	60	S2	LM	59.9204375932372
21	H1	60	S3	LM	-23.1228244654401
21	H1	60	S4	LM	-84.1703961544837
21	H2	80	S1	RF	293.027210884354
21	H2	80	S2	RF	267.34693877551
21	H2	80	S3	RF	54.7619047619048
21	H2	80	S4	RF	261.394557823129
21	H2	80	S1	BB	4.66045272969375
21	H2	80	S2	BB	238.082556591212
21	H2	80	S3	BB	124.234354194408
21	H2	80	S4	BB	-39.4141145139814
21	H2	80	S1	FCU	47.9651162790698
21	H2	80	S2	FCU	1373.54651162791
21	H2	80	S3	FCU	3500.87209302326

Continued on next page

Participant_ID	Seat_Height	Seat_Height_pct_PH	Stage	Muscle	Delta_RMS_pct
21	H2	80	S4	FCU	15.4069767441861
21	H2	80	S1	ES	1105.78512396694
21	H2	80	S2	ES	118.842975206612
21	H2	80	S3	ES	122.479338842975
21	H2	80	S4	ES	1897.52066115702
21	H2	80	S1	MG	13.452188006483
21	H2	80	S2	MG	-9.56239870340356
21	H2	80	S3	MG	7.61750405186385
21	H2	80	S4	MG	43.2739059967585
21	H2	80	S1	LM	9.79789103690684
21	H2	80	S2	LM	288.664323374341
21	H2	80	S3	LM	390.158172231986
21	H2	80	S4	LM	-45.9138840070299
21	H3	100	S1	RF	87.9611650485437
21	H3	100	S2	RF	112.427184466019
21	H3	100	S3	RF	79.8058252427184
21	H3	100	S4	RF	131.26213592233
21	H3	100	S1	BB	68.6346863468635
21	H3	100	S2	BB	403.690036900369
21	H3	100	S3	BB	147.60147601476
21	H3	100	S4	BB	78.9667896678967
21	H3	100	S1	FCU	59.5520421607378
21	H3	100	S2	FCU	1442.9512516469
21	H3	100	S3	FCU	454.940711462451
21	H3	100	S4	FCU	58.6297760210804
21	H3	100	S1	ES	439.767649687221
21	H3	100	S2	ES	95.0848972296693
21	H3	100	S3	ES	71.4924039320822
21	H3	100	S4	ES	804.37890974084
21	H3	100	S1	MG	32.780847145488
21	H3	100	S2	MG	62.0626151012891
21	H3	100	S3	MG	63.3517495395948
21	H3	100	S4	MG	102.025782688766
21	H3	100	S1	LM	-74.1874408330704
21	H3	100	S2	LM	251.088671505207
21	H3	100	S3	LM	-26.3805616913853
21	H3	100	S4	LM	-47.7437677500789
21	H4	120	S1	RF	140.963855421687
21	H4	120	S2	RF	80.421686746988
21	H4	120	S3	RF	58.8855421686747
21	H4	120	S4	RF	207.379518072289
21	H4	120	S1	BB	46.2829736211031
21	H4	120	S2	BB	660.191846522782
21	H4	120	S3	BB	76.0191846522782
21	H4	120	S4	BB	-13.4292565947242
21	H4	120	S1	FCU	-38.3458646616541
21	H4	120	S2	FCU	706.390977443609
21	H4	120	S3	FCU	6.34398496240601
21	H4	120	S4	FCU	12.0770676691729
21	H4	120	S1	ES	606.785714285714
21	H4	120	S2	ES	521.904761904762
21	H4	120	S3	ES	48.2142857142857
21	H4	120	S4	ES	1370.71428571429
21	H4	120	S1	MG	9.40054495912806
21	H4	120	S2	MG	16.2125340599455
21	H4	120	S3	MG	-8.71934604904632

Continued on next page

Participant_ID	Seat_Height	Seat_Height_pct_PH	Stage	Muscle	Delta_RMS_pct
21	H4	120	S4	MG	45.0953678474115
21	H4	120	S1	LM	-74.4536940686785
21	H4	120	S2	LM	129.656607700312
21	H4	120	S3	LM	75.2861602497399
21	H4	120	S4	LM	-73.3610822060354
22	H1	60	S1	RF	205.148005148005
22	H1	60	S2	RF	21.3642213642214
22	H1	60	S3	RF	96.6537966537966
22	H1	60	S4	RF	234.749034749035
22	H1	60	S1	BB	3514.59627329193
22	H1	60	S2	BB	3360.24844720497
22	H1	60	S3	BB	1231.98757763975
22	H1	60	S4	BB	1409.31677018633
22	H1	60	S1	FCU	1863.99132321041
22	H1	60	S2	FCU	1564.20824295011
22	H1	60	S3	FCU	552.494577006508
22	H1	60	S4	FCU	801.518438177874
22	H1	60	S1	ES	552.658662092624
22	H1	60	S2	ES	-22.8987993138937
22	H1	60	S3	ES	-46.1406518010292
22	H1	60	S4	ES	553.087478559177
22	H1	60	S1	MG	1027.52562225476
22	H1	60	S2	MG	784.040995607613
22	H1	60	S3	MG	133.235724743777
22	H1	60	S4	MG	653.440702781845
22	H1	60	S1	LM	60.3867403314917
22	H1	60	S2	LM	124.033149171271
22	H1	60	S3	LM	74.8618784530387
22	H1	60	S4	LM	22.9834254143646
22	H2	80	S1	RF	62.0222929936306
22	H2	80	S2	RF	10.031847133758
22	H2	80	S3	RF	21.7356687898089
22	H2	80	S4	RF	102.468152866242
22	H2	80	S1	BB	1328.41726618705
22	H2	80	S2	BB	4166.54676258993
22	H2	80	S3	BB	2565.46762589928
22	H2	80	S4	BB	1692.44604316547
22	H2	80	S1	FCU	1132.98755186722
22	H2	80	S2	FCU	1030.91286307054
22	H2	80	S3	FCU	646.887966804979
22	H2	80	S4	FCU	492.116182572614
22	H2	80	S1	ES	114.916067146283
22	H2	80	S2	ES	-14.1486810551559
22	H2	80	S3	ES	16.978417266187
22	H2	80	S4	ES	256.306954436451
22	H2	80	S1	MG	674.406779661017
22	H2	80	S2	MG	406.949152542373
22	H2	80	S3	MG	211.525423728814
22	H2	80	S4	MG	687.966101694915
22	H2	80	S1	LM	-37.1468926553672
22	H2	80	S2	LM	20.4802259887006
22	H2	80	S3	LM	52.1751412429378
22	H2	80	S4	LM	-6.89265536723163
22	H3	100	S1	RF	93.8574938574938
22	H3	100	S2	RF	38.4520884520884
22	H3	100	S3	RF	168.550368550368

Continued on next page

Participant_ID	Seat_Height	Seat_Height_pct_PH	Stage	Muscle	Delta_RMS_pct
22	H3	100	S4	RF	246.928746928747
22	H3	100	S1	BB	284.060552092609
22	H3	100	S2	BB	644.612644701692
22	H3	100	S3	BB	55.2092609082814
22	H3	100	S4	BB	220.926090828139
22	H3	100	S1	FCU	828.308823529412
22	H3	100	S2	FCU	1270.03676470588
22	H3	100	S3	FCU	256.985294117647
22	H3	100	S4	FCU	345.220588235294
22	H3	100	S1	ES	39.7820163487739
22	H3	100	S2	ES	-1.04450499545868
22	H3	100	S3	ES	-51.7711171662125
22	H3	100	S4	ES	98.8646684831971
22	H3	100	S1	MG	1034.88773747841
22	H3	100	S2	MG	244.041450777202
22	H3	100	S3	MG	335.751295336788
22	H3	100	S4	MG	734.024179620035
22	H3	100	S1	LM	-63.8736263736264
22	H3	100	S2	LM	35.679945054945
22	H3	100	S3	LM	-29.4299450549451
22	H3	100	S4	LM	-31.1126373626374
22	H4	120	S1	RF	160.718870346598
22	H4	120	S2	RF	95.6354300385109
22	H4	120	S3	RF	259.435173299101
22	H4	120	S4	RF	197.560975609756
22	H4	120	S1	BB	-14.0773158278629
22	H4	120	S2	BB	467.541940189643
22	H4	120	S3	BB	177.607585703866
22	H4	120	S4	BB	-56.6739606126915
22	H4	120	S1	FCU	818.961038961039
22	H4	120	S2	FCU	689.480519480519
22	H4	120	S3	FCU	212.077922077922
22	H4	120	S4	FCU	-5.58441558441559
22	H4	120	S1	ES	213.075060532688
22	H4	120	S2	ES	89.4269572235674
22	H4	120	S3	ES	90.2340597255851
22	H4	120	S4	ES	431.315577078289
22	H4	120	S1	MG	515.800415800416
22	H4	120	S2	MG	175.467775467776
22	H4	120	S3	MG	386.486486486487
22	H4	120	S4	MG	482.952182952183
22	H4	120	S1	LM	-62.5614150016377
22	H4	120	S2	LM	33.2459875532263
22	H4	120	S3	LM	7.10776285620701
22	H4	120	S4	LM	-24.8935473304946
23	H1	60	S1	RF	286.252945797329
23	H1	60	S2	RF	285.624509033779
23	H1	60	S3	RF	459.62293794187
23	H1	60	S4	RF	274.39120188531
23	H1	60	S1	BB	903.684210526316
23	H1	60	S2	BB	524.736842105263
23	H1	60	S3	BB	295.789473684211
23	H1	60	S4	BB	521.842105263158
23	H1	60	S1	FCU	334.160090191657
23	H1	60	S2	FCU	466.178128523112
23	H1	60	S3	FCU	141.600901916573

Continued on next page

Participant_ID	Seat_Height	Seat_Height_pct_PH	Stage	Muscle	Delta_RMS_pct
23	H1	60	S4	FCU	228.410372040586
23	H1	60	S1	ES	152.417794970987
23	H1	60	S2	ES	59.284332688588
23	H1	60	S3	ES	7.05996131528047
23	H1	60	S4	ES	154.061895551257
23	H1	60	S1	MG	0.58377116170462
23	H1	60	S2	MG	87.0402802101576
23	H1	60	S3	MG	-3.91126678342089
23	H1	60	S4	MG	50.1459427904261
23	H1	60	S1	LM	274.074074074074
23	H1	60	S2	LM	133.333333333333
23	H1	60	S3	LM	140.17094017094
23	H1	60	S4	LM	324.643874643875
23	H2	80	S1	RF	121.174764321972
23	H2	80	S2	RF	127.701232777375
23	H2	80	S3	RF	199.637418419144
23	H2	80	S4	RF	482.088469905729
23	H2	80	S1	BB	265.373134328358
23	H2	80	S2	BB	863.582089552239
23	H2	80	S3	BB	610.44776119403
23	H2	80	S4	BB	500.597014925373
23	H2	80	S1	FCU	140.729783037475
23	H2	80	S2	FCU	439.151873767258
23	H2	80	S3	FCU	203.353057199211
23	H2	80	S4	FCU	538.067061143984
23	H2	80	S1	ES	174.11907654921
23	H2	80	S2	ES	154.921020656136
23	H2	80	S3	ES	136.81652490887
23	H2	80	S4	ES	189.307411907655
23	H2	80	S1	MG	-38.5705483672212
23	H2	80	S2	MG	32.5323475046211
23	H2	80	S3	MG	-20.3327171903882
23	H2	80	S4	MG	12.5077017868146
23	H2	80	S1	LM	141.245136186771
23	H2	80	S2	LM	95.5252918287938
23	H2	80	S3	LM	136.381322957199
23	H2	80	S4	LM	212.84046692607
23	H3	100	S1	RF	116.14010989011
23	H3	100	S2	RF	103.159340659341
23	H3	100	S3	RF	313.942307692308
23	H3	100	S4	RF	149.862637362637
23	H3	100	S1	BB	239.855072463768
23	H3	100	S2	BB	719.927536231884
23	H3	100	S3	BB	638.04347826087
23	H3	100	S4	BB	601.811594202899
23	H3	100	S1	FCU	574.621212121212
23	H3	100	S2	FCU	1846.59090909091
23	H3	100	S3	FCU	898.863636363636
23	H3	100	S4	FCU	1047.34848484848
23	H3	100	S1	ES	319.460227272727
23	H3	100	S2	ES	138.210227272727
23	H3	100	S3	ES	125
23	H3	100	S4	ES	167.755681818182
23	H3	100	S1	MG	-1.90294957183634
23	H3	100	S2	MG	22.2645099904853
23	H3	100	S3	MG	46.4319695528069

Continued on next page

Participant_ID	Seat_Height	Seat_Height_pct_PH	Stage	Muscle	Delta_RMS_pct
23	H3	100	S4	MG	59.7526165556613
23	H3	100	S1	LM	151.523947750363
23	H3	100	S2	LM	112.917271407837
23	H3	100	S3	LM	203.338171262699
23	H3	100	S4	LM	269.66618287373
23	H4	120	S1	RF	294.215795328142
23	H4	120	S2	RF	89.432703003337
23	H4	120	S3	RF	85.4282536151279
23	H4	120	S4	RF	365.294771968854
23	H4	120	S1	BB	119.064748201439
23	H4	120	S2	BB	766.546762589928
23	H4	120	S3	BB	177.697841726619
23	H4	120	S4	BB	54.3165467625899
23	H4	120	S1	FCU	21.1029411764706
23	H4	120	S2	FCU	356.25
23	H4	120	S3	FCU	386.544117647059
23	H4	120	S4	FCU	-12.7205882352941
23	H4	120	S1	ES	29.6476306196841
23	H4	120	S2	ES	74.2405832320778
23	H4	120	S3	ES	130.498177399757
23	H4	120	S4	ES	164.15552855407
23	H4	120	S1	MG	50.4065040650407
23	H4	120	S2	MG	218.563685636856
23	H4	120	S3	MG	142.818428184282
23	H4	120	S4	MG	157.317073170732
23	H4	120	S1	LM	-2.81837160751565
23	H4	120	S2	LM	0.417536534446755
23	H4	120	S3	LM	113.883089770355
23	H4	120	S4	LM	42.901878914405
24	H1	60	S1	RF	273.502871205907
24	H1	60	S2	RF	-60.9515996718622
24	H1	60	S3	RF	-67.3502871205907
24	H1	60	S4	RF	225.102543068089
24	H1	60	S1	BB	417.783505154639
24	H1	60	S2	BB	1281.18556701031
24	H1	60	S3	BB	773.711340206186
24	H1	60	S4	BB	264.690721649484
24	H1	60	S1	FCU	1617.08333333333
24	H1	60	S2	FCU	933.125
24	H1	60	S3	FCU	579.166666666667
24	H1	60	S4	FCU	260.625
24	H1	60	S1	ES	-57.7338129496403
24	H1	60	S2	ES	-72.1622701838529
24	H1	60	S3	ES	-62.5899280575539
24	H1	60	S4	ES	-7.414068745004
24	H1	60	S1	MG	-11.7570215545395
24	H1	60	S2	MG	46.2442847811888
24	H1	60	S3	MG	-21.1626387981711
24	H1	60	S4	MG	16.5904637491835
24	H1	60	S1	LM	133.588621444201
24	H1	60	S2	LM	-7.54923413566741
24	H1	60	S3	LM	110.175054704595
24	H1	60	S4	LM	291.028446389497
24	H2	80	S1	RF	483.009708737864
24	H2	80	S2	RF	-14.2394822006473
24	H2	80	S3	RF	-33.0097087378641

Continued on next page

Participant_ID	Seat_Height	Seat_Height_pct_PH	Stage	Muscle	Delta_RMS_pct
24	H2	80	S4	RF	438.511326860842
24	H2	80	S1	BB	187.290167865707
24	H2	80	S2	BB	784.652278177458
24	H2	80	S3	BB	472.182254196643
24	H2	80	S4	BB	51.0791366906475
24	H2	80	S1	FCU	1453.31491712707
24	H2	80	S2	FCU	1354.97237569061
24	H2	80	S3	FCU	748.066298342541
24	H2	80	S4	FCU	1406.0773480663
24	H2	80	S1	ES	29.505076142132
24	H2	80	S2	ES	1.07868020304569
24	H2	80	S3	ES	32.6776649746193
24	H2	80	S4	ES	25.1269035532995
24	H2	80	S1	MG	-36.7066895368782
24	H2	80	S2	MG	241.680960548885
24	H2	80	S3	MG	42.1955403087478
24	H2	80	S4	MG	-4.45969125214408
24	H2	80	S1	LM	226.832151300236
24	H2	80	S2	LM	88.4160756501182
24	H2	80	S3	LM	186.643026004728
24	H2	80	S4	LM	265.130023640662
24	H3	100	S1	RF	242.036124794745
24	H3	100	S2	RF	-1.3136288998358
24	H3	100	S3	RF	-11.8226600985222
24	H3	100	S4	RF	289.490968801314
24	H3	100	S1	BB	162.924281984334
24	H3	100	S2	BB	790.339425587467
24	H3	100	S3	BB	936.553524804178
24	H3	100	S4	BB	25.3263707571802
24	H3	100	S1	FCU	1034.36426116838
24	H3	100	S2	FCU	2341.23711340206
24	H3	100	S3	FCU	1723.71134020619
24	H3	100	S4	FCU	186.59793814433
24	H3	100	S1	ES	79.8174442190669
24	H3	100	S2	ES	-7.60649087221095
24	H3	100	S3	ES	126.470588235294
24	H3	100	S4	ES	89.8580121703854
24	H3	100	S1	MG	25.3521126760564
24	H3	100	S2	MG	332.696177062374
24	H3	100	S3	MG	260.160965794769
24	H3	100	S4	MG	24.8490945674044
24	H3	100	S1	LM	198.520345252774
24	H3	100	S2	LM	23.5511713933416
24	H3	100	S3	LM	238.840937114673
24	H3	100	S4	LM	309.617755856967
24	H4	120	S1	RF	388.079470198676
24	H4	120	S2	RF	84.271523178808
24	H4	120	S3	RF	70.6953642384106
24	H4	120	S4	RF	388.576158940397
24	H4	120	S1	BB	216.103896103896
24	H4	120	S2	BB	2687.79220779221
24	H4	120	S3	BB	438.701298701299
24	H4	120	S4	BB	119.48051948052
24	H4	120	S1	FCU	485.772357723577
24	H4	120	S2	FCU	4780.48780487805
24	H4	120	S3	FCU	1286.17886178862

Continued on next page

Participant_ID	Seat_Height	Seat_Height_pct_PH	Stage	Muscle	Delta_RMS_pct
24	H4	120	S4	FCU	293.49593495935
24	H4	120	S1	ES	193.371757925072
24	H4	120	S2	ES	67.1469740634006
24	H4	120	S3	ES	327.233429394813
24	H4	120	S4	ES	156.916426512968
24	H4	120	S1	MG	-38.5321100917431
24	H4	120	S2	MG	250.688073394495
24	H4	120	S3	MG	-30.8103975535168
24	H4	120	S4	MG	13.914373088685
24	H4	120	S1	LM	341.28
24	H4	120	S2	LM	561.28
24	H4	120	S3	LM	450.72
24	H4	120	S4	LM	314.24
25	H1	60	S1	RF	433.052147239264
25	H1	60	S2	RF	-59.8926380368098
25	H1	60	S3	RF	27.4539877300614
25	H1	60	S4	RF	398.542944785276
25	H1	60	S1	BB	213.945578231293
25	H1	60	S2	BB	978.91156462585
25	H1	60	S3	BB	211.56462585034
25	H1	60	S4	BB	115.646258503401
25	H1	60	S1	FCU	258.111380145278
25	H1	60	S2	FCU	746.973365617433
25	H1	60	S3	FCU	403.63196125908
25	H1	60	S4	FCU	829.055690072639
25	H1	60	S1	ES	183.761840324763
25	H1	60	S2	ES	62.3815967523681
25	H1	60	S3	ES	111.502029769959
25	H1	60	S4	ES	297.699594046008
25	H1	60	S1	MG	-21.6401734104046
25	H1	60	S2	MG	36.6329479768786
25	H1	60	S3	MG	-73.1575144508671
25	H1	60	S4	MG	51.3728323699422
25	H1	60	S1	LM	403.92523364486
25	H1	60	S2	LM	340.373831775701
25	H1	60	S3	LM	354.018691588785
25	H1	60	S4	LM	1031.58878504673
25	H2	80	S1	RF	257.391826923077
25	H2	80	S2	RF	-69.2908653846154
25	H2	80	S3	RF	28.6057692307692
25	H2	80	S4	RF	125
25	H2	80	S1	BB	136.585365853658
25	H2	80	S2	BB	1108.53658536585
25	H2	80	S3	BB	414.227642276423
25	H2	80	S4	BB	163.821138211382
25	H2	80	S1	FCU	514.626865671642
25	H2	80	S2	FCU	1036.11940298508
25	H2	80	S3	FCU	370.149253731343
25	H2	80	S4	FCU	608.65671641791
25	H2	80	S1	ES	128.09667673716
25	H2	80	S2	ES	111.78247734139
25	H2	80	S3	ES	203.323262839879
25	H2	80	S4	ES	249.093655589124
25	H2	80	S1	MG	-25.0821018062397
25	H2	80	S2	MG	53.0788177339901
25	H2	80	S3	MG	163.546798029557

Continued on next page

Participant_ID	Seat_Height	Seat_Height_pct_PH	Stage	Muscle	Delta_RMS_pct
25	H2	80	S4	MG	78.6945812807882
25	H2	80	S1	LM	341.050583657588
25	H2	80	S2	LM	128.015564202335
25	H2	80	S3	LM	400.583657587549
25	H2	80	S4	LM	779.961089494163
25	H3	100	S1	RF	214.636449480642
25	H3	100	S2	RF	12.1813031161473
25	H3	100	S3	RF	5.38243626062323
25	H3	100	S4	RF	159.962228517469
25	H3	100	S1	BB	193.890675241158
25	H3	100	S2	BB	785.852090032154
25	H3	100	S3	BB	231.511254019293
25	H3	100	S4	BB	79.7427652733119
25	H3	100	S1	FCU	306.811145510836
25	H3	100	S2	FCU	746.130030959752
25	H3	100	S3	FCU	354.179566563468
25	H3	100	S4	FCU	68.1114551083591
25	H3	100	S1	ES	81.7708333333333
25	H3	100	S2	ES	73.2291666666667
25	H3	100	S3	ES	121.041666666667
25	H3	100	S4	ES	100.625
25	H3	100	S1	MG	49.4640122511486
25	H3	100	S2	MG	65.5946911689637
25	H3	100	S3	MG	4.79836651352732
25	H3	100	S4	MG	114.803471158755
25	H3	100	S1	LM	230.988023952096
25	H3	100	S2	LM	188.622754491018
25	H3	100	S3	LM	395.658682634731
25	H3	100	S4	LM	469.910179640719
25	H4	120	S1	RF	-54.7074777218132
25	H4	120	S2	RF	-54.1263076327005
25	H4	120	S3	RF	-67.4544750096861
25	H4	120	S4	RF	-16.7376985664471
25	H4	120	S1	BB	96.4788732394366
25	H4	120	S2	BB	716.197183098592
25	H4	120	S3	BB	334.154929577465
25	H4	120	S4	BB	40.1408450704225
25	H4	120	S1	FCU	225.5
25	H4	120	S2	FCU	796.5
25	H4	120	S3	FCU	599.25
25	H4	120	S4	FCU	55.25
25	H4	120	S1	ES	24.1461477362987
25	H4	120	S2	ES	8.10166799046862
25	H4	120	S3	ES	19.0627482128674
25	H4	120	S4	ES	28.9118347895155
25	H4	120	S1	MG	2.56678889470926
25	H4	120	S2	MG	25.8250392875851
25	H4	120	S3	MG	15.4007333682556
25	H4	120	S4	MG	-24.8559455212153
25	H4	120	S1	LM	75.4415475189235
25	H4	120	S2	LM	482.842724978974
25	H4	120	S3	LM	50.7989907485282
25	H4	120	S4	LM	184.104289318755
26	H1	60	S1	RF	62.5521765056649
26	H1	60	S2	RF	-60.3458556946929
26	H1	60	S3	RF	-62.6714370900417

Continued on next page

Participant_ID	Seat_Height	Seat_Height_pct_PH	Stage	Muscle	Delta_RMS_pct
26	H1	60	S4	RF	91.1150864639237
26	H1	60	S1	BB	185.222381635581
26	H1	60	S2	BB	500
26	H1	60	S3	BB	854.232424677188
26	H1	60	S4	BB	181.635581061693
26	H1	60	S1	FCU	-71.3666228646518
26	H1	60	S2	FCU	-82.7201051248357
26	H1	60	S3	FCU	-87.8318002628121
26	H1	60	S4	FCU	-69.9080157687254
26	H1	60	S1	ES	558.724428399519
26	H1	60	S2	ES	17.9302045728038
26	H1	60	S3	ES	79.3020457280385
26	H1	60	S4	ES	713.477737665463
26	H1	60	S1	MG	-34.8719034511963
26	H1	60	S2	MG	-66.6313783612111
26	H1	60	S3	MG	-86.9574423036206
26	H1	60	S4	MG	-26.8049968240525
26	H1	60	S1	LM	-68.569572078344
26	H1	60	S2	LM	-85.1016429963799
26	H1	60	S3	LM	-78.3904204956837
26	H1	60	S4	LM	-71.6699155295646
26	H2	80	S1	RF	-6.69334303383048
26	H2	80	S2	RF	-41.7606402328119
26	H2	80	S3	RF	-50.8184794470717
26	H2	80	S4	RF	9.85813022917425
26	H2	80	S1	BB	-56.7421513124035
26	H2	80	S2	BB	-15.748841996912
26	H2	80	S3	BB	-64.8481729284611
26	H2	80	S4	BB	-56.8708183221822
26	H2	80	S1	FCU	126.691729323308
26	H2	80	S2	FCU	-30.5263157894737
26	H2	80	S3	FCU	-2.10526315789475
26	H2	80	S4	FCU	125.488721804511
26	H2	80	S1	ES	41.2414689632759
26	H2	80	S2	ES	10.4972375690608
26	H2	80	S3	ES	-62.5284367890803
26	H2	80	S4	ES	79.8830029249269
26	H2	80	S1	MG	-29.7860199714693
26	H2	80	S2	MG	-62.1968616262482
26	H2	80	S3	MG	-59.6576319543509
26	H2	80	S4	MG	-4.22253922967189
26	H2	80	S1	LM	-73.1333758774729
26	H2	80	S2	LM	-82.8015315890236
26	H2	80	S3	LM	-79.0789193788556
26	H2	80	S4	LM	-66.1986811316741
26	H3	100	S1	RF	60.6786427145709
26	H3	100	S2	RF	153.792415169661
26	H3	100	S3	RF	-26.8463073852295
26	H3	100	S4	RF	160.578842315369
26	H3	100	S1	BB	70.687237026648
26	H3	100	S2	BB	1240.53295932679
26	H3	100	S3	BB	716.549789621318
26	H3	100	S4	BB	65.9186535764376
26	H3	100	S1	FCU	-66.1081492764661
26	H3	100	S2	FCU	371.210967250571
26	H3	100	S3	FCU	-21.2490479817213

Continued on next page

Participant_ID	Seat_Height	Seat_Height_pct_PH	Stage	Muscle	Delta_RMS_pct
26	H3	100	S4	FCU	-56.8418380299568
26	H3	100	S1	ES	932.047477744807
26	H3	100	S2	ES	80.1186943620178
26	H3	100	S3	ES	65.8753709198813
26	H3	100	S4	ES	1300
26	H3	100	S1	MG	-54.490861618799
26	H3	100	S2	MG	-77.5718015665796
26	H3	100	S3	MG	-90.9921671018277
26	H3	100	S4	MG	-30.5483028720627
26	H3	100	S1	LM	103.532875368008
26	H3	100	S2	LM	152.894995093229
26	H3	100	S3	LM	181.746810598626
26	H3	100	S4	LM	237.095191364082
26	H4	120	S1	RF	-31.9327731092437
26	H4	120	S2	RF	-61.8247298919568
26	H4	120	S3	RF	-52.9411764705882
26	H4	120	S4	RF	3.00120048019208
26	H4	120	S1	BB	-4.25038639876352
26	H4	120	S2	BB	538.948995363215
26	H4	120	S3	BB	89.4126738794436
26	H4	120	S4	BB	-20.8655332302937
26	H4	120	S1	FCU	-56.2297496318115
26	H4	120	S2	FCU	276.288659793814
26	H4	120	S3	FCU	-35.640648011782
26	H4	120	S4	FCU	-60.1767304860088
26	H4	120	S1	ES	433.940397350993
26	H4	120	S2	ES	38.7417218543047
26	H4	120	S3	ES	38.41059602649
26	H4	120	S4	ES	632.450331125828
26	H4	120	S1	MG	-41.029702970297
26	H4	120	S2	MG	-38.1782178217822
26	H4	120	S3	MG	-64.7920792079208
26	H4	120	S4	MG	-5.54455445544554
26	H4	120	S1	LM	83.3470733718054
26	H4	120	S2	LM	129.348722176422
26	H4	120	S3	LM	100
26	H4	120	S4	LM	142.621599340478
27	H1	60	S1	RF	17.7988242978445
27	H1	60	S2	RF	-53.8863487916394
27	H1	60	S3	RF	-53.4291312867407
27	H1	60	S4	RF	35.9568909209667
27	H1	60	S1	BB	265.492957746479
27	H1	60	S2	BB	577.934272300469
27	H1	60	S3	BB	528.169014084507
27	H1	60	S4	BB	195.539906103286
27	H1	60	S1	FCU	120.985221674877
27	H1	60	S2	FCU	221.083743842365
27	H1	60	S3	FCU	353.300492610837
27	H1	60	S4	FCU	85.7142857142857
27	H1	60	S1	ES	1081.23076923077
27	H1	60	S2	ES	417.846153846154
27	H1	60	S3	ES	34.7692307692308
27	H1	60	S4	ES	1317.53846153846
27	H1	60	S1	MG	-20.2563301031572
27	H1	60	S2	MG	52.9227883713661
27	H1	60	S3	MG	17.9118474523289

Continued on next page

Participant_ID	Seat_Height	Seat_Height_pct_PH	Stage	Muscle	Delta_RMS_pct
27	H1	60	S4	MG	6.43951234760862
27	H1	60	S1	LM	222.245108135942
27	H1	60	S2	LM	239.031925849639
27	H1	60	S3	LM	290.113285272914
27	H1	60	S4	LM	236.97219361483
27	H2	80	S1	RF	-25.2806246949731
27	H2	80	S2	RF	-76.232308443143
27	H2	80	S3	RF	5.75890678379699
27	H2	80	S4	RF	-6.14934114202049
27	H2	80	S1	BB	184.151785714286
27	H2	80	S2	BB	797.321428571428
27	H2	80	S3	BB	576.5625
27	H2	80	S4	BB	144.196428571428
27	H2	80	S1	FCU	75.6011730205279
27	H2	80	S2	FCU	96.891495601173
27	H2	80	S3	FCU	230.733137829912
27	H2	80	S4	FCU	40.8797653958944
27	H2	80	S1	ES	346.717557251908
27	H2	80	S2	ES	611.75572519084
27	H2	80	S3	ES	218.167938931298
27	H2	80	S4	ES	513.282442748092
27	H2	80	S1	MG	-56.7944977073781
27	H2	80	S2	MG	-29.5122967903293
27	H2	80	S3	MG	18.174239266361
27	H2	80	S4	MG	-32.1175489787411
27	H2	80	S1	LM	88.1399317406143
27	H2	80	S2	LM	67.8327645051195
27	H2	80	S3	LM	261.860068259386
27	H2	80	S4	LM	176.279863481229
27	H3	100	S1	RF	-25.8782936010038
27	H3	100	S2	RF	-67.4404015056462
27	H3	100	S3	RF	-64.0840652446675
27	H3	100	S4	RF	-2.54077791718946
27	H3	100	S1	BB	93.8534278959811
27	H3	100	S2	BB	427.186761229314
27	H3	100	S3	BB	214.184397163121
27	H3	100	S4	BB	93.144208037825
27	H3	100	S1	FCU	-41.7642140468227
27	H3	100	S2	FCU	8.57023411371236
27	H3	100	S3	FCU	291.26254180602
27	H3	100	S4	FCU	-50.2090301003345
27	H3	100	S1	ES	986.09022556391
27	H3	100	S2	ES	446.240601503759
27	H3	100	S3	ES	254.887218045113
27	H3	100	S4	ES	1228.57142857143
27	H3	100	S1	MG	19.1287878787879
27	H3	100	S2	MG	-17.3611111111111
27	H3	100	S3	MG	136.805555555555
27	H3	100	S4	MG	64.6464646464646
27	H3	100	S1	LM	102.283105022831
27	H3	100	S2	LM	138.812785388128
27	H3	100	S3	LM	277.625570776256
27	H3	100	S4	LM	247.762557077626
27	H4	120	S1	RF	32.874139010645
27	H4	120	S2	RF	68.6912961803381
27	H4	120	S3	RF	533.124608641202

Continued on next page

Participant_ID	Seat_Height	Seat_Height_pct_PH	Stage	Muscle	Delta_RMS_pct
27	H4	120	S4	RF	96.7438948027552
27	H4	120	S1	BB	-46.8783638320775
27	H4	120	S2	BB	129.224973089343
27	H4	120	S3	BB	-26.210979547901
27	H4	120	S4	BB	-61.0871905274489
27	H4	120	S1	FCU	-21.1390456644433
27	H4	120	S2	FCU	106.105695228322
27	H4	120	S3	FCU	121.908671113392
27	H4	120	S4	FCU	-43.5608004104669
27	H4	120	S1	ES	212.328767123288
27	H4	120	S2	ES	-40.3486924034869
27	H4	120	S3	ES	22.9140722291407
27	H4	120	S4	ES	292.029887920299
27	H4	120	S1	MG	-30.1515151515151
27	H4	120	S2	MG	34.6212121212121
27	H4	120	S3	MG	71.4015151515152
27	H4	120	S4	MG	-4.16666666666666
27	H4	120	S1	LM	25.1599147121535
27	H4	120	S2	LM	145.486851457001
27	H4	120	S3	LM	235.89196872779
27	H4	120	S4	LM	84.363894811656
28	H1	60	S1	RF	325.876288659794
28	H1	60	S2	RF	-22.8865979381443
28	H1	60	S3	RF	304.123711340206
28	H1	60	S4	RF	327.422680412371
28	H1	60	S1	BB	158.354114713217
28	H1	60	S2	BB	1178.927680798
28	H1	60	S3	BB	273.067331670823
28	H1	60	S4	BB	132.294264339152
28	H1	60	S1	FCU	872.727272727273
28	H1	60	S2	FCU	2674.70355731225
28	H1	60	S3	FCU	5504.74308300395
28	H1	60	S4	FCU	745.454545454545
28	H1	60	S1	ES	1020.30769230769
28	H1	60	S2	ES	417.846153846154
28	H1	60	S3	ES	34.7692307692308
28	H1	60	S4	ES	1460.30769230769
28	H1	60	S1	MG	116.654107008289
28	H1	60	S2	MG	222.908816880181
28	H1	60	S3	MG	46.4958553127355
28	H1	60	S4	MG	159.382064807837
28	H1	60	S1	LM	389.387755102041
28	H1	60	S2	LM	8.77551020408163
28	H1	60	S3	LM	158.571428571429
28	H1	60	S4	LM	561.224489795918
28	H2	80	S1	RF	4.48204214900565
28	H2	80	S2	RF	-40.3680617393885
28	H2	80	S3	RF	-12.5853368952211
28	H2	80	S4	RF	25.7346393588602
28	H2	80	S1	BB	14.0863787375415
28	H2	80	S2	BB	217.940199335548
28	H2	80	S3	BB	127.641196013289
28	H2	80	S4	BB	-3.38870431893689
28	H2	80	S1	FCU	679.945799457995
28	H2	80	S2	FCU	3130.89430894309
28	H2	80	S3	FCU	2327.91327913279

Continued on next page

Participant_ID	Seat_Height	Seat_Height_pct_PH	Stage	Muscle	Delta_RMS_pct
28	H2	80	S4	FCU	875.067750677507
28	H2	80	S1	ES	412.519083969466
28	H2	80	S2	ES	611.75572519084
28	H2	80	S3	ES	218.167938931298
28	H2	80	S4	ES	506.564885496183
28	H2	80	S1	MG	3.61744301288405
28	H2	80	S2	MG	177.502477700694
28	H2	80	S3	MG	60.3072348860258
28	H2	80	S4	MG	50.9910802775025
28	H2	80	S1	LM	272.859450726979
28	H2	80	S2	LM	154.765751211632
28	H2	80	S3	LM	218.255250403877
28	H2	80	S4	LM	406.946688206785
28	H3	100	S1	RF	28.2212086659065
28	H3	100	S2	RF	139.794754846066
28	H3	100	S3	RF	-32.3831242873432
28	H3	100	S4	RF	69.2702394526796
28	H3	100	S1	BB	144.331983805668
28	H3	100	S2	BB	294.534412955465
28	H3	100	S3	BB	305.87044534413
28	H3	100	S4	BB	130.364372469636
28	H3	100	S1	FCU	308.864265927978
28	H3	100	S2	FCU	430.470914127424
28	H3	100	S3	FCU	87.2576177285319
28	H3	100	S4	FCU	346.537396121884
28	H3	100	S1	ES	1199.24812030075
28	H3	100	S2	ES	446.240601503759
28	H3	100	S3	ES	254.887218045113
28	H3	100	S4	ES	1367.29323308271
28	H3	100	S1	MG	34.8705501618123
28	H3	100	S2	MG	97.0064724919094
28	H3	100	S3	MG	177.265372168285
28	H3	100	S4	MG	95.631067961165
28	H3	100	S1	LM	219.102990033223
28	H3	100	S2	LM	173.089700996678
28	H3	100	S3	LM	364.617940199336
28	H3	100	S4	LM	345.847176079734
28	H4	120	S1	RF	113.789778206365
28	H4	120	S2	RF	380.23143683703
28	H4	120	S3	RF	125.940212150434
28	H4	120	S4	RF	171.263259402121
28	H4	120	S1	BB	315.849056603774
28	H4	120	S2	BB	1118.11320754717
28	H4	120	S3	BB	668.679245283019
28	H4	120	S4	BB	204.905660377358
28	H4	120	S1	FCU	414.715719063545
28	H4	120	S2	FCU	5876.25418060201
28	H4	120	S3	FCU	334.448160535117
28	H4	120	S4	FCU	223.076923076923
28	H4	120	S1	ES	215.317559153176
28	H4	120	S2	ES	-40.3486924034869
28	H4	120	S3	ES	22.9140722291407
28	H4	120	S4	ES	300.498132004981
28	H4	120	S1	MG	108.375634517766
28	H4	120	S2	MG	638.071065989848
28	H4	120	S3	MG	503.426395939086

Continued on next page

Participant_ID	Seat_Height	Seat_Height_pct_PH	Stage	Muscle	Delta_RMS_pct
28	H4	120	S4	MG	198.477157360406
28	H4	120	S1	LM	202.423263327948
28	H4	120	S2	LM	27.9483037156704
28	H4	120	S3	LM	241.518578352181
28	H4	120	S4	LM	306.946688206785
29	H1	60	S1	RF	240.832595217006
29	H1	60	S2	RF	-63.4189548272808
29	H1	60	S3	RF	-24.800708591674
29	H1	60	S4	RF	264.127546501329
29	H1	60	S1	BB	88.0580357142857
29	H1	60	S2	BB	473.660714285714
29	H1	60	S3	BB	515.848214285714
29	H1	60	S4	BB	64.9553571428571
29	H1	60	S1	FCU	117.192600652884
29	H1	60	S2	FCU	72.1436343852013
29	H1	60	S3	FCU	101.414581066376
29	H1	60	S4	FCU	105.44069640914
29	H1	60	S1	ES	117.41935483871
29	H1	60	S2	ES	441.129032258065
29	H1	60	S3	ES	375
29	H1	60	S4	ES	228.225806451613
29	H1	60	S1	MG	-61.8103910016069
29	H1	60	S2	MG	-33.4895554365292
29	H1	60	S3	MG	-31.2265666845206
29	H1	60	S4	MG	-52.9994643813605
29	H1	60	S1	LM	243.023255813954
29	H1	60	S2	LM	26.046511627907
29	H1	60	S3	LM	128.372093023256
29	H1	60	S4	LM	312.441860465116
29	H2	80	S1	RF	158.333333333333
29	H2	80	S2	RF	-51.3754045307443
29	H2	80	S3	RF	47.0873786407767
29	H2	80	S4	RF	212.216828478964
29	H2	80	S1	BB	107.072368421053
29	H2	80	S2	BB	178.453947368421
29	H2	80	S3	BB	650
29	H2	80	S4	BB	87.0065789473684
29	H2	80	S1	FCU	98.2142857142857
29	H2	80	S2	FCU	61.7857142857143
29	H2	80	S3	FCU	140.267857142857
29	H2	80	S4	FCU	96.6964285714286
29	H2	80	S1	ES	124.978317432784
29	H2	80	S2	ES	499.306157849089
29	H2	80	S3	ES	407.025151777971
29	H2	80	S4	ES	156.201214223764
29	H2	80	S1	MG	-58.5344827586207
29	H2	80	S2	MG	-11.4310344827586
29	H2	80	S3	MG	-21.3275862068966
29	H2	80	S4	MG	-41.3448275862069
29	H2	80	S1	LM	164.070351758794
29	H2	80	S2	LM	29.6482412060302
29	H2	80	S3	LM	119.974874371859
29	H2	80	S4	LM	287.311557788945
29	H3	100	S1	RF	236.734693877551
29	H3	100	S2	RF	97.2789115646259
29	H3	100	S3	RF	527.210884353742

Continued on next page

Participant_ID	Seat_Height	Seat_Height_pct_PH	Stage	Muscle	Delta_RMS_pct
29	H3	100	S4	RF	333.061224489796
29	H3	100	S1	BB	211.799410029498
29	H3	100	S2	BB	584.070796460177
29	H3	100	S3	BB	263.716814159292
29	H3	100	S4	BB	201.769911504425
29	H3	100	S1	FCU	31.0909090909091
29	H3	100	S2	FCU	108.818181818182
29	H3	100	S3	FCU	132.363636363636
29	H3	100	S4	FCU	27.3636363636364
29	H3	100	S1	ES	369.425675675676
29	H3	100	S2	ES	575.844594594595
29	H3	100	S3	ES	358.952702702703
29	H3	100	S4	ES	445.101351351351
29	H3	100	S1	MG	-65.4517374517374
29	H3	100	S2	MG	-55.4285714285714
29	H3	100	S3	MG	-50.7799227799228
29	H3	100	S4	MG	-59.1042471042471
29	H3	100	S1	LM	161.684782608696
29	H3	100	S2	LM	125.407608695652
29	H3	100	S3	LM	67.6630434782609
29	H3	100	S4	LM	283.152173913043
29	H4	120	S1	RF	111.814744801512
29	H4	120	S2	RF	210.302457466919
29	H4	120	S3	RF	251.512287334594
29	H4	120	S4	RF	215.595463137996
29	H4	120	S1	BB	131.094049904031
29	H4	120	S2	BB	1087.71593090211
29	H4	120	S3	BB	594.433781190019
29	H4	120	S4	BB	51.2476007677543
29	H4	120	S1	FCU	50.1776198934281
29	H4	120	S2	FCU	155.595026642984
29	H4	120	S3	FCU	61.7229129662522
29	H4	120	S4	FCU	-9.05861456483126
29	H4	120	S1	ES	382.402707275804
29	H4	120	S2	ES	890.524534686971
29	H4	120	S3	ES	800.846023688663
29	H4	120	S4	ES	513.705583756345
29	H4	120	S1	MG	-13.0169172932331
29	H4	120	S2	MG	266.259398496241
29	H4	120	S3	MG	301.644736842105
29	H4	120	S4	MG	21.7105263157895
29	H4	120	S1	LM	96.8511450381679
29	H4	120	S2	LM	97.0419847328244
29	H4	120	S3	LM	98.1870229007633
29	H4	120	S4	LM	173.187022900763
30	H1	60	S1	RF	84.3550760176557
30	H1	60	S2	RF	33.1535066208926
30	H1	60	S3	RF	170.328592447278
30	H1	60	S4	RF	75.8705247670427
30	H1	60	S1	BB	152.352231604343
30	H1	60	S2	BB	805.669481302774
30	H1	60	S3	BB	780.579010856454
30	H1	60	S4	BB	146.803377563329
30	H1	60	S1	FCU	-49.4752006585717
30	H1	60	S2	FCU	14.8590244906359
30	H1	60	S3	FCU	27.5776908828977

Continued on next page

Participant_ID	Seat_Height	Seat_Height_pct_PH	Stage	Muscle	Delta_RMS_pct
30	H1	60	S4	FCU	-37.3945256225561
30	H1	60	S1	ES	366.699314397649
30	H1	60	S2	ES	851.518119490695
30	H1	60	S3	ES	178.45249755142
30	H1	60	S4	ES	411.851126346719
30	H1	60	S1	MG	-58.4124665205746
30	H1	60	S2	MG	-42.5249573898223
30	H1	60	S3	MG	-34.4411979547115
30	H1	60	S4	MG	-57.4506939371804
30	H1	60	S1	LM	-35.036496350365
30	H1	60	S2	LM	16.0414191139026
30	H1	60	S3	LM	100.746902053981
30	H1	60	S4	LM	-37.8034289594296
30	H2	80	S1	RF	26.5776140027637
30	H2	80	S2	RF	-42.7452786734224
30	H2	80	S3	RF	39.1985260248733
30	H2	80	S4	RF	69.0465223399355
30	H2	80	S1	BB	158.857142857143
30	H2	80	S2	BB	2055.80952380952
30	H2	80	S3	BB	706.285714285714
30	H2	80	S4	BB	219.619047619048
30	H2	80	S1	FCU	-13.3482000637146
30	H2	80	S2	FCU	359.541255176808
30	H2	80	S3	FCU	107.582032494425
30	H2	80	S4	FCU	7.13603058298822
30	H2	80	S1	ES	1101.89274447949
30	H2	80	S2	ES	2508.20189274448
30	H2	80	S3	ES	4692.74447949527
30	H2	80	S4	ES	1488.64353312303
30	H2	80	S1	MG	-76.9600905745825
30	H2	80	S2	MG	-45.9477309180111
30	H2	80	S3	MG	-40.1452967261062
30	H2	80	S4	MG	-64.704217378998
30	H2	80	S1	LM	-41.189725101397
30	H2	80	S2	LM	106.782334384858
30	H2	80	S3	LM	-4.6867958539883
30	H2	80	S4	LM	-21.5637674628211
30	H3	100	S1	RF	-23.2537042636831
30	H3	100	S2	RF	4.23344420925309
30	H3	100	S3	RF	178.016328999093
30	H3	100	S4	RF	2.78197762322347
30	H3	100	S1	BB	-49.8724024790375
30	H3	100	S2	BB	145.679912504557
30	H3	100	S3	BB	126.57674079475
30	H3	100	S4	BB	-39.9197958439665
30	H3	100	S1	FCU	-41.2571046472752
30	H3	100	S2	FCU	115.513206285523
30	H3	100	S3	FCU	237.445670344366
30	H3	100	S4	FCU	-25.810765630224
30	H3	100	S1	ES	44.7635135135135
30	H3	100	S2	ES	224.619932432432
30	H3	100	S3	ES	127.660472972973
30	H3	100	S4	ES	86.6976351351351
30	H3	100	S1	MG	-63.4199527530438
30	H3	100	S2	MG	-34.5084499363983
30	H3	100	S3	MG	4.45211702707613

Continued on next page

Participant_ID	Seat_Height	Seat_Height_pct_PH	Stage	Muscle	Delta_RMS_pct
30	H3	100	S4	MG	-49.8091949845539
30	H3	100	S1	LM	1.90303579519709
30	H3	100	S2	LM	145.44630720435
30	H3	100	S3	LM	239.737199818759
30	H3	100	S4	LM	67.8296329859538
30	H4	120	S1	RF	42.9692609548725
30	H4	120	S2	RF	230.215827338129
30	H4	120	S3	RF	477.697841726619
30	H4	120	S4	RF	109.614126880314
30	H4	120	S1	BB	45.1472191930207
30	H4	120	S2	BB	587.568157033806
30	H4	120	S3	BB	730.316248636859
30	H4	120	S4	BB	-0.218102508178839
30	H4	120	S1	FCU	-59.5510376958916
30	H4	120	S2	FCU	108.238034731046
30	H4	120	S3	FCU	166.221939855993
30	H4	120	S4	FCU	-73.2316814908937
30	H4	120	S1	ES	506.923076923077
30	H4	120	S2	ES	2141.73076923077
30	H4	120	S3	ES	1584.23076923077
30	H4	120	S4	ES	693.846153846154
30	H4	120	S1	MG	-58.8616071428571
30	H4	120	S2	MG	1.96428571428572
30	H4	120	S3	MG	10.4017857142857
30	H4	120	S4	MG	-41.3392857142857
30	H4	120	S1	LM	-13.7805684071381
30	H4	120	S2	LM	79.5109054857898
30	H4	120	S3	LM	28.0568407138136
30	H4	120	S4	LM	9.51751487111698
31	H1	60	S1	RF	50.9649468294604
31	H1	60	S2	RF	-11.7762898779047
31	H1	60	S3	RF	11.0279637652619
31	H1	60	S4	RF	60.4568727845609
31	H1	60	S1	BB	156.025039123631
31	H1	60	S2	BB	454.14710485133
31	H1	60	S3	BB	321.596244131455
31	H1	60	S4	BB	124.100156494523
31	H1	60	S1	FCU	35.5017301038062
31	H1	60	S2	FCU	-32.3875432525952
31	H1	60	S3	FCU	18.6851211072664
31	H1	60	S4	FCU	30.6574394463668
31	H1	60	S1	ES	68.7875150060024
31	H1	60	S2	ES	250
31	H1	60	S3	ES	154.081632653061
31	H1	60	S4	ES	148.019207683073
31	H1	60	S1	MG	-44.3080989239192
31	H1	60	S2	MG	6.75854257126675
31	H1	60	S3	MG	-33.452897866717
31	H1	60	S4	MG	-31.5650368132905
31	H1	60	S1	LM	145.091514143095
31	H1	60	S2	LM	-17.9700499168053
31	H1	60	S3	LM	56.405990016639
31	H1	60	S4	LM	193.760399334443
31	H2	80	S1	RF	26.2825572217837
31	H2	80	S2	RF	-9.94475138121547
31	H2	80	S3	RF	151.381215469613

Continued on next page

Participant_ID	Seat_Height	Seat_Height_pct_PH	Stage	Muscle	Delta_RMS_pct
31	H2	80	S4	RF	52.1704814522494
31	H2	80	S1	BB	282.674772036474
31	H2	80	S2	BB	1277.50759878419
31	H2	80	S3	BB	849.240121580547
31	H2	80	S4	BB	238.905775075988
31	H2	80	S1	FCU	49.1310160427807
31	H2	80	S2	FCU	-5.34759358288771
31	H2	80	S3	FCU	43.5828877005348
31	H2	80	S4	FCU	45.3208556149732
31	H2	80	S1	ES	112.359550561798
31	H2	80	S2	ES	669.341894060995
31	H2	80	S3	ES	181.540930979133
31	H2	80	S4	ES	147.913322632424
31	H2	80	S1	MG	-25.5043661547727
31	H2	80	S2	MG	461.096055404998
31	H2	80	S3	MG	218.277627220717
31	H2	80	S4	MG	4.21559771153267
31	H2	80	S1	LM	156.445556946183
31	H2	80	S2	LM	149.561952440551
31	H2	80	S3	LM	129.286608260325
31	H2	80	S4	LM	279.849812265332
31	H3	100	S1	RF	-12.090236164963
31	H3	100	S2	RF	-24.9911878745153
31	H3	100	S3	RF	4.33556573845612
31	H3	100	S4	RF	12.0197391610856
31	H3	100	S1	BB	186.274509803922
31	H3	100	S2	BB	939.21568627451
31	H3	100	S3	BB	489.075630252101
31	H3	100	S4	BB	180.952380952381
31	H3	100	S1	FCU	1.79856115107914
31	H3	100	S2	FCU	-8.70503597122303
31	H3	100	S3	FCU	143.597122302158
31	H3	100	S4	FCU	2.3021582733813
31	H3	100	S1	ES	19.814502529511
31	H3	100	S2	ES	161.677908937605
31	H3	100	S3	ES	-32.3777403035413
31	H3	100	S4	ES	40.2613827993255
31	H3	100	S1	MG	-54.2845755280988
31	H3	100	S2	MG	-7.65245117576723
31	H3	100	S3	MG	-55.1215623754484
31	H3	100	S4	MG	-46.2534874451973
31	H3	100	S1	LM	43.6704119850187
31	H3	100	S2	LM	-19.0262172284644
31	H3	100	S3	LM	63.2209737827715
31	H3	100	S4	LM	111.161048689139
31	H4	120	S1	RF	-49.5287253141831
31	H4	120	S2	RF	-56.665170556553
31	H4	120	S3	RF	-63.8689407540395
31	H4	120	S4	RF	-25.0448833034111
31	H4	120	S1	BB	233.625730994152
31	H4	120	S2	BB	1949.12280701754
31	H4	120	S3	BB	148.538011695906
31	H4	120	S4	BB	124.853801169591
31	H4	120	S1	FCU	26.8689859363434
31	H4	120	S2	FCU	113.027387120651
31	H4	120	S3	FCU	58.7712805329386

Continued on next page

Participant_ID	Seat_Height	Seat_Height_pct_PH	Stage	Muscle	Delta_RMS_pct
31	H4	120	S4	FCU	-24.500370096225
31	H4	120	S1	ES	639.285714285714
31	H4	120	S2	ES	2679.84693877551
31	H4	120	S3	ES	157.397959183673
31	H4	120	S4	ES	836.224489795918
31	H4	120	S1	MG	-69.8668754894284
31	H4	120	S2	MG	21.7384494909945
31	H4	120	S3	MG	-52.3414252153485
31	H4	120	S4	MG	-58.8097102584182
31	H4	120	S1	LM	107.238883143743
31	H4	120	S2	LM	36.2978283350569
31	H4	120	S3	LM	199.172699069286
31	H4	120	S4	LM	191.726990692864
32	H1	60	S1	RF	-44
32	H1	60	S2	RF	-73.3333333333333
32	H1	60	S3	RF	-74.3366336633663
32	H1	60	S4	RF	-40.1320132013201
32	H1	60	S1	BB	388.54415274463
32	H1	60	S2	BB	1052.50596658711
32	H1	60	S3	BB	2551.31264916468
32	H1	60	S4	BB	315.513126491647
32	H1	60	S1	FCU	52.3370233702337
32	H1	60	S2	FCU	11.7466174661747
32	H1	60	S3	FCU	19.249692496925
32	H1	60	S4	FCU	28.290282902829
32	H1	60	S1	ES	845.189504373178
32	H1	60	S2	ES	1423.90670553936
32	H1	60	S3	ES	817.492711370262
32	H1	60	S4	ES	1180.75801749271
32	H1	60	S1	MG	-87.3602497574147
32	H1	60	S2	MG	-67.8690461123065
32	H1	60	S3	MG	-77.2644812892883
32	H1	60	S4	MG	-85.4406615196389
32	H1	60	S1	LM	238.110072689512
32	H1	60	S2	LM	70.9241952232606
32	H1	60	S3	LM	52.02492211838
32	H1	60	S4	LM	289.927310488058
32	H2	80	S1	RF	-34.9313780785862
32	H2	80	S2	RF	-30.2312464749013
32	H2	80	S3	RF	-29.8552359466065
32	H2	80	S4	RF	-21.3197969543147
32	H2	80	S1	BB	116.983894582723
32	H2	80	S2	BB	603.367496339678
32	H2	80	S3	BB	656.954612005857
32	H2	80	S4	BB	99.8535871156662
32	H2	80	S1	FCU	7.46887966804978
32	H2	80	S2	FCU	3.27339787920699
32	H2	80	S3	FCU	-4.14937759336101
32	H2	80	S4	FCU	9.0825265099124
32	H2	80	S1	ES	708.923884514436
32	H2	80	S2	ES	1472.17847769029
32	H2	80	S3	ES	1408.13648293963
32	H2	80	S4	ES	845.14435695538
32	H2	80	S1	MG	-63.5262661214218
32	H2	80	S2	MG	77.9647687952186
32	H2	80	S3	MG	-34.6178043409877

Continued on next page

Participant_ID	Seat_Height	Seat_Height_pct_PH	Stage	Muscle	Delta_RMS_pct
32	H2	80	S4	MG	-42.9223026108839
32	H2	80	S1	LM	157.662624035281
32	H2	80	S2	LM	17.3098125689085
32	H2	80	S3	LM	24.696802646086
32	H2	80	S4	LM	265.270121278942
32	H3	100	S1	RF	-53.6274208422994
32	H3	100	S2	RF	-79.4804795573317
32	H3	100	S3	RF	-13.449123885644
32	H3	100	S4	RF	-45.4964648017215
32	H3	100	S1	BB	206.534090909091
32	H3	100	S2	BB	916.477272727273
32	H3	100	S3	BB	751.988636363636
32	H3	100	S4	BB	224.147727272727
32	H3	100	S1	FCU	20
32	H3	100	S2	FCU	69.0909090909091
32	H3	100	S3	FCU	-34.7636363636364
32	H3	100	S4	FCU	12.3636363636364
32	H3	100	S1	ES	1163.24110671937
32	H3	100	S2	ES	1539.5256916996
32	H3	100	S3	ES	2440.71146245059
32	H3	100	S4	ES	1370.3557312253
32	H3	100	S1	MG	-78.0331924543862
32	H3	100	S2	MG	-67.1580249458819
32	H3	100	S3	MG	-14.8541387485826
32	H3	100	S4	MG	-71.6730233996495
32	H3	100	S1	LM	277.313974591652
32	H3	100	S2	LM	113.248638838476
32	H3	100	S3	LM	87.8402903811252
32	H3	100	S4	LM	466.606170598911
32	H4	120	S1	RF	-64.9825558857734
32	H4	120	S2	RF	-80.1783176120946
32	H4	120	S3	RF	-57.0874790024551
32	H4	120	S4	RF	-54.6194598785373
32	H4	120	S1	BB	60.1977750309024
32	H4	120	S2	BB	357.478368355995
32	H4	120	S3	BB	282.941903584672
32	H4	120	S4	BB	0.988875154511744
32	H4	120	S1	FCU	6.61103979460847
32	H4	120	S2	FCU	40.5648267008986
32	H4	120	S3	FCU	162.901155327343
32	H4	120	S4	FCU	-36.9062901155327
32	H4	120	S1	ES	698.108108108108
32	H4	120	S2	ES	855.405405405405
32	H4	120	S3	ES	1374.05405405405
32	H4	120	S4	ES	803.243243243243
32	H4	120	S1	MG	-83.1989359048965
32	H4	120	S2	MG	-73.0567794496633
32	H4	120	S3	MG	-23.0775625571535
32	H4	120	S4	MG	-78.0114722753346
32	H4	120	S1	LM	118.206039076377
32	H4	120	S2	LM	32.2380106571936
32	H4	120	S3	LM	34.0142095914742
32	H4	120	S4	LM	180.905861456483
33	H1	60	S1	RF	464.896755162242
33	H1	60	S2	RF	26.401179941003
33	H1	60	S3	RF	399.262536873156

Continued on next page

Participant_ID	Seat_Height	Seat_Height_pct_PH	Stage	Muscle	Delta_RMS_pct
33	H1	60	S4	RF	499.557522123894
33	H1	60	S1	BB	307.130124777184
33	H1	60	S2	BB	354.545454545455
33	H1	60	S3	BB	412.121212121212
33	H1	60	S4	BB	248.484848484848
33	H1	60	S1	FCU	58.1879194630872
33	H1	60	S2	FCU	18.1879194630872
33	H1	60	S3	FCU	48.3892617449664
33	H1	60	S4	FCU	36.1073825503356
33	H1	60	S1	ES	895.013850415513
33	H1	60	S2	ES	104.15512465374
33	H1	60	S3	ES	1583.65650969529
33	H1	60	S4	ES	1352.90858725762
33	H1	60	S1	MG	22.0957613814757
33	H1	60	S2	MG	58.3987441130298
33	H1	60	S3	MG	176.609105180534
33	H1	60	S4	MG	37.0879120879121
33	H1	60	S1	LM	61.2281757977122
33	H1	60	S2	LM	230.885009030704
33	H1	60	S3	LM	60.2046959662854
33	H1	60	S4	LM	94.4611679711017
33	H2	80	S1	RF	193.103448275862
33	H2	80	S2	RF	-48.9463601532567
33	H2	80	S3	RF	-37.6436781609195
33	H2	80	S4	RF	275.191570881226
33	H2	80	S1	BB	183.156028368794
33	H2	80	S2	BB	653.723404255319
33	H2	80	S3	BB	540.780141843972
33	H2	80	S4	BB	215.248226950355
33	H2	80	S1	FCU	77.8705636743215
33	H2	80	S2	FCU	57.6896311760612
33	H2	80	S3	FCU	52.2616562282533
33	H2	80	S4	FCU	114.19624217119
33	H2	80	S1	ES	459.363957597173
33	H2	80	S2	ES	1560.60070671378
33	H2	80	S3	ES	1312.72084805654
33	H2	80	S4	ES	566.077738515901
33	H2	80	S1	MG	-9.17721518987342
33	H2	80	S2	MG	52.2151898734177
33	H2	80	S3	MG	91.4952531645569
33	H2	80	S4	MG	35.2848101265823
33	H2	80	S1	LM	-45.1800232288037
33	H2	80	S2	LM	-10.4761904761905
33	H2	80	S3	LM	-38.397212543554
33	H2	80	S4	LM	-25.9465737514518
33	H3	100	S1	RF	48.3311938382542
33	H3	100	S2	RF	6.93196405648267
33	H3	100	S3	RF	83.3761232349166
33	H3	100	S4	RF	101.797175866495
33	H3	100	S1	BB	132.58785942492
33	H3	100	S2	BB	398.562300319489
33	H3	100	S3	BB	206.869009584665
33	H3	100	S4	BB	99.0415335463259
33	H3	100	S1	FCU	12.387676508344
33	H3	100	S2	FCU	6.93196405648267
33	H3	100	S3	FCU	83.3761232349166

Continued on next page

Participant_ID	Seat_Height	Seat_Height_pct_PH	Stage	Muscle	Delta_RMS_pct
33	H3	100	S4	FCU	1.02695763799743
33	H3	100	S1	ES	591.533180778032
33	H3	100	S2	ES	1741.18993135011
33	H3	100	S3	ES	882.837528604119
33	H3	100	S4	ES	759.954233409611
33	H3	100	S1	MG	-26.228323699422
33	H3	100	S2	MG	-20.2312138728324
33	H3	100	S3	MG	-18.0635838150289
33	H3	100	S4	MG	-0.975433526011559
33	H3	100	S1	LM	1.10192837465566
33	H3	100	S2	LM	56.0330578512397
33	H3	100	S3	LM	91.3498622589532
33	H3	100	S4	LM	51.8457300275482
33	H4	120	S1	RF	188.985507246377
33	H4	120	S2	RF	133.478260869565
33	H4	120	S3	RF	107.971014492754
33	H4	120	S4	RF	305.942028985507
33	H4	120	S1	BB	108.562197092084
33	H4	120	S2	BB	257.18901453958
33	H4	120	S3	BB	183.683360258481
33	H4	120	S4	BB	38.610662358643
33	H4	120	S1	FCU	-6.59988214496169
33	H4	120	S2	FCU	-5.0088391278727
33	H4	120	S3	FCU	56.9829110194461
33	H4	120	S4	FCU	-36.8886269888038
33	H4	120	S1	ES	372.307692307692
33	H4	120	S2	ES	1238.07692307692
33	H4	120	S3	ES	625.576923076923
33	H4	120	S4	ES	582.692307692308
33	H4	120	S1	MG	25.1228070175438
33	H4	120	S2	MG	754.59649122807
33	H4	120	S3	MG	130.526315789474
33	H4	120	S4	MG	82.9473684210526
33	H4	120	S1	LM	1.87845303867403
33	H4	120	S2	LM	-4.75138121546963
33	H4	120	S3	LM	48.3425414364641
33	H4	120	S4	LM	59.2817679558011
34	H1	60	S1	RF	56.6037735849057
34	H1	60	S2	RF	-10.6469002695418
34	H1	60	S3	RF	58.1311769991015
34	H1	60	S4	RF	81.266846361186
34	H1	60	S1	BB	-56.8132086731649
34	H1	60	S2	BB	119.992042967973
34	H1	60	S3	BB	672.32942112592
34	H1	60	S4	BB	-60.9309727471653
34	H1	60	S1	FCU	78.6367414796342
34	H1	60	S2	FCU	9.47630922693267
34	H1	60	S3	FCU	14.04821280133
34	H1	60	S4	FCU	94.0980881130507
34	H1	60	S1	ES	89.7924608216857
34	H1	60	S2	ES	894.917407878018
34	H1	60	S3	ES	2710.88521812791
34	H1	60	S4	ES	114.781872088098
34	H1	60	S1	MG	-92.3628567203904
34	H1	60	S2	MG	-81.5515796737148
34	H1	60	S3	MG	-84.4817388732811

Continued on next page

Participant_ID	Seat_Height	Seat_Height_pct_PH	Stage	Muscle	Delta_RMS_pct
34	H1	60	S4	MG	-91.4608901375129
34	H1	60	S1	LM	359.581497797357
34	H1	60	S2	LM	34.9118942731277
34	H1	60	S3	LM	46.1453744493392
34	H1	60	S4	LM	303.303964757709
34	H2	80	S1	RF	20.1649484536083
34	H2	80	S2	RF	-11.8350515463918
34	H2	80	S3	RF	0.12371134020619
34	H2	80	S4	RF	54.5979381443299
34	H2	80	S1	BB	-70.402838655813
34	H2	80	S2	BB	181.79920684617
34	H2	80	S3	BB	507.284491755375
34	H2	80	S4	BB	-64.3706950532248
34	H2	80	S1	FCU	109.200743494424
34	H2	80	S2	FCU	52.6951672862454
34	H2	80	S3	FCU	20.910780669145
34	H2	80	S4	FCU	114.219330855019
34	H2	80	S1	ES	292.75053304904
34	H2	80	S2	ES	4192.96375266525
34	H2	80	S3	ES	2930.70362473347
34	H2	80	S4	ES	394.136460554371
34	H2	80	S1	MG	-80.5215910849714
34	H2	80	S2	MG	-51.81860393128
34	H2	80	S3	MG	-56.1445596656864
34	H2	80	S4	MG	-69.7183098591549
34	H2	80	S1	LM	191.125541125541
34	H2	80	S2	LM	39.7186147186147
34	H2	80	S3	LM	23.7012987012987
34	H2	80	S4	LM	277.922077922078
34	H3	100	S1	RF	28.8639687957094
34	H3	100	S2	RF	13.5543637250122
34	H3	100	S3	RF	193.905411994149
34	H3	100	S4	RF	64.4076060458313
34	H3	100	S1	BB	-71.97265625
34	H3	100	S2	BB	190.72265625
34	H3	100	S3	BB	603.076171875
34	H3	100	S4	BB	-67.333984375
34	H3	100	S1	FCU	27.4611398963731
34	H3	100	S2	FCU	24.006908462867
34	H3	100	S3	FCU	-48.5319516407599
34	H3	100	S4	FCU	46.3730569948186
34	H3	100	S1	ES	345.430463576159
34	H3	100	S2	ES	4188.74172185431
34	H3	100	S3	ES	6044.6357615894
34	H3	100	S4	ES	433.112582781457
34	H3	100	S1	MG	-69.4948578342408
34	H3	100	S2	MG	2.41984271022383
34	H3	100	S3	MG	102.646702964307
34	H3	100	S4	MG	-56.3369630973987
34	H3	100	S1	LM	107.410881801126
34	H3	100	S2	LM	18.0112570356473
34	H3	100	S3	LM	-5.81613508442778
34	H3	100	S4	LM	255.253283302064
34	H4	120	S1	RF	11.5768463073852
34	H4	120	S2	RF	230.289421157685
34	H4	120	S3	RF	-15.3193612774451

Continued on next page

Participant_ID	Seat_Height	Seat_Height_pct_PH	Stage	Muscle	Delta_RMS_pct
34	H4	120	S4	RF	58.9820359281437
34	H4	120	S1	BB	-18.7537447573397
34	H4	120	S2	BB	1126.12342720192
34	H4	120	S3	BB	2597.24385859796
34	H4	120	S4	BB	-46.3750748951468
34	H4	120	S1	FCU	34.3462246777164
34	H4	120	S2	FCU	60.4972375690608
34	H4	120	S3	FCU	104.235727440147
34	H4	120	S4	FCU	13.0755064456722
34	H4	120	S1	ES	13.5427952329361
34	H4	120	S2	ES	957.529794149512
34	H4	120	S3	ES	966.413867822319
34	H4	120	S4	ES	49.440231130372
34	H4	120	S1	MG	-60.9087102177554
34	H4	120	S2	MG	200.774706867672
34	H4	120	S3	MG	121.461474036851
34	H4	120	S4	MG	-44.3676716917923
34	H4	120	S1	LM	145.719844357977
34	H4	120	S2	LM	-5.54474708171205
34	H4	120	S3	LM	85.5058365758755
34	H4	120	S4	LM	215.56420233463
35	H1	60	S1	RF	347.963800904977
35	H1	60	S2	RF	-66.2141779788839
35	H1	60	S3	RF	50.2262443438914
35	H1	60	S4	RF	293.363499245852
35	H1	60	S1	BB	339.733333333333
35	H1	60	S2	BB	302.666666666667
35	H1	60	S3	BB	1112.8
35	H1	60	S4	BB	339.733333333333
35	H1	60	S1	FCU	-76.2394120577977
35	H1	60	S2	FCU	-12.9857997010463
35	H1	60	S3	FCU	5.92301943198804
35	H1	60	S4	FCU	-79.1604384653712
35	H1	60	S1	ES	1320.31558185404
35	H1	60	S2	ES	901.577909270217
35	H1	60	S3	ES	33.1360946745562
35	H1	60	S4	ES	1488.16568047337
35	H1	60	S1	MG	167.868852459016
35	H1	60	S2	MG	145.464480874317
35	H1	60	S3	MG	231.03825136612
35	H1	60	S4	MG	206.120218579235
35	H1	60	S1	LM	320.384615384615
35	H1	60	S2	LM	103.269230769231
35	H1	60	S3	LM	161.153846153846
35	H1	60	S4	LM	386.153846153846
35	H2	80	S1	RF	186.693548387097
35	H2	80	S2	RF	88.7096774193548
35	H2	80	S3	RF	7.5268817204301
35	H2	80	S4	RF	291.801075268817
35	H2	80	S1	BB	284.682080924855
35	H2	80	S2	BB	1039.3063583815
35	H2	80	S3	BB	705.202312138728
35	H2	80	S4	BB	271.965317919075
35	H2	80	S1	FCU	-74.3824201755033
35	H2	80	S2	FCU	15.5372022712189
35	H2	80	S3	FCU	-2.94963498267089

Continued on next page

Participant_ID	Seat_Height	Seat_Height_pct_PH	Stage	Muscle	Delta_RMS_pct
35	H2	80	S4	FCU	-71.3811665806357
35	H2	80	S1	ES	485.962877030162
35	H2	80	S2	ES	1133.99071925754
35	H2	80	S3	ES	555.220417633411
35	H2	80	S4	ES	732.598607888631
35	H2	80	S1	MG	43.943661971831
35	H2	80	S2	MG	176.713615023474
35	H2	80	S3	MG	14.4600938967136
35	H2	80	S4	MG	109.577464788732
35	H2	80	S1	LM	329.609929078014
35	H2	80	S2	LM	126.595744680851
35	H2	80	S3	LM	77.8368794326241
35	H2	80	S4	LM	348.581560283688
35	H3	100	S1	RF	246.341463414634
35	H3	100	S2	RF	278.861788617886
35	H3	100	S3	RF	123.577235772358
35	H3	100	S4	RF	417.479674796748
35	H3	100	S1	BB	336.785714285714
35	H3	100	S2	BB	1363.92857142857
35	H3	100	S3	BB	1190.35714285714
35	H3	100	S4	BB	384.285714285714
35	H3	100	S1	FCU	-87.9244524814145
35	H3	100	S2	FCU	5.63257651865247
35	H3	100	S3	FCU	21.6864242180698
35	H3	100	S4	FCU	-86.0022771415177
35	H3	100	S1	ES	1310.68965517241
35	H3	100	S2	ES	199.310344827586
35	H3	100	S3	ES	175.51724137931
35	H3	100	S4	ES	1785.86206896552
35	H3	100	S1	MG	28.1132075471698
35	H3	100	S2	MG	174.622641509434
35	H3	100	S3	MG	137.924528301887
35	H3	100	S4	MG	76.3207547169812
35	H3	100	S1	LM	165.464632454924
35	H3	100	S2	LM	153.536754507628
35	H3	100	S3	LM	201.386962552011
35	H3	100	S4	LM	281.969486823856
35	H4	120	S1	RF	18.5595567867036
35	H4	120	S2	RF	-41.5512465373961
35	H4	120	S3	RF	15.4432132963989
35	H4	120	S4	RF	72.0221606648199
35	H4	120	S1	BB	389.795918367347
35	H4	120	S2	BB	1431.12244897959
35	H4	120	S3	BB	890.816326530612
35	H4	120	S4	BB	322.959183673469
35	H4	120	S1	FCU	-84.8875997099347
35	H4	120	S2	FCU	3.41551849166063
35	H4	120	S3	FCU	7.1211022480058
35	H4	120	S4	FCU	-88.4844089920232
35	H4	120	S1	ES	1054.30711610487
35	H4	120	S2	ES	276.029962546816
35	H4	120	S3	ES	365.917602996255
35	H4	120	S4	ES	1527.71535580524
35	H4	120	S1	MG	23.2943469785575
35	H4	120	S2	MG	250.584795321637
35	H4	120	S3	MG	-15.3021442495127

Continued on next page

Participant_ID	Seat_Height	Seat_Height_pct_PH	Stage	Muscle	Delta_RMS_pct
35	H4	120	S4	MG	107.699805068226
35	H4	120	S1	LM	137.339055793991
35	H4	120	S2	LM	158.512160228898
35	H4	120	S3	LM	483.834048640916
35	H4	120	S4	LM	212.160228898426

REBA

Seat_Height	Seat_Height_pct_PH	Stage	n	REBA_Mean	REBA_Median	REBA_Q1	REBA_Q3	Low_n	Low_pct	Medium_n	Medium_pct	High_n	High_pct	Very_High_n	Very_High_pct
H1	60	S1	35	6.57	6	6	7	0	0	31	0.886	4	0.114	0	0
H1	60	S2	35	7.49	8	8	8	1	0.029	7	0.2	27	0.771	0	0
H1	60	S3	35	6.2	6	6	6	0	0	35	1	0	0	0	0
H1	60	S4	35	5.74	6	4.5	6	0	0	31	0.886	4	0.114	0	0
H2	80	S1	35	6.2	6	6	7	0	0	33	0.943	2	0.057	0	0
H2	80	S2	35	7.86	8	8	8	0	0	3	0.086	32	0.914	0	0
H2	80	S3	35	5.77	6	6	6	0	0	35	1	0	0	0	0
H2	80	S4	35	4.89	4	4	6	3	0.086	32	0.914	0	0	0	0
H3	100	S1	35	6.23	6	6	7	0	0	35	1	0	0	0	0
H3	100	S2	35	8.49	9	8	9	0	0	1	0.029	34	0.971	0	0
H3	100	S3	35	5.6	6	5.5	6	0	0	35	1	0	0	0	0
H3	100	S4	35	4.6	4	4	6	4	0.114	31	0.886	0	0	0	0
H4	120	S1	35	5.31	6	4	6	0	0	35	1	0	0	0	0
H4	120	S2	35	8.74	9	8.5	9	0	0	0	0	35	1	0	0
H4	120	S3	35	4.94	5	4	6	1	0.029	34	0.971	0	0	0	0
H4	120	S4	35	4.63	4	4	5.5	2	0.057	33	0.943	0	0	0	0

Comfort

Participant_ID	Seat_Height	Seat_Height_pct_PH	Stage	Trial	Comfort_Rating
1	H1	60	S1	1	1
1	H1	60	S1	2	1
2	H1	60	S1	1	2
2	H1	60	S1	2	1
3	H1	60	S1	1	-3
3	H1	60	S1	2	-3
4	H1	60	S1	1	-3
4	H1	60	S1	2	-3
5	H1	60	S1	1	2
5	H1	60	S1	2	2
6	H1	60	S1	1	-3
6	H1	60	S1	2	-2
7	H1	60	S1	1	-3
7	H1	60	S1	2	-3
8	H1	60	S1	1	-3
8	H1	60	S1	2	-3
9	H1	60	S1	1	-3
9	H1	60	S1	2	-3
10	H1	60	S1	1	-2
10	H1	60	S1	2	-1
11	H1	60	S1	1	-3
11	H1	60	S1	2	-3
12	H1	60	S1	1	-2
12	H1	60	S1	2	-2
13	H1	60	S1	1	-3
13	H1	60	S1	2	-3
14	H1	60	S1	1	-3
14	H1	60	S1	2	-3
15	H1	60	S1	1	2
15	H1	60	S1	2	2
16	H1	60	S1	1	-1
16	H1	60	S1	2	-1
17	H1	60	S1	1	-2
17	H1	60	S1	2	-1
18	H1	60	S1	1	-1
18	H1	60	S1	2	-1
19	H1	60	S1	1	-1
19	H1	60	S1	2	-1
20	H1	60	S1	1	-3
20	H1	60	S1	2	-3
21	H1	60	S1	1	-3
21	H1	60	S1	2	-3
22	H1	60	S1	1	-3
22	H1	60	S1	2	-3
23	H1	60	S1	1	-3
23	H1	60	S1	2	-2
24	H1	60	S1	1	-2
24	H1	60	S1	2	-2
25	H1	60	S1	1	0
25	H1	60	S1	2	0
26	H1	60	S1	1	-3

Continued on next page

Participant_ID	Seat_Height	Seat_Height_pct_PH	Stage	Trial	Comfort_Rating
26	H1	60	S1	2	-3
27	H1	60	S1	1	-2
27	H1	60	S1	2	-2
28	H1	60	S1	1	-3
28	H1	60	S1	2	-3
29	H1	60	S1	1	-2
29	H1	60	S1	2	-3
30	H1	60	S1	1	-3
30	H1	60	S1	2	-3
31	H1	60	S1	1	1
31	H1	60	S1	2	1
32	H1	60	S1	1	2
32	H1	60	S1	2	2
33	H1	60	S1	1	-3
33	H1	60	S1	2	-3
34	H1	60	S1	1	-3
34	H1	60	S1	2	-3
35	H1	60	S1	1	2
35	H1	60	S1	2	2
1	H1	60	S2	1	3
1	H1	60	S2	2	3
2	H1	60	S2	1	2
2	H1	60	S2	2	2
3	H1	60	S2	1	2
3	H1	60	S2	2	1
4	H1	60	S2	1	3
4	H1	60	S2	2	2
5	H1	60	S2	1	3
5	H1	60	S2	2	3
6	H1	60	S2	1	3
6	H1	60	S2	2	3
7	H1	60	S2	1	1
7	H1	60	S2	2	1
8	H1	60	S2	1	3
8	H1	60	S2	2	3
9	H1	60	S2	1	-3
9	H1	60	S2	2	-3
10	H1	60	S2	1	0
10	H1	60	S2	2	0
11	H1	60	S2	1	3
11	H1	60	S2	2	3
12	H1	60	S2	1	2
12	H1	60	S2	2	2
13	H1	60	S2	1	1
13	H1	60	S2	2	1
14	H1	60	S2	1	3
14	H1	60	S2	2	3
15	H1	60	S2	1	2
15	H1	60	S2	2	2
16	H1	60	S2	1	3
16	H1	60	S2	2	3
17	H1	60	S2	1	3
17	H1	60	S2	2	2
18	H1	60	S2	1	2
18	H1	60	S2	2	2
19	H1	60	S2	1	-2

Continued on next page

Participant_ID	Seat_Height	Seat_Height_pct_PH	Stage	Trial	Comfort_Rating
19	H1	60	S2	2	-2
20	H1	60	S2	1	1
20	H1	60	S2	2	1
21	H1	60	S2	1	1
21	H1	60	S2	2	1
22	H1	60	S2	1	1
22	H1	60	S2	2	1
23	H1	60	S2	1	3
23	H1	60	S2	2	3
24	H1	60	S2	1	-2
24	H1	60	S2	2	-2
25	H1	60	S2	1	-1
25	H1	60	S2	2	0
26	H1	60	S2	1	2
26	H1	60	S2	2	2
27	H1	60	S2	1	-2
27	H1	60	S2	2	-2
28	H1	60	S2	1	-2
28	H1	60	S2	2	-2
29	H1	60	S2	1	-1
29	H1	60	S2	2	-1
30	H1	60	S2	1	-1
30	H1	60	S2	2	-1
31	H1	60	S2	1	-1
31	H1	60	S2	2	-1
32	H1	60	S2	1	2
32	H1	60	S2	2	2
33	H1	60	S2	1	2
33	H1	60	S2	2	2
34	H1	60	S2	1	-1
34	H1	60	S2	2	-1
35	H1	60	S2	1	2
35	H1	60	S2	2	2
1	H1	60	S3	1	2
1	H1	60	S3	2	2
2	H1	60	S3	1	-1
2	H1	60	S3	2	0
3	H1	60	S3	1	1
3	H1	60	S3	2	1
4	H1	60	S3	1	1
4	H1	60	S3	2	1
5	H1	60	S3	1	2
5	H1	60	S3	2	2
6	H1	60	S3	1	-1
6	H1	60	S3	2	-1
7	H1	60	S3	1	-2
7	H1	60	S3	2	-2
8	H1	60	S3	1	2
8	H1	60	S3	2	2
9	H1	60	S3	1	-1
9	H1	60	S3	2	-1
10	H1	60	S3	1	2
10	H1	60	S3	2	2
11	H1	60	S3	1	-3
11	H1	60	S3	2	-3
12	H1	60	S3	1	0

Continued on next page

Participant_ID	Seat_Height	Seat_Height_pct_PH	Stage	Trial	Comfort_Rating
12	H1	60	S3	2	0
13	H1	60	S3	1	-1
13	H1	60	S3	2	-1
14	H1	60	S3	1	2
14	H1	60	S3	2	2
15	H1	60	S3	1	-1
15	H1	60	S3	2	0
16	H1	60	S3	1	-2
16	H1	60	S3	2	-2
17	H1	60	S3	1	-2
17	H1	60	S3	2	-2
18	H1	60	S3	1	-3
18	H1	60	S3	2	-2
19	H1	60	S3	1	2
19	H1	60	S3	2	2
20	H1	60	S3	1	-2
20	H1	60	S3	2	-2
21	H1	60	S3	1	-2
21	H1	60	S3	2	-2
22	H1	60	S3	1	0
22	H1	60	S3	2	1
23	H1	60	S3	1	0
23	H1	60	S3	2	0
24	H1	60	S3	1	-1
24	H1	60	S3	2	-1
25	H1	60	S3	1	2
25	H1	60	S3	2	2
26	H1	60	S3	1	-2
26	H1	60	S3	2	-2
27	H1	60	S3	1	-2
27	H1	60	S3	2	-2
28	H1	60	S3	1	2
28	H1	60	S3	2	2
29	H1	60	S3	1	-3
29	H1	60	S3	2	-3
30	H1	60	S3	1	-2
30	H1	60	S3	2	-2
31	H1	60	S3	1	-2
31	H1	60	S3	2	-2
32	H1	60	S3	1	-2
32	H1	60	S3	2	-2
33	H1	60	S3	1	-3
33	H1	60	S3	2	-2
34	H1	60	S3	1	3
34	H1	60	S3	2	3
35	H1	60	S3	1	-1
35	H1	60	S3	2	-1
1	H1	60	S4	1	1
1	H1	60	S4	2	1
2	H1	60	S4	1	2
2	H1	60	S4	2	2
3	H1	60	S4	1	-3
3	H1	60	S4	2	-3
4	H1	60	S4	1	-3
4	H1	60	S4	2	-3
5	H1	60	S4	1	3

Continued on next page

Participant_ID	Seat_Height	Seat_Height_pct_PH	Stage	Trial	Comfort_Rating
5	H1	60	S4	2	3
6	H1	60	S4	1	-3
6	H1	60	S4	2	-2
7	H1	60	S4	1	-3
7	H1	60	S4	2	-3
8	H1	60	S4	1	-3
8	H1	60	S4	2	-3
9	H1	60	S4	1	-3
9	H1	60	S4	2	-3
10	H1	60	S4	1	-2
10	H1	60	S4	2	-2
11	H1	60	S4	1	-3
11	H1	60	S4	2	-3
12	H1	60	S4	1	-2
12	H1	60	S4	2	-2
13	H1	60	S4	1	-3
13	H1	60	S4	2	-2
14	H1	60	S4	1	-3
14	H1	60	S4	2	-3
15	H1	60	S4	1	2
15	H1	60	S4	2	2
16	H1	60	S4	1	-2
16	H1	60	S4	2	-2
17	H1	60	S4	1	-2
17	H1	60	S4	2	-2
18	H1	60	S4	1	-1
18	H1	60	S4	2	-1
19	H1	60	S4	1	-1
19	H1	60	S4	2	-1
20	H1	60	S4	1	-3
20	H1	60	S4	2	-3
21	H1	60	S4	1	-3
21	H1	60	S4	2	-3
22	H1	60	S4	1	-3
22	H1	60	S4	2	-3
23	H1	60	S4	1	-3
23	H1	60	S4	2	-3
24	H1	60	S4	1	-2
24	H1	60	S4	2	-2
25	H1	60	S4	1	0
25	H1	60	S4	2	0
26	H1	60	S4	1	-2
26	H1	60	S4	2	-1
27	H1	60	S4	1	-3
27	H1	60	S4	2	-3
28	H1	60	S4	1	-2
28	H1	60	S4	2	-2
29	H1	60	S4	1	-3
29	H1	60	S4	2	-3
30	H1	60	S4	1	-3
30	H1	60	S4	2	-3
31	H1	60	S4	1	-1
31	H1	60	S4	2	-1
32	H1	60	S4	1	-1
32	H1	60	S4	2	-1
33	H1	60	S4	1	-3

Continued on next page

Participant_ID	Seat_Height	Seat_Height_pct_PH	Stage	Trial	Comfort_Rating
33	H1	60	S4	2	-3
34	H1	60	S4	1	-3
34	H1	60	S4	2	-3
35	H1	60	S4	1	-3
35	H1	60	S4	2	-2
1	H2	80	S1	1	2
1	H2	80	S1	2	2
2	H2	80	S1	1	0
2	H2	80	S1	2	0
3	H2	80	S1	1	2
3	H2	80	S1	2	2
4	H2	80	S1	1	1
4	H2	80	S1	2	1
5	H2	80	S1	1	-1
5	H2	80	S1	2	-1
6	H2	80	S1	1	1
6	H2	80	S1	2	1
7	H2	80	S1	1	-1
7	H2	80	S1	2	-1
8	H2	80	S1	1	2
8	H2	80	S1	2	2
9	H2	80	S1	1	1
9	H2	80	S1	2	1
10	H2	80	S1	1	1
10	H2	80	S1	2	1
11	H2	80	S1	1	1
11	H2	80	S1	2	0
12	H2	80	S1	1	2
12	H2	80	S1	2	2
13	H2	80	S1	1	1
13	H2	80	S1	2	1
14	H2	80	S1	1	2
14	H2	80	S1	2	2
15	H2	80	S1	1	2
15	H2	80	S1	2	2
16	H2	80	S1	1	2
16	H2	80	S1	2	2
17	H2	80	S1	1	2
17	H2	80	S1	2	2
18	H2	80	S1	1	3
18	H2	80	S1	2	3
19	H2	80	S1	1	-1
19	H2	80	S1	2	-1
20	H2	80	S1	1	3
20	H2	80	S1	2	3
21	H2	80	S1	1	2
21	H2	80	S1	2	2
22	H2	80	S1	1	1
22	H2	80	S1	2	1
23	H2	80	S1	1	1
23	H2	80	S1	2	1
24	H2	80	S1	1	2
24	H2	80	S1	2	1
25	H2	80	S1	1	2
25	H2	80	S1	2	2
26	H2	80	S1	1	1

Continued on next page

Participant_ID	Seat_Height	Seat_Height_pct_PH	Stage	Trial	Comfort_Rating
26	H2	80	S1	2	1
27	H2	80	S1	1	-1
27	H2	80	S1	2	-1
28	H2	80	S1	1	1
28	H2	80	S1	2	1
29	H2	80	S1	1	-1
29	H2	80	S1	2	-1
30	H2	80	S1	1	2
30	H2	80	S1	2	2
31	H2	80	S1	1	1
31	H2	80	S1	2	0
32	H2	80	S1	1	2
32	H2	80	S1	2	2
33	H2	80	S1	1	1
33	H2	80	S1	2	1
34	H2	80	S1	1	2
34	H2	80	S1	2	2
35	H2	80	S1	1	2
35	H2	80	S1	2	2
1	H2	80	S2	1	2
1	H2	80	S2	2	2
2	H2	80	S2	1	1
2	H2	80	S2	2	1
3	H2	80	S2	1	0
3	H2	80	S2	2	0
4	H2	80	S2	1	3
4	H2	80	S2	2	3
5	H2	80	S2	1	2
5	H2	80	S2	2	2
6	H2	80	S2	1	1
6	H2	80	S2	2	1
7	H2	80	S2	1	-3
7	H2	80	S2	2	-3
8	H2	80	S2	1	1
8	H2	80	S2	2	1
9	H2	80	S2	1	-1
9	H2	80	S2	2	-1
10	H2	80	S2	1	0
10	H2	80	S2	2	0
11	H2	80	S2	1	-2
11	H2	80	S2	2	-2
12	H2	80	S2	1	2
12	H2	80	S2	2	2
13	H2	80	S2	1	-1
13	H2	80	S2	2	-1
14	H2	80	S2	1	1
14	H2	80	S2	2	1
15	H2	80	S2	1	0
15	H2	80	S2	2	0
16	H2	80	S2	1	2
16	H2	80	S2	2	1
17	H2	80	S2	1	-1
17	H2	80	S2	2	-1
18	H2	80	S2	1	2
18	H2	80	S2	2	2
19	H2	80	S2	1	0

Continued on next page

Participant_ID	Seat_Height	Seat_Height_pct_PH	Stage	Trial	Comfort_Rating
19	H2	80	S2	2	0
20	H2	80	S2	1	3
20	H2	80	S2	2	3
21	H2	80	S2	1	-2
21	H2	80	S2	2	-2
22	H2	80	S2	1	2
22	H2	80	S2	2	2
23	H2	80	S2	1	0
23	H2	80	S2	2	0
24	H2	80	S2	1	0
24	H2	80	S2	2	0
25	H2	80	S2	1	3
25	H2	80	S2	2	2
26	H2	80	S2	1	2
26	H2	80	S2	2	2
27	H2	80	S2	1	1
27	H2	80	S2	2	1
28	H2	80	S2	1	1
28	H2	80	S2	2	1
29	H2	80	S2	1	1
29	H2	80	S2	2	1
30	H2	80	S2	1	2
30	H2	80	S2	2	2
31	H2	80	S2	1	2
31	H2	80	S2	2	2
32	H2	80	S2	1	2
32	H2	80	S2	2	2
33	H2	80	S2	1	-1
33	H2	80	S2	2	-1
34	H2	80	S2	1	3
34	H2	80	S2	2	2
35	H2	80	S2	1	2
35	H2	80	S2	2	2
1	H2	80	S3	1	3
1	H2	80	S3	2	3
2	H2	80	S3	1	3
2	H2	80	S3	2	3
3	H2	80	S3	1	3
3	H2	80	S3	2	3
4	H2	80	S3	1	0
4	H2	80	S3	2	0
5	H2	80	S3	1	1
5	H2	80	S3	2	1
6	H2	80	S3	1	3
6	H2	80	S3	2	3
7	H2	80	S3	1	-2
7	H2	80	S3	2	-2
8	H2	80	S3	1	2
8	H2	80	S3	2	1
9	H2	80	S3	1	1
9	H2	80	S3	2	1
10	H2	80	S3	1	-1
10	H2	80	S3	2	-1
11	H2	80	S3	1	2
11	H2	80	S3	2	2
12	H2	80	S3	1	1

Continued on next page

Participant_ID	Seat_Height	Seat_Height_pct_PH	Stage	Trial	Comfort_Rating
12	H2	80	S3	2	1
13	H2	80	S3	1	-1
13	H2	80	S3	2	-1
14	H2	80	S3	1	2
14	H2	80	S3	2	2
15	H2	80	S3	1	2
15	H2	80	S3	2	2
16	H2	80	S3	1	2
16	H2	80	S3	2	2
17	H2	80	S3	1	2
17	H2	80	S3	2	1
18	H2	80	S3	1	2
18	H2	80	S3	2	2
19	H2	80	S3	1	1
19	H2	80	S3	2	1
20	H2	80	S3	1	2
20	H2	80	S3	2	2
21	H2	80	S3	1	0
21	H2	80	S3	2	0
22	H2	80	S3	1	-1
22	H2	80	S3	2	-1
23	H2	80	S3	1	3
23	H2	80	S3	2	3
24	H2	80	S3	1	1
24	H2	80	S3	2	1
25	H2	80	S3	1	2
25	H2	80	S3	2	1
26	H2	80	S3	1	-1
26	H2	80	S3	2	-1
27	H2	80	S3	1	2
27	H2	80	S3	2	2
28	H2	80	S3	1	2
28	H2	80	S3	2	2
29	H2	80	S3	1	2
29	H2	80	S3	2	2
30	H2	80	S3	1	2
30	H2	80	S3	2	2
31	H2	80	S3	1	3
31	H2	80	S3	2	3
32	H2	80	S3	1	1
32	H2	80	S3	2	1
33	H2	80	S3	1	1
33	H2	80	S3	2	2
34	H2	80	S3	1	2
34	H2	80	S3	2	2
35	H2	80	S3	1	3
35	H2	80	S3	2	3
1	H2	80	S4	1	2
1	H2	80	S4	2	2
2	H2	80	S4	1	0
2	H2	80	S4	2	0
3	H2	80	S4	1	2
3	H2	80	S4	2	2
4	H2	80	S4	1	1
4	H2	80	S4	2	1
5	H2	80	S4	1	-1

Continued on next page

Participant_ID	Seat_Height	Seat_Height_pct_PH	Stage	Trial	Comfort_Rating
5	H2	80	S4	2	-1
6	H2	80	S4	1	1
6	H2	80	S4	2	1
7	H2	80	S4	1	-2
7	H2	80	S4	2	-2
8	H2	80	S4	1	1
8	H2	80	S4	2	2
9	H2	80	S4	1	2
9	H2	80	S4	2	2
10	H2	80	S4	1	1
10	H2	80	S4	2	1
11	H2	80	S4	1	1
11	H2	80	S4	2	1
12	H2	80	S4	1	2
12	H2	80	S4	2	2
13	H2	80	S4	1	1
13	H2	80	S4	2	1
14	H2	80	S4	1	1
14	H2	80	S4	2	1
15	H2	80	S4	1	2
15	H2	80	S4	2	2
16	H2	80	S4	1	2
16	H2	80	S4	2	2
17	H2	80	S4	1	2
17	H2	80	S4	2	3
18	H2	80	S4	1	3
18	H2	80	S4	2	3
19	H2	80	S4	1	-1
19	H2	80	S4	2	-1
20	H2	80	S4	1	3
20	H2	80	S4	2	3
21	H2	80	S4	1	2
21	H2	80	S4	2	2
22	H2	80	S4	1	2
22	H2	80	S4	2	2
23	H2	80	S4	1	1
23	H2	80	S4	2	1
24	H2	80	S4	1	2
24	H2	80	S4	2	3
25	H2	80	S4	1	2
25	H2	80	S4	2	2
26	H2	80	S4	1	1
26	H2	80	S4	2	1
27	H2	80	S4	1	-2
27	H2	80	S4	2	-2
28	H2	80	S4	1	1
28	H2	80	S4	2	1
29	H2	80	S4	1	2
29	H2	80	S4	2	2
30	H2	80	S4	1	1
30	H2	80	S4	2	1
31	H2	80	S4	1	1
31	H2	80	S4	2	2
32	H2	80	S4	1	2
32	H2	80	S4	2	2
33	H2	80	S4	1	2

Continued on next page

Participant_ID	Seat_Height	Seat_Height_pct_PH	Stage	Trial	Comfort_Rating
33	H2	80	S4	2	2
34	H2	80	S4	1	2
34	H2	80	S4	2	2
35	H2	80	S4	1	3
35	H2	80	S4	2	3
1	H3	100	S1	1	3
1	H3	100	S1	2	3
2	H3	100	S1	1	3
2	H3	100	S1	2	3
3	H3	100	S1	1	-1
3	H3	100	S1	2	-1
4	H3	100	S1	1	-1
4	H3	100	S1	2	-1
5	H3	100	S1	1	-2
5	H3	100	S1	2	-1
6	H3	100	S1	1	-2
6	H3	100	S1	2	-2
7	H3	100	S1	1	-2
7	H3	100	S1	2	-2
8	H3	100	S1	1	-1
8	H3	100	S1	2	-1
9	H3	100	S1	1	-2
9	H3	100	S1	2	-2
10	H3	100	S1	1	1
10	H3	100	S1	2	1
11	H3	100	S1	1	-1
11	H3	100	S1	2	-1
12	H3	100	S1	1	-1
12	H3	100	S1	2	-1
13	H3	100	S1	1	2
13	H3	100	S1	2	2
14	H3	100	S1	1	-1
14	H3	100	S1	2	-1
15	H3	100	S1	1	0
15	H3	100	S1	2	0
16	H3	100	S1	1	2
16	H3	100	S1	2	1
17	H3	100	S1	1	-1
17	H3	100	S1	2	-1
18	H3	100	S1	1	2
18	H3	100	S1	2	2
19	H3	100	S1	1	1
19	H3	100	S1	2	1
20	H3	100	S1	1	-2
20	H3	100	S1	2	-1
21	H3	100	S1	1	1
21	H3	100	S1	2	1
22	H3	100	S1	1	-2
22	H3	100	S1	2	-2
23	H3	100	S1	1	-1
23	H3	100	S1	2	-1
24	H3	100	S1	1	0
24	H3	100	S1	2	0
25	H3	100	S1	1	0
25	H3	100	S1	2	0
26	H3	100	S1	1	-2

Continued on next page

Participant_ID	Seat_Height	Seat_Height_pct_PH	Stage	Trial	Comfort_Rating
26	H3	100	S1	2	-2
27	H3	100	S1	1	-2
27	H3	100	S1	2	-1
28	H3	100	S1	1	-1
28	H3	100	S1	2	-1
29	H3	100	S1	1	-2
29	H3	100	S1	2	-2
30	H3	100	S1	1	1
30	H3	100	S1	2	1
31	H3	100	S1	1	-1
31	H3	100	S1	2	-1
32	H3	100	S1	1	2
32	H3	100	S1	2	2
33	H3	100	S1	1	-1
33	H3	100	S1	2	-1
34	H3	100	S1	1	0
34	H3	100	S1	2	0
35	H3	100	S1	1	2
35	H3	100	S1	2	2
1	H3	100	S2	1	3
1	H3	100	S2	2	3
2	H3	100	S2	1	2
2	H3	100	S2	2	2
3	H3	100	S2	1	2
3	H3	100	S2	2	2
4	H3	100	S2	1	2
4	H3	100	S2	2	1
5	H3	100	S2	1	2
5	H3	100	S2	2	2
6	H3	100	S2	1	2
6	H3	100	S2	2	2
7	H3	100	S2	1	-2
7	H3	100	S2	2	-2
8	H3	100	S2	1	2
8	H3	100	S2	2	2
9	H3	100	S2	1	2
9	H3	100	S2	2	2
10	H3	100	S2	1	-1
10	H3	100	S2	2	-1
11	H3	100	S2	1	3
11	H3	100	S2	2	3
12	H3	100	S2	1	2
12	H3	100	S2	2	2
13	H3	100	S2	1	-2
13	H3	100	S2	2	-2
14	H3	100	S2	1	3
14	H3	100	S2	2	3
15	H3	100	S2	1	2
15	H3	100	S2	2	2
16	H3	100	S2	1	3
16	H3	100	S2	2	2
17	H3	100	S2	1	3
17	H3	100	S2	2	3
18	H3	100	S2	1	3
18	H3	100	S2	2	3
19	H3	100	S2	1	1

Continued on next page

Participant_ID	Seat_Height	Seat_Height_pct_PH	Stage	Trial	Comfort_Rating
19	H3	100	S2	2	1
20	H3	100	S2	1	2
20	H3	100	S2	2	2
21	H3	100	S2	1	-1
21	H3	100	S2	2	-1
22	H3	100	S2	1	0
22	H3	100	S2	2	0
23	H3	100	S2	1	2
23	H3	100	S2	2	2
24	H3	100	S2	1	2
24	H3	100	S2	2	2
25	H3	100	S2	1	2
25	H3	100	S2	2	2
26	H3	100	S2	1	2
26	H3	100	S2	2	1
27	H3	100	S2	1	2
27	H3	100	S2	2	2
28	H3	100	S2	1	2
28	H3	100	S2	2	2
29	H3	100	S2	1	2
29	H3	100	S2	2	2
30	H3	100	S2	1	2
30	H3	100	S2	2	2
31	H3	100	S2	1	3
31	H3	100	S2	2	3
32	H3	100	S2	1	2
32	H3	100	S2	2	2
33	H3	100	S2	1	1
33	H3	100	S2	2	1
34	H3	100	S2	1	2
34	H3	100	S2	2	2
35	H3	100	S2	1	3
35	H3	100	S2	2	2
1	H3	100	S3	1	2
1	H3	100	S3	2	2
2	H3	100	S3	1	2
2	H3	100	S3	2	2
3	H3	100	S3	1	1
3	H3	100	S3	2	1
4	H3	100	S3	1	3
4	H3	100	S3	2	3
5	H3	100	S3	1	1
5	H3	100	S3	2	1
6	H3	100	S3	1	2
6	H3	100	S3	2	2
7	H3	100	S3	1	-2
7	H3	100	S3	2	-2
8	H3	100	S3	1	2
8	H3	100	S3	2	2
9	H3	100	S3	1	0
9	H3	100	S3	2	0
10	H3	100	S3	1	1
10	H3	100	S3	2	1
11	H3	100	S3	1	-1
11	H3	100	S3	2	-1
12	H3	100	S3	1	1

Continued on next page

Participant_ID	Seat_Height	Seat_Height_pct_PH	Stage	Trial	Comfort_Rating
12	H3	100	S3	2	1
13	H3	100	S3	1	0
13	H3	100	S3	2	0
14	H3	100	S3	1	2
14	H3	100	S3	2	2
15	H3	100	S3	1	0
15	H3	100	S3	2	0
16	H3	100	S3	1	2
16	H3	100	S3	2	2
17	H3	100	S3	1	2
17	H3	100	S3	2	2
18	H3	100	S3	1	2
18	H3	100	S3	2	2
19	H3	100	S3	1	0
19	H3	100	S3	2	0
20	H3	100	S3	1	3
20	H3	100	S3	2	2
21	H3	100	S3	1	-2
21	H3	100	S3	2	-2
22	H3	100	S3	1	2
22	H3	100	S3	2	2
23	H3	100	S3	1	1
23	H3	100	S3	2	1
24	H3	100	S3	1	1
24	H3	100	S3	2	1
25	H3	100	S3	1	3
25	H3	100	S3	2	3
26	H3	100	S3	1	0
26	H3	100	S3	2	0
27	H3	100	S3	1	2
27	H3	100	S3	2	2
28	H3	100	S3	1	1
28	H3	100	S3	2	1
29	H3	100	S3	1	1
29	H3	100	S3	2	1
30	H3	100	S3	1	2
30	H3	100	S3	2	2
31	H3	100	S3	1	2
31	H3	100	S3	2	2
32	H3	100	S3	1	2
32	H3	100	S3	2	1
33	H3	100	S3	1	-1
33	H3	100	S3	2	-1
34	H3	100	S3	1	2
34	H3	100	S3	2	2
35	H3	100	S3	1	2
35	H3	100	S3	2	2
1	H3	100	S4	1	3
1	H3	100	S4	2	3
2	H3	100	S4	1	3
2	H3	100	S4	2	3
3	H3	100	S4	1	-1
3	H3	100	S4	2	-1
4	H3	100	S4	1	-1
4	H3	100	S4	2	-1
5	H3	100	S4	1	-2

Continued on next page

Participant_ID	Seat_Height	Seat_Height_pct_PH	Stage	Trial	Comfort_Rating
5	H3	100	S4	2	-2
6	H3	100	S4	1	-2
6	H3	100	S4	2	-2
7	H3	100	S4	1	-2
7	H3	100	S4	2	-2
8	H3	100	S4	1	-1
8	H3	100	S4	2	-1
9	H3	100	S4	1	-2
9	H3	100	S4	2	-1
10	H3	100	S4	1	1
10	H3	100	S4	2	1
11	H3	100	S4	1	-1
11	H3	100	S4	2	-1
12	H3	100	S4	1	-1
12	H3	100	S4	2	-1
13	H3	100	S4	1	2
13	H3	100	S4	2	2
14	H3	100	S4	1	-1
14	H3	100	S4	2	-1
15	H3	100	S4	1	1
15	H3	100	S4	2	1
16	H3	100	S4	1	2
16	H3	100	S4	2	2
17	H3	100	S4	1	-1
17	H3	100	S4	2	0
18	H3	100	S4	1	2
18	H3	100	S4	2	2
19	H3	100	S4	1	1
19	H3	100	S4	2	1
20	H3	100	S4	1	-2
20	H3	100	S4	2	-2
21	H3	100	S4	1	1
21	H3	100	S4	2	1
22	H3	100	S4	1	-2
22	H3	100	S4	2	-2
23	H3	100	S4	1	-1
23	H3	100	S4	2	-1
24	H3	100	S4	1	0
24	H3	100	S4	2	0
25	H3	100	S4	1	0
25	H3	100	S4	2	0
26	H3	100	S4	1	-2
26	H3	100	S4	2	-2
27	H3	100	S4	1	-2
27	H3	100	S4	2	-2
28	H3	100	S4	1	-1
28	H3	100	S4	2	-1
29	H3	100	S4	1	-2
29	H3	100	S4	2	-1
30	H3	100	S4	1	1
30	H3	100	S4	2	1
31	H3	100	S4	1	2
31	H3	100	S4	2	2
32	H3	100	S4	1	-1
32	H3	100	S4	2	-1
33	H3	100	S4	1	1

Continued on next page

Participant_ID	Seat_Height	Seat_Height_pct_PH	Stage	Trial	Comfort_Rating
33	H3	100	S4	2	1
34	H3	100	S4	1	2
34	H3	100	S4	2	2
35	H3	100	S4	1	-1
35	H3	100	S4	2	-1
1	H4	120	S1	1	1
1	H4	120	S1	2	1
2	H4	120	S1	1	-1
2	H4	120	S1	2	-1
3	H4	120	S1	1	2
3	H4	120	S1	2	2
4	H4	120	S1	1	1
4	H4	120	S1	2	1
5	H4	120	S1	1	1
5	H4	120	S1	2	1
6	H4	120	S1	1	2
6	H4	120	S1	2	2
7	H4	120	S1	1	2
7	H4	120	S1	2	2
8	H4	120	S1	1	2
8	H4	120	S1	2	2
9	H4	120	S1	1	1
9	H4	120	S1	2	1
10	H4	120	S1	1	2
10	H4	120	S1	2	2
11	H4	120	S1	1	1
11	H4	120	S1	2	2
12	H4	120	S1	1	1
12	H4	120	S1	2	1
13	H4	120	S1	1	0
13	H4	120	S1	2	0
14	H4	120	S1	1	2
14	H4	120	S1	2	2
15	H4	120	S1	1	2
15	H4	120	S1	2	2
16	H4	120	S1	1	-1
16	H4	120	S1	2	-1
17	H4	120	S1	1	1
17	H4	120	S1	2	1
18	H4	120	S1	1	-2
18	H4	120	S1	2	-2
19	H4	120	S1	1	2
19	H4	120	S1	2	2
20	H4	120	S1	1	-2
20	H4	120	S1	2	-2
21	H4	120	S1	1	2
21	H4	120	S1	2	2
22	H4	120	S1	1	2
22	H4	120	S1	2	2
23	H4	120	S1	1	-1
23	H4	120	S1	2	-1
24	H4	120	S1	1	-1
24	H4	120	S1	2	-1
25	H4	120	S1	1	0
25	H4	120	S1	2	0
26	H4	120	S1	1	2

Continued on next page

Participant_ID	Seat_Height	Seat_Height_pct_PH	Stage	Trial	Comfort_Rating
26	H4	120	S1	2	1
27	H4	120	S1	1	2
27	H4	120	S1	2	2
28	H4	120	S1	1	1
28	H4	120	S1	2	1
29	H4	120	S1	1	2
29	H4	120	S1	2	2
30	H4	120	S1	1	1
30	H4	120	S1	2	1
31	H4	120	S1	1	-1
31	H4	120	S1	2	-1
32	H4	120	S1	1	1
32	H4	120	S1	2	1
33	H4	120	S1	1	-2
33	H4	120	S1	2	-2
34	H4	120	S1	1	2
34	H4	120	S1	2	2
35	H4	120	S1	1	-2
35	H4	120	S1	2	-2
1	H4	120	S2	1	1
1	H4	120	S2	2	1
2	H4	120	S2	1	-1
2	H4	120	S2	2	0
3	H4	120	S2	1	0
3	H4	120	S2	2	0
4	H4	120	S2	1	0
4	H4	120	S2	2	0
5	H4	120	S2	1	1
5	H4	120	S2	2	1
6	H4	120	S2	1	-2
6	H4	120	S2	2	-2
7	H4	120	S2	1	-2
7	H4	120	S2	2	-2
8	H4	120	S2	1	1
8	H4	120	S2	2	1
9	H4	120	S2	1	-2
9	H4	120	S2	2	-2
10	H4	120	S2	1	1
10	H4	120	S2	2	1
11	H4	120	S2	1	-2
11	H4	120	S2	2	-2
12	H4	120	S2	1	1
12	H4	120	S2	2	1
13	H4	120	S2	1	-2
13	H4	120	S2	2	-2
14	H4	120	S2	1	1
14	H4	120	S2	2	1
15	H4	120	S2	1	-2
15	H4	120	S2	2	-2
16	H4	120	S2	1	-2
16	H4	120	S2	2	-2
17	H4	120	S2	1	-3
17	H4	120	S2	2	-3
18	H4	120	S2	1	-3
18	H4	120	S2	2	-3
19	H4	120	S2	1	1

Continued on next page

Participant_ID	Seat_Height	Seat_Height_pct_PH	Stage	Trial	Comfort_Rating
19	H4	120	S2	2	1
20	H4	120	S2	1	-2
20	H4	120	S2	2	-2
21	H4	120	S2	1	-3
21	H4	120	S2	2	-3
22	H4	120	S2	1	1
22	H4	120	S2	2	1
23	H4	120	S2	1	-1
23	H4	120	S2	2	-1
24	H4	120	S2	1	-2
24	H4	120	S2	2	-2
25	H4	120	S2	1	1
25	H4	120	S2	2	0
26	H4	120	S2	1	-2
26	H4	120	S2	2	-2
27	H4	120	S2	1	-3
27	H4	120	S2	2	-3
28	H4	120	S2	1	1
28	H4	120	S2	2	1
29	H4	120	S2	1	-2
29	H4	120	S2	2	-1
30	H4	120	S2	1	-3
30	H4	120	S2	2	-3
31	H4	120	S2	1	-3
31	H4	120	S2	2	-3
32	H4	120	S2	1	-2
32	H4	120	S2	2	-2
33	H4	120	S2	1	-3
33	H4	120	S2	2	-3
34	H4	120	S2	1	3
34	H4	120	S2	2	3
35	H4	120	S2	1	-2
35	H4	120	S2	2	-2
1	H4	120	S3	1	3
1	H4	120	S3	2	3
2	H4	120	S3	1	3
2	H4	120	S3	2	3
3	H4	120	S3	1	3
3	H4	120	S3	2	3
4	H4	120	S3	1	2
4	H4	120	S3	2	2
5	H4	120	S3	1	2
5	H4	120	S3	2	2
6	H4	120	S3	1	2
6	H4	120	S3	2	2
7	H4	120	S3	1	1
7	H4	120	S3	2	1
8	H4	120	S3	1	3
8	H4	120	S3	2	3
9	H4	120	S3	1	-2
9	H4	120	S3	2	-2
10	H4	120	S3	1	1
10	H4	120	S3	2	1
11	H4	120	S3	1	2
11	H4	120	S3	2	2
12	H4	120	S3	1	2

Continued on next page

Participant_ID	Seat_Height	Seat_Height_pct_PH	Stage	Trial	Comfort_Rating
12	H4	120	S3	2	2
13	H4	120	S3	1	0
13	H4	120	S3	2	1
14	H4	120	S3	1	2
14	H4	120	S3	2	2
15	H4	120	S3	1	2
15	H4	120	S3	2	2
16	H4	120	S3	1	3
16	H4	120	S3	2	3
17	H4	120	S3	1	2
17	H4	120	S3	2	2
18	H4	120	S3	1	1
18	H4	120	S3	2	1
19	H4	120	S3	1	-2
19	H4	120	S3	2	-2
20	H4	120	S3	1	1
20	H4	120	S3	2	1
21	H4	120	S3	1	1
21	H4	120	S3	2	1
22	H4	120	S3	1	1
22	H4	120	S3	2	1
23	H4	120	S3	1	3
23	H4	120	S3	2	3
24	H4	120	S3	1	-1
24	H4	120	S3	2	-1
25	H4	120	S3	1	0
25	H4	120	S3	2	0
26	H4	120	S3	1	2
26	H4	120	S3	2	2
27	H4	120	S3	1	-1
27	H4	120	S3	2	-1
28	H4	120	S3	1	-2
28	H4	120	S3	2	-2
29	H4	120	S3	1	-1
29	H4	120	S3	2	-1
30	H4	120	S3	1	1
30	H4	120	S3	2	1
31	H4	120	S3	1	0
31	H4	120	S3	2	0
32	H4	120	S3	1	0
32	H4	120	S3	2	1
33	H4	120	S3	1	2
33	H4	120	S3	2	2
34	H4	120	S3	1	-2
34	H4	120	S3	2	-2
35	H4	120	S3	1	2
35	H4	120	S3	2	2
1	H4	120	S4	1	1
1	H4	120	S4	2	1
2	H4	120	S4	1	-1
2	H4	120	S4	2	-1
3	H4	120	S4	1	2
3	H4	120	S4	2	2
4	H4	120	S4	1	1
4	H4	120	S4	2	1
5	H4	120	S4	1	1

Continued on next page

Participant_ID	Seat_Height	Seat_Height_pct_PH	Stage	Trial	Comfort_Rating
5	H4	120	S4	2	1
6	H4	120	S4	1	2
6	H4	120	S4	2	2
7	H4	120	S4	1	2
7	H4	120	S4	2	1
8	H4	120	S4	1	2
8	H4	120	S4	2	2
9	H4	120	S4	1	2
9	H4	120	S4	2	2
10	H4	120	S4	1	2
10	H4	120	S4	2	2
11	H4	120	S4	1	1
11	H4	120	S4	2	1
12	H4	120	S4	1	1
12	H4	120	S4	2	1
13	H4	120	S4	1	1
13	H4	120	S4	2	1
14	H4	120	S4	1	2
14	H4	120	S4	2	2
15	H4	120	S4	1	2
15	H4	120	S4	2	2
16	H4	120	S4	1	-1
16	H4	120	S4	2	-1
17	H4	120	S4	1	1
17	H4	120	S4	2	1
18	H4	120	S4	1	-2
18	H4	120	S4	2	-2
19	H4	120	S4	1	2
19	H4	120	S4	2	2
20	H4	120	S4	1	-1
20	H4	120	S4	2	-1
21	H4	120	S4	1	2
21	H4	120	S4	2	2
22	H4	120	S4	1	2
22	H4	120	S4	2	1
23	H4	120	S4	1	-1
23	H4	120	S4	2	-1
24	H4	120	S4	1	-1
24	H4	120	S4	2	-1
25	H4	120	S4	1	0
25	H4	120	S4	2	0
26	H4	120	S4	1	1
26	H4	120	S4	2	1
27	H4	120	S4	1	2
27	H4	120	S4	2	2
28	H4	120	S4	1	2
28	H4	120	S4	2	2
29	H4	120	S4	1	2
29	H4	120	S4	2	2
30	H4	120	S4	1	2
30	H4	120	S4	2	2
31	H4	120	S4	1	2
31	H4	120	S4	2	2
32	H4	120	S4	1	2
32	H4	120	S4	2	2
33	H4	120	S4	1	-1

Continued on next page

Participant_ID	Seat_Height	Seat_Height_pct_PH	Stage	Trial	Comfort_Rating
33	H4	120	S4	2	-1
34	H4	120	S4	1	1
34	H4	120	S4	2	1
35	H4	120	S4	1	-2
35	H4	120	S4	2	-2

Data Dictionary

Sheet	Variable	Definition
sEMG_Primary	Participant_ID	De-identified study participant code (1–35).
sEMG_Primary	Seat_Height	H1–H4 seat-height condition code.
sEMG_Primary	Seat_Height_pct_PH	Seat height expressed as percentage of individual popliteal height: 60, 80, 100, or 120.
sEMG_Primary	Stage	S1–S4 task-stage code.
sEMG_Primary	Muscle	BB, FCU, ES, LM, RF, or MG.
sEMG_Primary	Delta_RMS_pct	Stage-level relative RMS change (%) used in the primary sEMG analysis.
REBA	Seat_Height	H1–H4 seat-height condition code.
REBA	Seat_Height_pct_PH	Seat height expressed as percentage of individual popliteal height.
REBA	Stage	S1–S4 task-stage code.
REBA	n	Number of final condition-level REBA observations summarized in the row (35).
REBA	REBA_Mean	Mean final REBA score for the Seat Height × Stage condition, matching Supplementary Table S8.
REBA	REBA_Median	Median final REBA score, matching Supplementary Table S5 Panel B.
REBA	REBA_Q1 / REBA_Q3	First and third quartiles of final REBA scores, matching Supplementary Table S5 Panel B.
REBA	Low_n / Low_pct	Count and percentage with REBA scores 2–3.
REBA	Medium_n / Medium_pct	Count and percentage with REBA scores 4–7.
REBA	High_n / High_pct	Count and percentage with REBA scores 8–10.
REBA	Very_High_n / Very_High_pct	Count and percentage with REBA scores 11–15.
Comfort	Participant_ID	De-identified study participant code (1–35).
Comfort	Seat_Height	H1–H4 seat-height condition code.
Comfort	Seat_Height_pct_PH	Seat height expressed as percentage of individual popliteal height.
Comfort	Stage	S1–S4 task-stage code.
Comfort	Trial	Repeated trial number (1 or 2).
Comfort	Comfort_Rating	Stage-specific subjective comfort rating on the numerical scale from -3 to +3.
